# High Performance Aluminum Ion Batteries Enabled by the Coordination Between Vanadium‐Based PBAs Cathode and Aqueous Eutectic Electrolyte

**DOI:** 10.1002/advs.202511274

**Published:** 2025-08-13

**Authors:** Wanchang Feng, Boman Li, Guoqiang Yuan, Yawei Li, Yanfei Zhang, Meng Du, Yichun Su, Yijian Tang, Haotian Yue, Yuxin Li, Mohsen Shakouri, Hsiao‐Chien Chen, Wenting Li, Zheng Liu, Huan Pang

**Affiliations:** ^1^ School of Chemistry and Chemical Engineering Yangzhou University Yangzhou Jiangsu 225002 P. R. China; ^2^ School of Chemistry and Chemical Engineering Chongqing University of Science & Technology Chongqing 401331 P. R. China; ^3^ School of Environmental Science Nanjing Xiaozhuang University Nanjing P. R. China; ^4^ Canadian Light Source Inc. University of Saskatchewan Saskatoon S7N 2V3 Canada; ^5^ Center for Reliability Science and Technologies Kidney Research Center Department of Nephrology Chang Gung Memorial Hospital Chang Gung University Linkou, Taoyuan 333 Taiwan P. R. China; ^6^ Key Laboratory of Advanced Energy Materials Chemistry (Ministry of Education) Nankai University Tianjin 300071 P. R. China

**Keywords:** aqueous aluminum‐ion batteries, eutectic electrolyte, Prussian blue analogues, vanadium

## Abstract

The practical application of aqueous aluminumion batteries (AAlBs) faced with critical challenges, such as low rate performance and poor cycling stability due to the absence of ideal cathode materials. To address the bottlenecks of low electrochemical activity, structural instability, and narrow voltage window in Prussian blue analogues (PBAs) for AAIBs, this study develops a universal synthesis strategy integrating acid‐assisted method with ligand modulation to prepare high‐performance vanadium‐based PBAs (V‐PBAs) cathodes. Through precise coordination environment control, V and Fe/Co/Ni synergistically enhance multi‐electron redox activity. Density functional theory calculations reveal that the Fe‐doped VFePBA exhibits a narrow bandgap (0.479 eV) and low Al^3+^ migration energy barrier (0.586 eV), enabling rapid ion transport. Combined with an Al_2_(SO_4_)_3_‐urea eutectic electrolyte (AU15) that expands the operational voltage window to 0.1–2.0 V, the optimized Zn||AU15||VFePBA system achieves a high specific capacity of 161.37 mAh g^−1^ at 0.1 A g^−1^. In‐situ characterizations confirm a suppressed structural distortion via Al‐O coordination and a capacitive‐dominated charge storage mechanism. Flexible pouch cells demonstrate stable operation under mechanical bending and practical device powering capabilities. This work provides a novel paradigm for the systematic assembly of advanced safe, and low‐cost post‐lithium energy storage systems.

## Graphical abstract

The study proposes a universal strategy for the synthesis of high‐performance vanadium‐based PBAs (V‐PBAs) cathodes for aluminum ion batteries. It is find that the doped transition metals (Fe/Co/Ni) at carbon‐coordinated sites can markedly enhance the multi‐electron redox activity and inhibit the V dissolution, structural collapse, and other degradation issues.

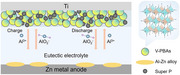

## 1 Introduction

As the most representative coordination polymer, Prussian blue analogues (PBAs) have attracted continuous research interest in energy storage chemistry due to their unique open framework structure and adjustable coordination environment.^[^ ^1^, ^2^, ^3^, ^4^ ^]^ Particularly under China's “Dual Carbon Strategic Goals” policy, great efforts have been devoted to searching for “post‐lithium” energy storage systems characterized by abundant resources and significant cost advantages,^[^ ^5^, ^6^ ^]^ such as sodium‐ion batteries (SIBs),^[^ ^7^, ^8^ ^]^ potassium‐ion batteries (KIBs),^[^ ^9^, ^10^ ^]^ and zinc‐ion batteries (ZIBs).^[^ ^11^, ^12^, ^13^, ^14^ ^]^ In this technological revolution, PBAs exhibit great potential as cathode materials due to their specific capacity and high electrochemical windows.^[^ ^15^, ^16^, ^17^ ^]^ Very recently (May 2025), Contemporary Amperex Technology Co. Limited (CATL) proposed the new commercial application of sodium‐ion batteries using PBAs as cathode materials, fully validating the engineering feasibility of PBAs. However, the current battery systems are all assembled with organic electrolyte, which are easily flammable, resulting in a significant safety hazard.^[^ ^18^, ^19^, ^20^, ^21^ ^]^ Therefore, the exploitation of alternative energy storage systems with intrinsic safety and high performance based on PBAs cathode is significant.

Aqueous aluminum‐ion batteries (AAIBs) show high theoretical capacity of 2980 mAh g^−1^ (volumetric capacity of 8046 mAh cm^−3^) and low cost due to the aluminum's three‐electron redox reaction and crustal abundance (8.2 wt.%), revealing significant potential for Lithium‐ion batteries (LIBs) alternative.^[^ ^22^, ^23^, ^24^ ^]^ However, the practical applications of PBAs as cathode for AAIBs still face several crucial challenges, such as poor electrochemical activity and low electronic conductivity, resulting in unsatisfactory practical specific capacity and rate performance.^[^ ^25^ ^]^ More critically, the strong polarization of high‐charge‐density of Al^3+^ during insertion/extraction processes exacerbates lattice stress, inducing structural collapse, while irreversible dissolution of transition metal ions further aggravates capacity fading.^[^ ^26^ ^]^ Although introducing electrochemically active metals like Mn, Ni, and Co to construct multi‐metal systems can obviously enhance specific capacity, the severe volume effects can inevitably cause lattice distortion and rapid decay of cycling stability.^[^ ^27^ ^]^ To address these issues, the researchers have employed multi‐dimensional modification strategies, including entropy engineering regulation,^[^ ^28^ ^]^ defect engineering optimization, and heterostructure construction,^[^ ^29^ ^]^ but the synergistically improvement of rate capability and long‐term cycling stability of PBAs cathode for practical AAIBs are still faced with technical bottlenecks.

Due to the multi‐electron redox reaction of multivalent vanadium centers, vanadium‐based PBAs (V‐PBAs), can provide multiple charge storage sites during charge/discharge process, theoretically revealing superior electrochemical performance than other PBAs.^[^ ^30^ ^]^ However, the complex valence transitions of vanadium is a double‐edged sword and can inevitably exacerbate material dissolution tendencies. More disappointedly, the controllable synthesis of V‐PBAs via traditional coprecipitation methods is faced with inherent difficulty, and the conventional metal doping strategies tend to occupy vanadium sites and reduce effective capacity.^[^ ^31^, ^32^ ^]^ The corresponding research on V‐PBAs in AAIBs systems remains unexplored. Recently research revealed that the synergistic coordination with transition metals like Fe and Co significantly enhances the reactivity of vanadium sites, though conventional doping strategies still suffer from structural defects caused by competitive occupation of active sites.^[^ ^33^ ^]^ This discovery highlights the need to develop novel site‐selective modification technologies that precisely regulate coordination environments to optimize the stability and reaction efficiency of vanadium active centers. In addition, the practical advancement of AAIBs faces dual challenges in electrolyte systems that water molecule decomposition causes the narrow electrochemical window and the irreversible corrosion of aluminum anodes.^[^ ^34^, ^35^, ^36^ ^]^ To address these challenges, aqueous eutectic electrolyte systems have emerged as a cutting‐edge research frontier due to their unique component synergy.^[^ ^37^, ^38^ ^]^ They retain the intrinsic safety of aqueous systems while achieving an ionic liquid‐like wide voltage window through stable solvation structures, providing an ideal reaction environment for high‐capacity cathode materials like V‐PBAs with multi‐step redox characteristics.^[^ ^39^ ^]^ Thus, developing universal synthesis strategies for V‐PBAs cathode materials and designing compatible electrolytes (featuring both wide voltage windows and interfacial stability) tailored to their framework characteristics will systematically overcome critical bottlenecks such as capacity fading and cycling lifespan in AAIBs.

Herein, we propose a universal acid‐assisted strategy for the preparation of series of V‐PBAs based crystals via an acid‐assisted strategy, as AAIBs cathode, greatly increasing the percentage of electrochemical active site while maintaining the entire crystal coordination structure. Particularly, the synergistic effect of V‐Fe double metal sites shows multiple electron redox process, effectively enhance the specific capacity. Density functional theory (DFT) and in‐situ characterizations verify that the electron conductivity and structural stability of V‐PBAs can be largely improved after doping of Fe atom at C‐coordinated sites. More significantly, when assembled with the aqueous eutectic electrolyte, the V dissolution, structural collapse, and other degradation issues are obviously inhibited, leading to a broaden working electrochemical window from 0.1 V to 2.0 V and higher capacity increases from 103.32 to 161.37 mAh g^−1^. In‐situ X‐ray Diffraction (XRD) patterns verify that the electrode exhibits significantly reduced configuration distortion in the eutectic electrolyte. Therefore, the assembled Zn||AU15||VFePBA shows excellent rate capability (161.37, 138.09, 120.59, 105.63, 93.14, 84.04, and 70.71 mAh g^−1^ at 0.1–0.8 A g^−1^), and ultra‐long cycling stability (100 mAh g^−1^ retention after 500 cycles at 0.5 A g^−1^), which is superior to most of the reported results. The systematic resolution strategy of ration design of PBA cathode with novelty electrolyte provides a new avenue for the further development of practical batteries in the post LIBs time.

## 2 Results and Discussion

**Figure 1:**
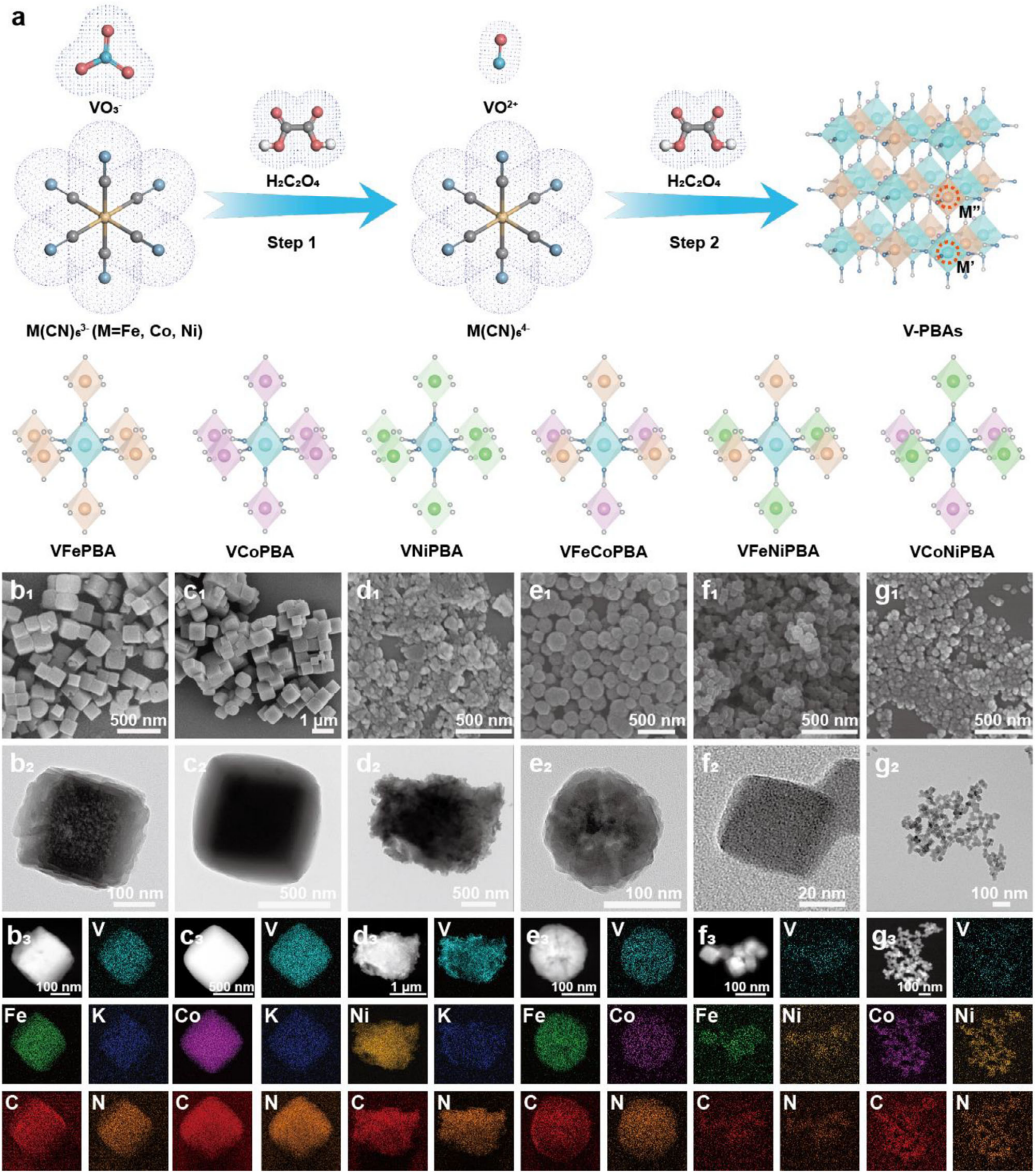
a) Synthesis and structural schematic. SEM, HRTEM, dark‐field images and elemental mapping of b1–b3) VFePBA. c1–c3) VCoPBA. d1–d3) VNiPBA. e1e3) VFeCoPBA. f1f3) VFeNiPBA. g1g3) VCoNiPBA.

To elucidate the inner effects of different metals on the preparation of V‐PBAs, we developed an acid‐assisted hydrothermal synthesis method (**Figure** **1**a; Figure S1, Supporting Information), realizing the precise control of V‐PBAs through the synergistic effects of bifunctional oxalic acid (H_2_C_2_O_4_). The key mechanism can bie attributed to the multifunction effect of H_2_C_2_O_4_, in which the H_2_C_2_O_4_ acts as a reducing agent in Step 1 to convert VO_3_^−^ into VO^2+^, followed by the weak acid dissociation in Step 2 to regulate crystal growth kinetics. Notably, VO_3_^−^ cannot directly coordinate with cyanide ligands, whereas the reduced VO^2+^ can spontaneously forms stable coordination bonds with ligands. This in‐situ slow‐release strategy effectively controls the nucleation rates, successfully yielding the uniformly dispersion of PBAs nanoparticles. By systematically adjusting the types and ratios of cyanometallate precursors (M(CN)_6_^3−^), we prepared a series of samples materials including VFePBA, VCoPBA, VNiPBA, and their ternary composites (VFeCoPBA, VFeNiPBA, VCoNiPBA). Fe, Co, and Ni sources are introduced as hexacyanometallate precursors, where their metal centers are already bound to the carbon atoms of cyanide ligands (CN−) through strong covalent bonds, forming highly stable [M″(CN)_6_] structural units. These units exhibit ligand rigidity (linear cyanide configuration with high‐strength triple bonds), coordination saturation (metal centers fully occupied by six cyanide carbon atoms), and kinetic inertness (high dissociation energy of coordination bonds), ensuring their chemical integrity throughout the hydrothermal synthesis. In these atomic scale, the M’ sites (coordinated with N in CN^−^) are exclusively occupied by V, while the M″ sites (coordinated with C in CN^−^) are occupied by other metals. This differs from conventional metal doping approaches, in which the introduced doping metals are tended to occupy M’ sites, inevitably reducing V content and compromising specific capacity. The unconventional regulation of doping metals in ligands can effectively avoids the occupation of M’ sites, enabling the successful preparation of V‐PBAs with highest V content. Scanning electron microscopy (SEM) and transmission electron microscopy (TEM) images of the synthesized V‐PBAs (Figure 1b1–g2) reveal the distinct morphologies, that VFePBA, VCoPBA, VFeNiPBA, and VCoNiPBA exhibit regular cubic shapes with average sizes of ≈200 nm, 800 nm, <50 nm, and <50 nm, respectively. In contrast, VNiPBA shows ultra‐small nanoparticles with agglomerated morphology in microscopy, while VFeCoPBA displays a spherical structure. Significant differences in grain sizes (VNiPBA < VFeNiPBA ≈ VCoNiPBA < VFePBA ≈ VFeCoPBA < VCoPBA) can be attributed to the gradual increased coordination capability of ligands (potassium nickel cyanide > potassium ferricyanide > potassium cobalt cyanide). Elemental mapping images (Figure 1b3–g3) confirms the uniform distribution of all elements. This method enables the universal synthesis of V‐PBAs with diverse metal compositions and ratios (Figures S2–S6, Supporting Information).

**Figure 2:**
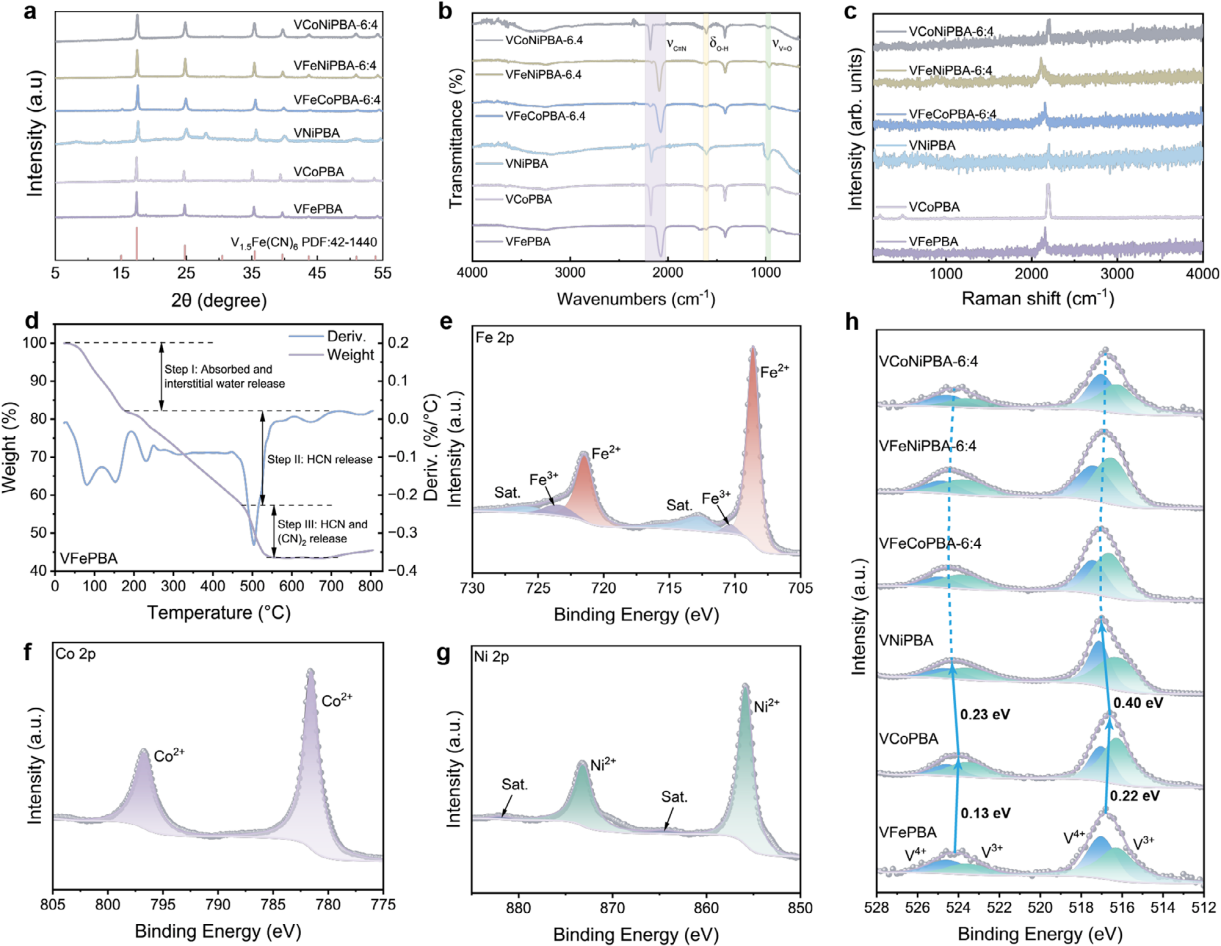
a) XRD patterns. b) FTIR spectra. c) Raman spectra. d) Thermogravimetric (TG) curve of VFePBA. e) Fe 2p XPS spectrum of VFePBA. f) Co 2p XPS spectrum of VCoPBA. g) Ni 2p XPS spectrum of VNiPBA. h) V 2p XPS spectrum.

**Figure** **2**a shows the XRD patterns of the prepared V‐PBAs. All samples exhibit specific diffraction peaks at ≈17.6°, 25.0°, and 35.8°, which can be well assigned to the (200), (220), and (400) crystal planes of PBAs, respectively, indicating the successful synthesis of series V‐PBAs.^[^ ^40^ ^]^ Moreover, when the metal ratios are rationally controlled, the XRD patterns shows no obvious changes, further indicating that the various metal doping of V‐PBAs with different percentage cannot break the coordination environment and entire crystal morphology (Figures S7–S9, Supporting Information).

As shown in Figure 2b and Figures S10–S12 (Supporting Information), The Fourier Transform infrared spectroscopy (FTIR) spectra exhibit distinct absorption peak in the range of 2000–2200 cm^−1^, corresponding to the coordination between transition metals and cyanide ligands (CN^−^), where CN^−^ acts as a σ‐donor. Notably, The ν(CN) vibrational frequency follows an inverse relationship with dopant electronegativity (Fe < Co < Ni): VFePBA exhibits the lowest frequency (2070 cm^−1^) due to Fe's lowest electronegativity weakening the σ‐donor effect, while higher Co/Ni proportions induce blue shifts (VCoPBA: 2174 cm^−1^; VNiPBA: 2171 cm^−1^). As the proportions of Co and Ni in V‐PBAs increase, the ν(CN) vibrational frequency shifts to higher wavenumbers. This ligand electronic modulation directly correlates with vanadium's oxidation state changes observed in X‐ray photoelectron spectroscopy (XPS). Raman spectral data further validated these coordination characteristics (Figure 2c; Figures S13–S15, Supporting Information). Thermogravimetric analysis (TGA) of VFePBA revealed three distinct weight‐loss stages, including the removal of interstitial and coordinated water (17.76%), followed by HCN volatilization, and finally the co‐release of HCN and CN_2_ (Figure 2d). XPS investigations elucidated the electronic interactions between V and incorporated metals (Fe, Co, Ni). Survey spectra confirmed the presence of V, O, C, N, and transition metals in all samples (Figure S16, Supporting Information). The V 2p XPS spectrum displayed peaks at 516.6 eV (2p_3/2_) and 523.8 eV (2p_1/2_), indicative of mixed V^4+^/V^3+^ states. Distinct Fe 2p_3_/_2_ (708.61 eV) and Fe 2p_1/2_ (721.34 eV), Co 2p_3/2_ (781.53 eV) and Co 2p_1/2_ (796.68 eV), and Ni 2p_3/2_ (855.75 eV) and Ni 2p_1/2_ (873.09 eV) signals confirmed the successful integration of these metals into the PBA framework (Figure 2e–g; Figures S17–S23, Supporting Information). Metal substitution induced notable shifts in V binding energies, with VCoPBA exhibiting 0.22 and 0.13 eV downward shifts in V 2p_3/2_ and 2p_1/2_ peaks compared to VFePBA (suggesting enhanced electron density at V sites), while VNiPBA showed upward shifts of 0.18 and 0.10 eV due to reduced coordination effects despite electron donation from Ni‐bound CN^−^ (Figure 2h; Figure S24, Supporting Information). The optimal V^4+^/V^3+^ ratio and strong electron delocalization in VFePBA facilitated abundant redox‐active sites, advantageous for electrochemical processes. N_2_ physisorption analysis revealed a hierarchical pore structure with dominant pore sizes below 1.0 nm and ≈1.5 nm (Figure S25, Supporting Information). These collective results demonstrate that Fe, Co, and Ni doping effectively modulates the electronic structure and redox properties of V centers in PBAs through charge redistribution and valence state adjustments.

**Figure 3:**
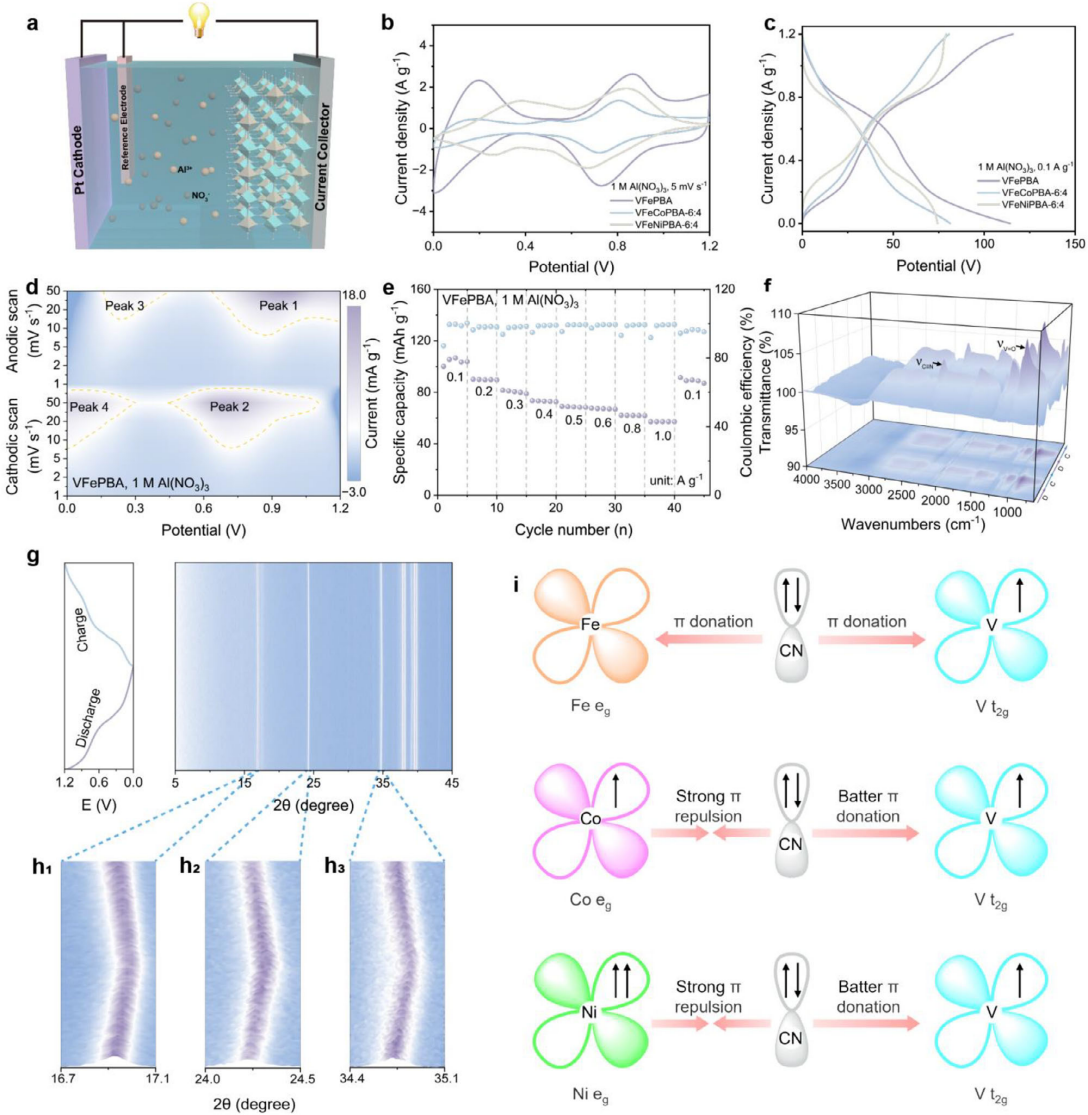
Electrochemical performance in aqueous electrolyte: a) Schematic diagram of the three‐electrode cell configuration. b) CV curves of samples with different elemental compositions. c) Charge/discharge curves of samples with different elemental compositions. d) CV curves of VFePBA at various scan rates. e) Rate performance of VFePBA. f) In‐situ FTIR spectra of VFePBA in 1 M Al(NO_3_)_3_ electrolyte. g) In‐situ XRD patterns of VFePBA and corresponding charge/discharge curves in 1 M Al(NO_3_)_3_ electrolyte. h1–h3) Magnified regions of in‐situ XRD patterns. i) Schematic illustration of intermetallic electron push‐pull interactions.

To investigate the impact of Fe, Co, and Ni incorporation on the electrochemical performance of V‐PBAs, the Al storage behavior of the materials was evaluated in a three‐electrode system using an aqueous electrolyte (1 M Al(NO_3_)_3_) (**Figure** **3**a). Cyclic voltammetry (CV) curves of all samples exhibited two redox couples corresponding to V^4+^/V^3+^ (≈0.8 V) and V^3+^/V^2+^ (≈0.2 V) transitions (Figure 3b; Figures S26 and S27, Supporting Information). Notably, the CV integral area decreased with increasing Co or Ni substitution in VFePBA, indicating reduced electrochemical activity. Galvanostatic charge‐discharge (GCD) profiles further corroborated this trend with all materials displayed two distinct plateaus within 0–1.2 V, and the specific capacity declined progressively with higher Co/Ni doping ratios (Figure 3c; Figures S28 and S29, Supporting Information), confirming the superior performance of pristine VFePBA. Kinetic analysis via variable scan rate CV revealed distinct charge storage mechanisms, with VFePBA exhibiting b‐values of 0.90 (Peak 1), 0.85 (Peak 2), and 0.59 (Peak 3), suggesting capacitive dominance for the first two peaks and diffusion control for the third. The capacitive contribution increased from 44.0% to 83.9% with higher scan rates (Figure 3d; Figures S30 and S31, Supporting Information). The CV curves, b values, and capacitive contribution ratios (including capacitive contribution ratios at different scan rates) of other samples showing in Figures S32–S47 (Supporting Information) further systematically illustrate the impact of metal content (particularly the Fe content) on the electrochemical performance of V‐PBAs. Crucially, the performance of different samples shows a positive correlation with Fe content, strongly supporting our central argument that Fe enhances the electrochemical performance of V‐PBAs. The high capacitive contribution rationalize the exceptional rate capability of VFePBA, which delivered specific capacities of 103.32, 89.73, 80.38, 73.29, 69.09, 67.01, 62.41, 57.44, and 90.85 mAh g^−1^ at current densities of 0.1, 0.2, 0.3, 0.4, 0.5, 0.6, 0.8, 1.0, and 0.1 A g^−1^, respectively, outperforming all doped counterparts (Figure 3e). The rate performance of selected additional samples is shown in Figures S48–S50 (Supporting Information). Comparative CV studies between room‐temperature and high‐temperature synthesized samples highlighted enhanced current response and larger CV areas for the latter (Figures S51 and S52, Supporting Information), while cycling stability tests (Figures S53–S55, Supporting Information) confirmed the superior durability of high‐temperature synthesized VFePBA. In‐situ FTIR spectroscopy during charge/discharge cycles revealed dynamic structural changes characterized by increased transmittance of ν_C≡N_ and ν_V = O_ bands during discharge (signifying weakened bond strengths due to Al^3+^ adsorption near C≡N and V = O sites), while reversed trends during charge indicated bond strength recovery upon Al^3+^ desorption (Figure 3f). In‐situ XRD analysis demonstrated lattice contraction during discharge, evidenced by shifts of (200), (220), and (400) diffraction peaks toward higher angles (16.9°, 24.2°, 34.7°), attributed to strong Al^3+^‐lattice interactions. Upon charging, peak reversibility confirmed elastic lattice expansion from Al^3+^ extraction (Figure 3g,h).^[^ ^28^, ^41^ ^]^ The charge transfer resistance decreases during the discharge process, facilitating efficient battery discharge, while the reverse trend is observed during charging, demonstrating excellent electrochemical reversibility (Figure S56, Supporting Information). Electronic structure analysis elucidated the synergistic effects in V‐C≡N‐M (M = Fe, Co, Ni) frameworks through CN− (as a strong‐field ligand) mediating metal d‐electron configuration‐dependent charge transfer dynamics. Fe^2+^ (3d^6^, t_2g_^6^), Co^2+^ (3d^7^, t_2g_^6^e_g_^1^), and Ni^2+^ (3d^8^, t_2g_^6^e_g_^2^) progressively enhance electron repulsion from their e_g_ orbitals toward CN^−^, thereby increasing electron density at V^4+^ (3d^1^, t_2g_^1^) and lowering its oxidation state.^[^ ^42^ ^]^ However, Ni(CN)_4_^2−^’s preference for four‐coordination (versus six‐coordination for Fe(CN)_6_^4−^/Co(CN)_6_^4−^) weakens its influence on V compared to Fe/Co, consistent with XPS observations (Figure 3i). These findings collectively establish that Fe, Co, and Ni doping modulates both electronic and structural properties of V‐PBAs, directly correlating with their aluminum storage performance.

VFePBA demonstrates broad applicability in other conventional aqueous electrolytes, including 1 M AlCl_3_ and 0.5 M Al_2_(SO_4_)_3_ (Figure S57, Supporting Information). In a coin cell, employing 1 M Al(OTF)_3_ + 0.2 M Mn(OTF)_2_ as the electrolyte and Al as the anode, the cycling stability was tested, demonstrating a cycle life of 300 cycles (Figure S58, Supporting Information). When the anode was replaced with Zn, the initial specific capacity increased to 120 mAh g^−1^ (Figure S59, Supporting Information).

**Figure 4:**
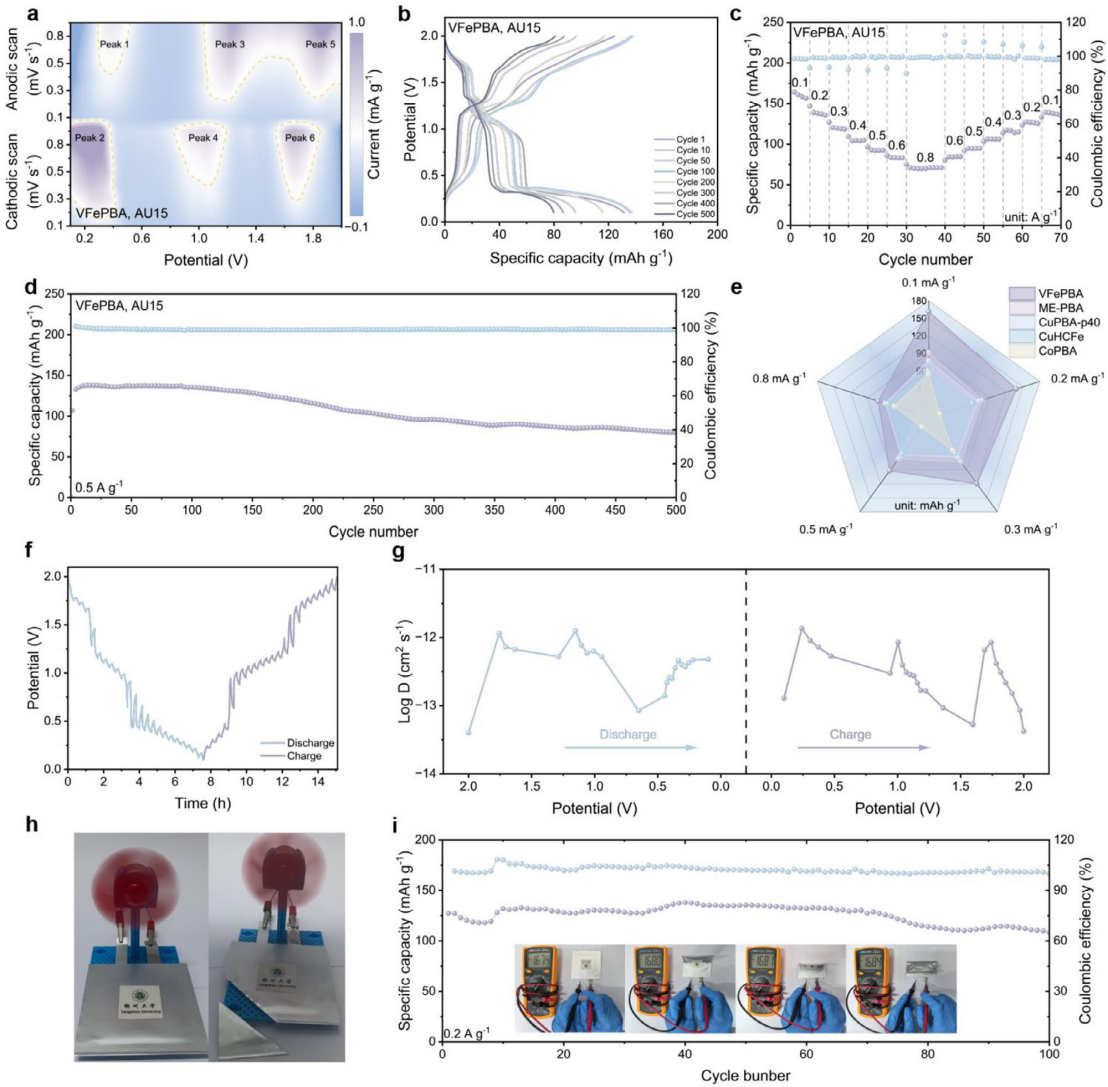
Electrochemical performance in AU15 electrolyte: a) CV curves of VFePBA at various scan rates. b) Charge/discharge curves of VFePBA. c) Capacitive contribution ratios of VFePBA at various scan rates. d) Long‐term cycling performance of VFePBA. e) Rate performance of VFePBA. f) GITT curves of VFePBA. g) Ionic conductivity of VFePBA calculated from GITT measurements. h) Pouch cell demonstration of VFePBA. i) Long‐term cycling performance of VFePBA‐based pouch cell and voltage variation under different bending angles.

The limited voltage window of conventional aqueous electrolytes in two‐electrode systems, where the Al^3+^ deposition potential exceeds the electrochemical stability range, leads to severe side reactions and restricts practical applications. To address this challenge, we developed a eutectic electrolyte (denoted as AU15, comprising Al_2_(SO_4_)_3_, urea, and H_2_O) to expand the operational voltage window to 0.1–2.0 V, while employing Zn as the anode to harness the AlZn alloying reaction during cycling, which effectively reduces the Al^3+^ deposition potential and enhances cycling stability. In the Zn||AU15||VFePBA battery system, CV reveals three distinct redox couples (**Figure** **4**a), including an additional high‐voltage redox pair at 1.7 V attributed to the Fe^3+^/Fe^2+^ process, absent in conventional aqueous electrolytes. Corresponding GCD profiles (Figure 4b) exhibit three voltage plateaus, consistent with the multi‐step redox reactions observed in CV, and deliver a specific capacity of 161.37 mAh g^−1^ owing to the expanded voltage window. Kinetic analysis via b‐value fitting demonstrates capacitive‐dominated behavior across all redox peaks, with b‐values as 0.71, 0.88, 0.83, 0.74, 0.99, 0.86 for Peak 1 to Peak 6. Capacitive contributions progressively increase from 49.8% at 0.1 mV s^−1^ to 75.2% at 1.0 mV s^−1^ (Figures S60 and S61, Supporting Information). The Zn||AU15||VFePBA configuration achieves reversible capacities of 161.37, 138.09, 120.59, 105.63, 93.14, 84.04, and 70.71 mAh g^−1^ at current densities of 0.1–0.8 A g^−1^, respectively (Figure 4c), and maintains 100 mAh g^−1^ after 500 cycles at 0.5 A g^−1^ (Figure 4d) The electrochemical impedance spectroscopy of the battery confirmed the low charge transfer resistance and favorable ion diffusion kinetics in the Zn||AU15||VFePBA battery (Figure S62, Supporting Information). Figure S63 (Supporting Information) presents the contact angle measurement results between the VFePBA electrode and electrolyte. The measured contact angle of 69.4° indicates moderate wettability on the electrode surface. This favorable wetting property facilitates the formation of uniform ion transport channels and effectively promotes the charge transfer process. As shown in Figure 4e, the performance comparison with other materials demonstrates that the VFePBA cathode exhibits the highest specific capacity across various current densities among all PBA cathodes.^[^ ^27^, ^43^, ^44^, ^45^, ^46^ ^]^ Galvanostatic intermittent titration technique (GITT) analysis (Figure 4f,g) further quantifies the Al^3+^ diffusion coefficient in VFePBA within the range of 5 × 10^−14^ to 2 × 10^−12^ cm^2^ s^−1^, governed by Fick's second law. Practical applicability is demonstrated through a flexible pouch cell that powers a miniaturized fan without short‐circuiting even after mechanical cutting (Figure 4h), while retaining 110 mAh g^−1^ after 100 cycles at 0.2 A g^−1^ and showing invariant open‐circuit voltage during repeated bending (Figure 4i; Figure S64, Supporting Information). These results collectively validate the synergistic electrolyte‐electrode design in enabling high‐capacity, stable, and safe AAIBs systems.

**Figure 5:**
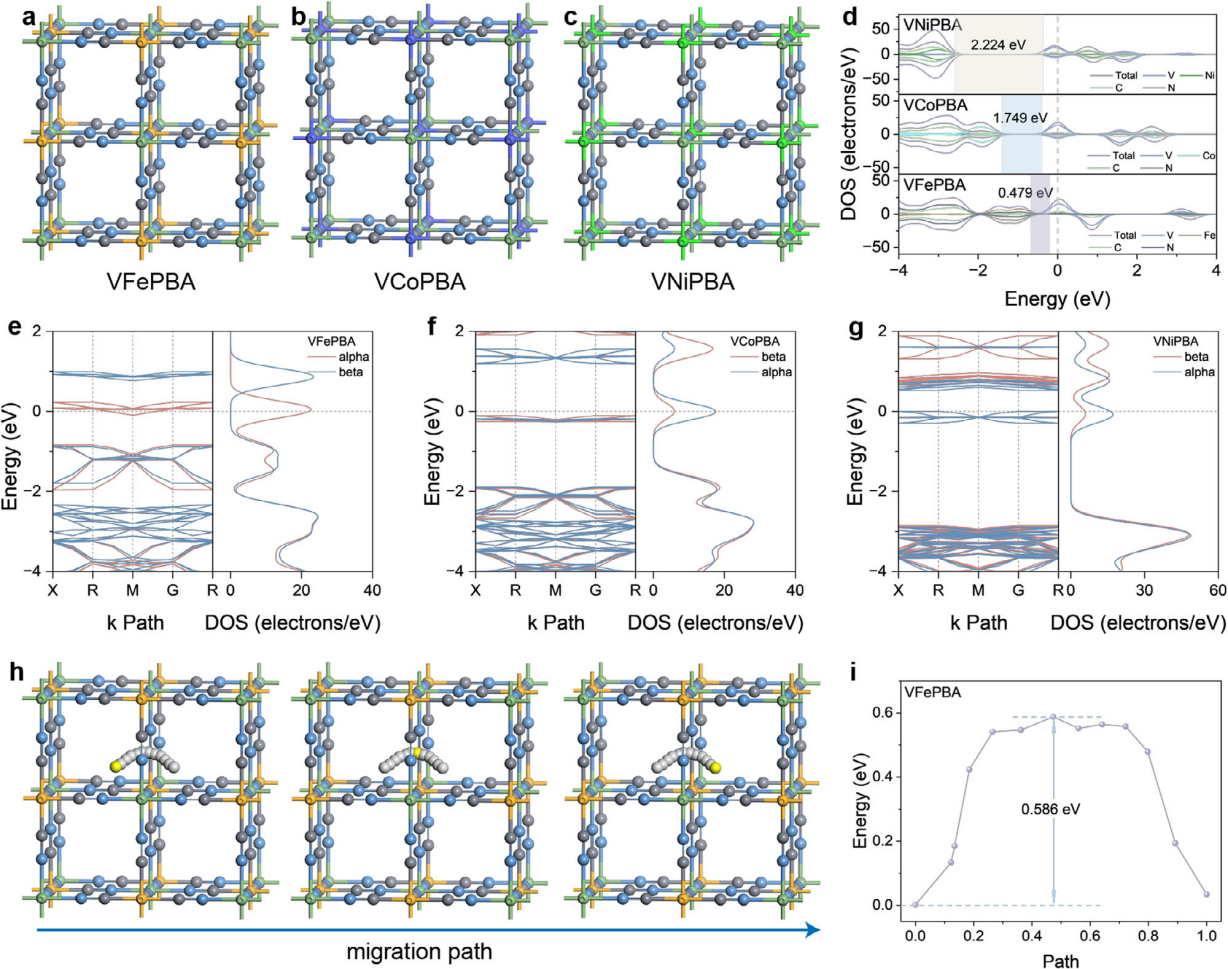
a) Structural schematic of VFePBA. b) Structural schematic of VCoPBA. c) Structural schematic of VNiPBA. d) Comparative density of states (DOS) diagrams of VFePBA, VCoPBA, and VNiPBA. e) Band structure diagram of VFePBA. f) Band structure diagram of VCoPBA. g) Band structure diagram of VNiPBA. h) Al^3+^ migration pathway in VFePBA. i) Migration energy barrier of Al^3+^ in VFePBA.

To clarify the structural evolution mechanism of V‐PBAs during aluminum ion storage, this study conducted systematic computational analysis using DFT. Taking VFePBA as a model system (initial structure shown in Figure S65, Supporting Information), structural characterization revealed that V occupies the M' sites (coordinated with cyanide nitrogen) and features adjacent pendant oxygen atoms (characterized by V = O bonds). Charge distribution analysis along the (101) crystal plane demonstrated that both oxygen atoms and cyanide groups act as negative charge centers, with the lone pair electrons of oxygen atoms oriented toward material channels (Figure S66, Supporting Information). Upon Al^3+^ intercalation, its high charge density promotes coordination with oxygen lone pairs, forming Al‐O composite structures (e.g., AlO^+^/AlO_2_^−^). This process leads to oxygen detachment from the lattice and effectively screens the charge effects of Al^3+^ (Figure S67, Supporting Information). Based on this mechanism, we constructed optimized structural models with oxygen removal (**Figure** **5**a–c) and introduced Fe, Co, and Ni at M″ sites (coordinated with cyanide carbon) for comparative investigation.

Electronic structure calculations showed that VFePBA, VCoPBA, and VNiPBA exhibit band gaps of 0.479, 1.749, and 2.224 eV, respectively (Figure 5d). Band structure analysis (Figure 5e–g) confirmed that VFePBA possesses the narrowest band gap due to Fe's d‐orbital electronic characteristics, consistent with its superior electrochemical activity. Further calculation of Al^3+^ migration pathways in VFePBA (Figure 5h) revealed an energy barrier of only 0.586 eV for diffusion along crystal channels (Figure 5i), indicating efficient ion transport pathways. These findings provide theoretical support for the observed high‐rate performance in experimental studies.

**Figure 6:**
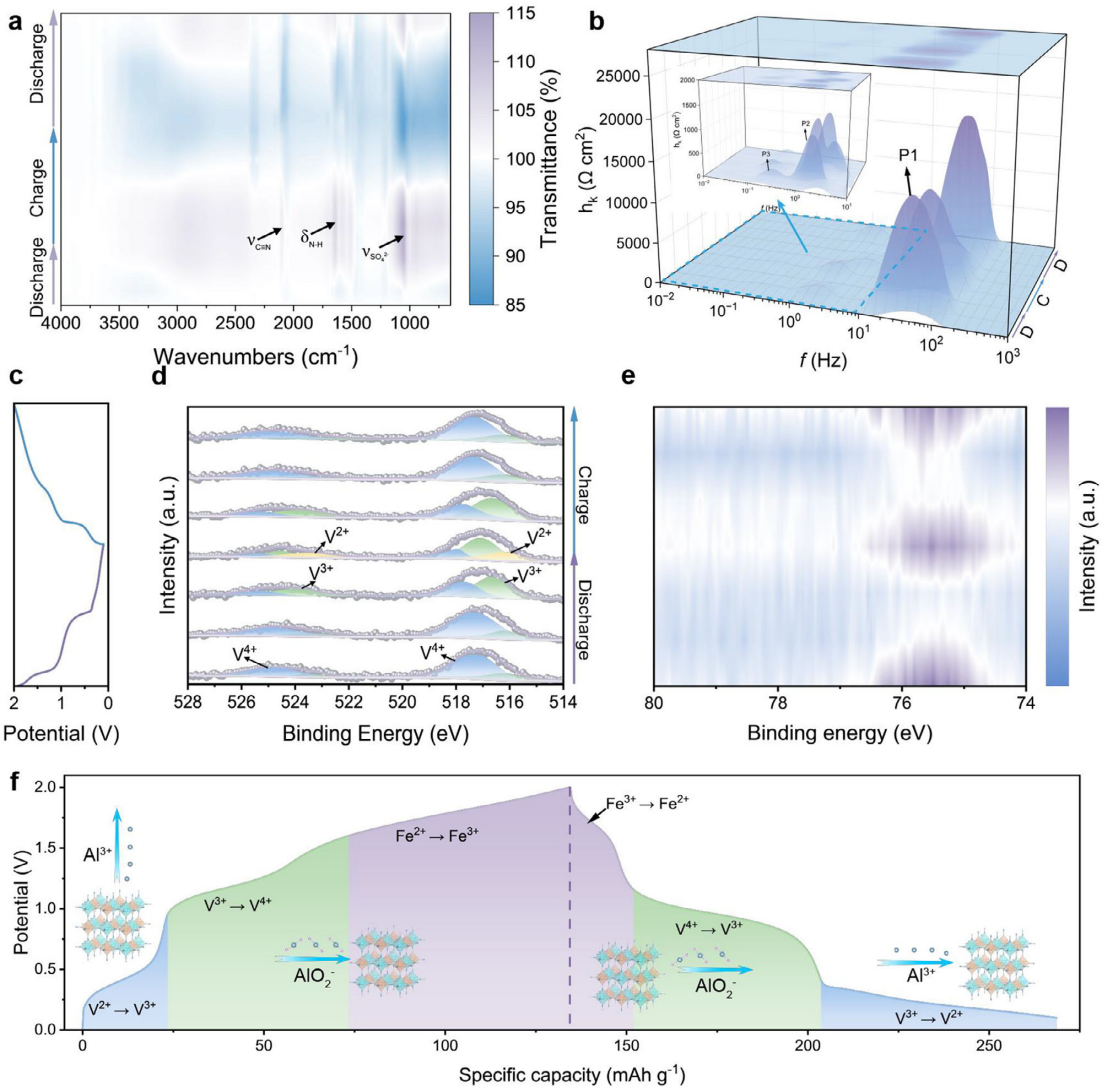
a) In‐situ FTIR spectra of VFePBA. b) In‐situ electrochemical impedance analysis with DRT (distribution of relaxation times) deconvolution of VFePBA. c) Charge/discharge curves of VFePBA. Ex situ XPS spectra of d)V 2p and e) Al 2p. f) Schematic illustration of the charge/discharge process.

The interfacial evolution of the Zn||AU15||VFePBA system during cycling was probed through in‐situ FTIR, revealing dynamic electrolyte‐electrode interactions (**Figure** **6**a). Spectral analysis demonstrates reversible intensity modulation of the ν(SO_4_^2−^) band at ≈1200 cm^−1^ and δ(N‐H) vibration at ≈1750 cm^−1^ during charge‐discharge cycles, attributed to electric field‐driven migration of ionic clusters (Al(SO_4_)_3_^3−^‐urea complexes) within the eutectic electrolyte. Notably, comparative analysis between the first discharge and subsequent cycles reveals attenuated intensities of these characteristic peaks after initial charging, suggesting partial consumption of SO_4_^2−^ anions and urea molecules through their participation in forming a cathode‐electrolyte interphase (CEI) layer on VFePBA surfaces during the initial activation process. This self‐limiting CEI layer, predominantly composed of sulfate‐ and nitrogen‐containing species, likely contributes to enhanced interfacial stability by passivating reactive sites and suppressing continuous electrolyte decomposition, as evidenced by the stabilized peak intensities in subsequent cycles. As showing in Figure 6b, In‐situ EIS combined with DRT analysis identified three characteristic peaks (P1, P2, P3) in frequency domains of 10^−1^‐10^0^, 10^0^–10^1^, and 10^1^–10^2^ Hz, corresponding to the ion transport at the interfacial CEI (R_CEI_), charge transfer in electrodes (R_ct_), and solid‐state diffusion (W_diff_), respectively. During discharge (2.0–1.4 V, 1.4–0.7 V, 0.7–0.1 V), R_CEI_, R_ct_, and W_diff_ increased in the first two stages but decreased in the third, while the charging process (0.1–1.0 V, 1.0–1.5 V, 1.5–2.0 V) exhibited an inverse trend. Ex situ XPS analysis (Figure 6c–e; Figure S68, Supporting Information) revealed synchronized evolution of Al 2p signals with intensity decreased during discharge phases I and II, then recovered in phase III, showing an inverse correlating with impedance variations. DFT computational analysis demonstrates that the high charge density of Al^3+^ facilitates binding with oxygen's lone‐pair electrons, promoting formation of Al‐O complexes (e.g., AlO^+^/AlO_2_^−^). This phenomenon was ascribed to AlO_2_^−^ anion migration from the cathode during early discharge stages (reducing Al content and increasing impedance) followed by Al^3+^ cation intercalation in phase III (enhancing conductivity). Vanadium oxidation state evolution during cycling displayed stage‐dependent characteristics, including minimal changes in V oxidation state during discharge phase I, reduction from V^4+^ to V^3+^ in phase II, and further reduction to V^2+^ in phase III, with reversed oxidation processes during charging. This behavior aligned with CV redox peaks, confirming V^4+^/V^3+^ and V^3+^/V^2+^ redox couples at low potentials and Fe^3+^/Fe^2+^ at high potentials.^[^ ^30^, ^33^, ^47^ ^]^ The proposed charge storage mechanism (Figure 6f) was further supported by in‐situ XRD results showing negligible peak shifts for VFePBA during cycling in AU15 electrolyte (Figure S69, Supporting Information), demonstrating enhanced structural stability and cycling performance. Figure S70 (v) presents the results of in‐situ UV–vis spectroscopy monitoring of the electrolyte during an 800 min cycling period. Throughout the cycle, no new absorption peaks appeared, confirming the absence of dissolved vanadium species or structural fragments. VFePBA effectively suppresses vanadium dissolution, offering a new approach to enhance the stability of vanadium‐based materials.

## 3 Conclusion

In summary, An acid‐assisted synthesis method with ligand modulation has been developed for the universal preparation V‐PBAs as high‐performance cathode materials for AAIBs. Electrochemically, the synergistic effect between the double active sites of Fe and V can enhances the entire redox activity, significantly boosting the capacity of V‐PBAs cathodes. Coupled with an aqueous eutectic electrolyte that extends the operational voltage window by 0.8 V, the optimized VFePBA cathode achieves an ultra‐high specific capacity of 161.37 mAh g^−1^ at 0.1 A g^−1^. The capacitance‐dominated charge storage mechanism facilitates the rapid Al^3+^ transport kinetics, endowing the battery with superior rate capability (78.4% capacity retention at 0.5 A g^−1^). In‐situ XRD analyses reveal minimal lattice parameter fluctuations during cycling in the eutectic system, demonstrating exceptional structural stability. Comprehensive characterization through XPS, CV, and EIS analyses confirms the multi‐electron redox processes involving V^2+^/V^3+^, V^3+^/V^4+^ and Fe^2+^/Fe^3+^ couples. Flexible pouch‐cell prototypes exhibit stable operation under mechanical deformation (180° bending) and successfully power electronic devices, validating the practical viability of V‐PBAs cathodes for next‐generation safe, high‐energy AAIBs.

## Conflict of Interest

The authors declare no conflict of interest.

## Supporting information



Supporting Information

Supporting Information

## Data Availability

The data that support the findings of this study are available from the corresponding author upon reasonable request.
