# Supplementary material for: High Performance Aluminum Ion Batteries Enabled by the Coordination Between Vanadium‐Based PBAs Cathode and Aqueous Eutectic Electrolyte
Source: Adv Sci (Weinh). 2025 Aug 13;12(42):e11274. doi: 10.1002/advs.202511274 (PMC12622544; doi:10.1002/advs.202511274)
Supplement: Supplementary file 1 — Supporting Information [file ADVS-12-e11274-s001.docx]

**Supporting Information**

**High performance aluminum ion batteries enabled by the coordination between Vanadium-based PBAs cathode and aqueous eutectic electrolyte**

*Wanchang Feng, Boman Li, Guoqiang Yuan, Yawei Li**, Yanfei Zhang, Meng Du, Yichun Su, Yijian Tang, Haotian Yue, Yuxin Li, Mohsen* *Shakouri, Hsiao-Chien Chen, Wenting Li, Zheng Liu,* and Huan Pang**

W. Feng, B. Li, G Yuan, Y. Li, Y. Zhang, M. Du, Y. Su, Y. Tang, W. Li, Z. Liu, H. Pang

School of Chemistry and Chemical Engineering, Yangzhou University, Yangzhou, 225002 Jiangsu, P. R. China.

H. Yue

School of Chemistry and Chemical Engineering, Chongqing University of Science & Technology, Chongqing 401331, China.

Y. Li

School of Environmental Science, Nanjing Xiaozhuang University, Nanjing, China.

M. Shakouri

Canadian Light Source Inc., University of Saskatchewan, Saskatoon, S7N 2V3, Canada.

H. Chen

Center for Reliability Science and Technologies, Chang Gung University, Kidney Research Center, Department of Nephrology, Chang Gung Memorial Hospital, Linkou, Taoyuan 333, Taiwan, China.

Z. Liu

Key Laboratory of Advanced Energy Materials Chemistry (Ministry of Education), Nankai University, Tianjin 300071, China

Corresponding author.

* Corresponding author.

*E-mail addresses*: liuzhengbeyond@163.com (Z. Liu), panghuan@yzu.edu.cn, huanpangchem@hotmail.com (H. Pang)

**Content**

[1. Experimental section 1](#_Toc205578217)

[1.1 Chem reagent 1](#_Toc205578218)

[1.2 Instrument require for experiment 1](#_Toc205578219)

[1.3 Materials synthesis 1](#_Toc205578220)

[1.4 Preparation of AU15 Eutectic Electrolyte 3](#_Toc205578221)

[1.5 Preparation of electrodes. 4](#_Toc205578222)

[1.6 Electrochemical measurements 4](#_Toc205578223)

[1.7 Computation methods 4](#_Toc205578224)

[1.8 In-situ XRD Experiment 6](#_Toc205578225)

[1.9 In-situ FTIR Experiment 6](#_Toc205578226)

[2. Schematic of coordination process 7](#_Toc205578227)

[3. SEM images of VFePBA at different reaction times. 8](#_Toc205578228)

[4. SEM images of VFeCoPBA with different Fe/Co ratios 9](#_Toc205578229)

[5. SEM images of VFeNiPBA with different Fe/Ni ratios 10](#_Toc205578230)

[6. SEM images of VCoNiPBA with different Co/Ni ratios 11](#_Toc205578231)

[7. SEM, HRTEM, dark-field images and elemental mapping of VFePBA-25^o^C 12](#_Toc205578232)

[8. XRD patterns of VFeCoPBA with different Fe/Co ratios 13](#_Toc205578233)

[9. XRD patterns of VFeNiPBA with different Fe/Ni ratios 14](#_Toc205578234)

[10. XRD patterns of VCoNiPBA with different Co/Ni ratios 15](#_Toc205578235)

[11. FTIR spectra of VFeCoPBA with different Fe/Co ratios 16](#_Toc205578236)

[12. FTIR spectra of VFeNiPBA with different Fe/Ni ratios 17](#_Toc205578237)

[13. FTIR spectra of VCoNiPBA with different Co/Ni ratios 18](#_Toc205578238)

[14. Raman spectra of VFeCoPBA with different Fe/Co ratios 19](#_Toc205578239)

[15. Raman spectra of VFeNiPBA with different Fe/Ni ratios 20](#_Toc205578240)

[16. Raman spectra of VCoNiPBA with different Co/Ni ratios 21](#_Toc205578241)

[17. XPS survey spectra 22](#_Toc205578242)

[18. Fe 2p XPS spectrum of VFePBA-25^o^C. 23](#_Toc205578243)

[19. Fe 2p XPS spectrum of VFeCoPBA 24](#_Toc205578244)

[20. Fe 2p XPS spectrum of VFeNiPBA 25](#_Toc205578245)

[21. Co 2p XPS spectrum of VFeCoPBA 26](#_Toc205578246)

[22. Co 2p XPS spectrum of VCoNiPBA 27](#_Toc205578247)

[23. Ni 2p XPS spectrum of VFeNiPBA 28](#_Toc205578248)

[24. Ni 2p XPS spectrum of VCoNiPBA 29](#_Toc205578249)

[25. V 2p XPS spectrum of VFePBA-25^o^C. 30](#_Toc205578250)

[26. BET surface area and pore size distribution 31](#_Toc205578251)

[27. CV Profiles of VFeCoPBA with Varied Fe/Co Ratios 32](#_Toc205578252)

[28. CV Profiles of VFeNiPBA with Varied Fe/Co Ratios 33](#_Toc205578253)

[29. Galvanostatic Charge-Discharge Profiles of VFeCoPBA with Varied Fe/Co Ratios 34](#_Toc205578254)

[30. Galvanostatic Charge-Discharge Profiles of VFeNiPBA with Varied Fe/Co Ratios 35](#_Toc205578255)

[31. CV curves, b values, and Capacitive Contribution Ratio of VFePBA 36](#_Toc205578256)

[32. Capacitive Contribution Ratio at different scan rates of VFePBA 37](#_Toc205578257)

[33. CV curves, b values, and Capacitive Contribution Ratio of VFeCoPBA-8:2 38](#_Toc205578258)

[34. Capacitive Contribution Ratio at different scan rates of VFeCoPBA-8:2 39](#_Toc205578259)

[35. CV curves, b values, and Capacitive Contribution Ratio of VFeCoPBA-6:4 40](#_Toc205578260)

[36. Capacitive Contribution Ratio at different scan rates of VFeCoPBA-6:4 41](#_Toc205578261)

[37. CV curves, b values, and Capacitive Contribution Ratio of VFeCoPBA-4:6 42](#_Toc205578262)

[38. Capacitive Contribution Ratio at different scan rates of VFeCoPBA-4:6 43](#_Toc205578263)

[39. CV curves, b values, and Capacitive Contribution Ratio of VFeCoPBA-2:8 44](#_Toc205578264)

[40. Capacitive Contribution Ratio at different scan rates of VFeCoPBA-2:8 45](#_Toc205578265)

[41. CV curves, b values, and Capacitive Contribution Ratio of VFeNiPBA-8:2 46](#_Toc205578266)

[42. Capacitive Contribution Ratio at different scan rates of VFeNiPBA-8:2 47](#_Toc205578267)

[43. CV curves, b values, and Capacitive Contribution Ratio of VFeNiPBA-6:4 48](#_Toc205578268)

[44. Capacitive Contribution Ratio at different scan rates of VFeNiPBA-6:4 49](#_Toc205578269)

[45. CV curves, b values, and Capacitive Contribution Ratio of VFeNiPBA-4:6 50](#_Toc205578270)

[46. Capacitive Contribution Ratio at different scan rates of VFeNiPBA-4:6 51](#_Toc205578271)

[47. CV curves, b values, and Capacitive Contribution Ratio of VFeNiPBA-2:8 52](#_Toc205578272)

[48. Capacitive Contribution Ratio at different scan rates of VFeNiPBA-2:8 53](#_Toc205578273)

[49. Rate performance of VFeNiPBA-8:2 54](#_Toc205578274)

[50. Rate performance of VFeNiPBA-6:4 55](#_Toc205578275)

[51. Rate performance of VFeNiPBA-4:6 56](#_Toc205578276)

[52. CV curves, b values, and Capacitive Contribution Ratio of VFePBA-25^o^C 57](#_Toc205578277)

[53. Capacitive Contribution Ratio at different scan rates of VFePBA-25^o^C 58](#_Toc205578278)

[54. Cycling performance of VFePBA 59](#_Toc205578279)

[55. Charge-discharge profiles of VFePBA 60](#_Toc205578280)

[56. Cycling performance of VFePBA-25oC 61](#_Toc205578281)

[57. In-situ EIS results of VFePBA 62](#_Toc205578282)

[58. Cycling performance in different aqueous electrolytes of VFePBA 63](#_Toc205578283)

[59. Cycling performance of VFePBA in Al||1 M Al(OTF)_3_ + 0.2 M Mn(OTF)_2_||VFePBA 64](#_Toc205578284)

[60. Cycling performance of VFePBA in Zn||1 M Al(OTF)_3_ + 0.2 M Mn(OTF)_2_||VFePBA 65](#_Toc205578285)

[61. CV curves, b values, and Capacitive Contribution Ratio of Zn||AU15||VFePBA 66](#_Toc205578286)

[62. Capacitive Contribution Ratio at different scan rates of Zn||AU15||VFePBA 67](#_Toc205578287)

[63. EIS and Equivalent Circuit Diagram of the Zn||AU15||VFePBA System 68](#_Toc205578288)

[64. Contact angle between VFePBA and electrolyte 69](#_Toc205578289)

[65. Charge-discharge profiles of Pouch cell demonstration of VFePBA 70](#_Toc205578290)

[66. Structure of VFePBA before Al^3+^ intercalation 71](#_Toc205578291)

[67. Charge distribution on the (101) crystal plane of VFePBA 72](#_Toc205578292)

[68. Structure of VFePBA after Al^3+^ intercalation 73](#_Toc205578293)

[69. Ex situ XPS spectra of VFePBA: Zn 2p 74](#_Toc205578294)

[70. In-situ XRD patterns of VFePBA in Zn||AU15||VFePBA 75](#_Toc205578295)

[71. In-situ UV spectroscopy of VFePBA in Zn||AU15||VFePBA 76](#_Toc205578296)

[Rerfence 77](#_Toc205578297)

# 1. Experimental section

## 1.1 Chem reagent

All reagents were of analytical grade and could be used without further purification. Ammonium metavanadate (NH_4_VO_3_, 99%), Vanadium oxide sulfate hydrate (VOSO_4_·xH_2_O, 97%), Oxalic acid dihydrate (H_2_C_2_O_4_, 99.5%), Potassium ferricyanide (K_3_Fe(CN)_6_, 99.5%), Potassium hexacyanocobaltate (K_3_Co(CN)_6_, 99%), Potassium tetracyanonickelate (K_2_Ni(CN)_4_, 99%), Aluminum nitrate nonahydrate (Al(NO_3_)_3_·9H_2_O, 99%), Aluminum sulfate octadecahydrate (Al_2_(SO_4_)_3_·18H_2_O, 99%), Aluminum chloride hexahydrate (AICI_3_·6H_2_O, 99%), Urea (H_2_NCONH_2_, 99%).

## 1.2 Instrument require for experiment

Electronic analytical balance (GL224-1SCN), purchased from Sartorius Scientific Instruments (Beijing) Co., Ltd. The phase and crystal structure of the material were characterized by X-ray diffraction (XRD) on a Bruker D8 Advanced X-ray Diffractometer (Cu-Kα radiation: λ = 0.15406 nm). The morphology of samples was observed by scanning electron microscope (SEM, Zeiss_Supra55) under the acceleration voltage of 5.0 kV. Transmission electron microscopy (TEM) investigations were performed by a JEM-2100 instrument. Energy dispersive X-ray spectrometry (EDX) elemental mapping scans were recorded using Tecnai G2 F30 S-TWIN at an acceleration voltage of 300 kV. Thermogravimetric measurements were determined via a PerkinElmer Pyris 1 TGA thermogravimetric analysis (TGA) instrument. The chemical states were measured using an Axis Ultra X-ray photoelectron spectroscope (XPS, Thermo Fisher Scientific ESCALAB250Xi) equipped with a standard monochromatic Al-Kα source (hv = 1486.6 eV). Fourier transform infrared (FTIR) transmission spectra were obtained on a BRUKER-EQUINOX-55 IR spectrophotometer. The products were tested by Raman on a DXRxi Raman Imaging Microscope for functional group analysis. Autosorb-Iq obtained the N_2_ sorption isothermals and pore size distribution via Brunauer-Emmet-Teller (BET) method. Electrochemical measurements were conducted on a CHI-660E electrochemical workstation (CH Instruments). Electrochemical impedance spectroscopy (EIS) measurements were performed using a DH7000D system (Donghua Testing Technology Co., Ltd.). Battery charge-discharge testing was conducted using a LAND battery testing system (Wuhan LAND Electronics Co., Ltd.).

## 1.3 Materials synthesis

**Synthesis of VFePBA.**

The synthesis procedure of VFePBA was adapted from the literature^[1]^.

In a beaker containing 50 mL deionized water, 2.57 mmol NH_4_VO_3_, 6.43 mmol oxalic acid, and 1.71 mmol K_3_[Fe(CN)_6_] were sequentially added under continuous stirring at room temperature to form a homogeneous transparent solution. The mixture was transferred into a 100 mL Teflon-lined stainless steel autoclave and heated at 180°C for different reaction durations. After cooling to room temperature, the product was collected by centrifugation, washed three times with deionized water, and dried at 60°C for 24 hours to obtain the VFePBA powder.

**Synthesis of VCoPBA.**

The synthesis procedure of VCoPBA is as follows: In a beaker containing 50 mL deionized water, 2.57 mmol NH_4_VO_3_, 6.43 mmol oxalic acid, and 1.71 mmol K_3_[Fe(CN)_6_] were sequentially added under continuous stirring at room temperature to form a homogeneous transparent solution. The mixture was transferred into a 100 mL Teflon-lined stainless steel autoclave and heated at 160°C for 24 h. After cooling to room temperature, the product was collected by centrifugation, washed three times with deionized water, and dried at 60°C for 24 hours to obtain the VCoPBA powder.

**Synthesis of VNiPBA.**

The synthesis procedure of VNiPBA is as follows: In a beaker containing 50 mL deionized water, 3.6 mmol VOSO_4_·xH_2_O, 2.4 mmol K_2_Ni(CN)_4_, were sequentially added under continuous stirring at room temperature to form a homogeneous transparent solution. The mixed product was aged at room temperature for 24 h. After aging, the product was collected by centrifugation, washed three times with deionized water, and dried at 60°C for 24 hours to obtain the VNiPBA powder.

**Synthesis of VFeCoPBA.**

The modified synthesis procedure for VFeCoPBA with varying Fe/Co ratios is described as follows: In a beaker containing 50 mL deionized water, 2.57 mmol NH_4_VO_3_ and 6.43 mmol oxalic acid were dissolved under continuous stirring at room temperature. Subsequently, a total molar amount of 1.71 mmol cyanide precursors (specifically K_3_[Fe(CN)_6_] and K_3_[Co(CN)_6_] in molar ratios of 8:2, 6:4, 4:6 and 2:8) were added sequentially to form homogeneous transparent solutions. The mixtures were transferred into 100 mL Teflon-lined stainless steel autoclaves and hydrothermally treated at 160°C for 24 h. After cooling to room temperature, the products were collected by centrifugation, washed three times with deionized water, and dried at 60°C for 24 h to obtain VFeCoPBA powders with designated Fe/Co ratios (VFeCoPBA-8:2, VFeCoPBA-6:4, VFeCoPBA-4:6, VFeCoPBA-2:8).

**Synthesis of VFeNiPBA.**

The modified synthesis procedure for VFeNiPBA with varying Fe/Ni ratios is described as follows: In a beaker containing 50 mL deionized water, 2.57 mmol NH_4_VO_3_ and 6.43 mmol oxalic acid were dissolved under continuous stirring at room temperature. Subsequently, a total molar amount of 1.71 mmol cyanide precursors (specifically K_3_[Fe(CN)_6_] and K_2_[Ni(CN)_4_] in molar ratios of 8:2, 6:4, 4:6 and 2:8) were added sequentially to form homogeneous transparent solutions. The mixtures were transferred into 100 mL Teflon-lined stainless steel autoclaves and hydrothermally treated at 120°C for 24 h. After cooling to room temperature, the products were collected by centrifugation, washed three times with deionized water, and dried at 60°C for 24 h to obtain VFeNiPBA powders with designated Fe/Ni ratios (VFeNiPBA-8:2, VFeNiPBA-6:4, VFeNiPBA-4:6, VFeNiPBA-2:8).

**Synthesis of VCoNiPBA.**

The modified synthesis procedure for VCoNiPBA with varying Co/Ni ratios is described as follows: In a beaker containing 50 mL deionized water, 2.57 mmol NH_4_VO_3_ and 6.43 mmol oxalic acid were dissolved under continuous stirring at room temperature. Subsequently, a total molar amount of 1.71 mmol cyanide precursors (specifically K_3_[Co(CN)_6_] and K_2_[Ni(CN)_4_] in molar ratios of 8:2, 6:4, 4:6 and 2:8) were added sequentially to form homogeneous transparent solutions. The mixtures were transferred into 100 mL Teflon-lined stainless steel autoclaves and hydrothermally treated at 120°C for 24 h. After cooling to room temperature, the products were collected by centrifugation, washed three times with deionized water, and dried at 60°C for 24 h to obtain VCoNiPBA powders with designated Fe/Ni ratios (VCoNiPBA-8:2, VCoNiPBA-6:4, VCoNiPBA-4:6, VCoNiPBA-2:8).

## 1.4 Preparation of AU15 Eutectic Electrolyte

The Preparation of AU15 Eutectic Electrolyte was adapted from the literature ^[2]^.

The AU15 eutectic electrolyte was formulated with a molar ratio of Al_2_(SO_4_)_3_·18H_2_O, urea, and deionized water at 1:6:15. The synthesis procedure involved two sequential steps: (1) Al_2_(SO_4_)_3_·18H_2_O and urea were mixed under continuous stirring at 60 °C in air for 1 h to obtain a homogeneous liquid precursor. (2) Deionized water was then introduced into the AU solution, followed by additional stirring for 1 h at 60 °C using a water bath to ensure complete homogenization, yielding the final AU15 electrolyte.

## 1.5 Preparation of electrodes.

Electrodes for three-electrode system: Prior to the preparation, the carbon cloth (CC) was first ultrasonically cleaned in acetone, absolute ethanol and DI water alternately for 3 - 4 times for 30 min each time. Then, the treated CC was placed in a vacuum drying oven to be dried at 60 °C overnight. To fabricate the working electrodes, polyvinylidene fluoride (PVDF, 10 wt.%), Super P carbon (30 wt.%) and active materials (60 wt.%) were well mixed in N-methypyrrolidone (NMP) solvent. Then, the formed slurry was sonicated for 30 min before depositing on a 1 cm × 2 cm CC, ensuring samples coated within 1 cm × 1 cm. The typical mass loading of the active material was about 1.2 mg cm^-2^. Finally, Electrodes were acquired after drying overnight at 60 °C in a vacuum drying oven.

Electrodes for Zn||AU15||VFePBA: The electrochemical performances were measured in coin cells. The working electrodes were prepared by mixing polyvinylidene fluoride (PVDF, 10 wt.%), Super P carbon (30 wt.%) and active materials (60 wt.%) in N-methypyrrolidone (NMP) solvent on titanium foil which was used as the current collectors. The coated electrode was dried in vacuum at 60℃ for 12 h. The separator was glass fiber. AU15 as the electrolyte.

## 1.6 Electrochemical measurements

Electrochemical measurements were carried out on an electrochemical working station (CHI 660E, Shanghai Chenhua) in a three-electrode system, in which the V-PBAs electrode was used as the working electrode, the platinum electrode as a counter electrode and the saturated calomel (SCE) electrode as the reference electrode. The electrolyte was 1.0 M Al(NO_3_)_3_ solution. To compare the cation effects on Al insertion The V-PBAs electrodes were tested with cyclic voltammetry and galvanostatic charge-discharge methods. In the galvanostatic charge/discharge test, the potential ranged from 0 to 1.2 V (vs. SCE).

Full-cell measurements were performed on Zn||AU15||VFePBA. The cycling performance and rate capability were tested by a battery measurement system (CT3002A, Wuhan Land, China). The cyclic voltammetry (CV) were tested on a CHI 760E electrochemical workstation, and the CV curves was obtained in the potential range from 0.1 to 2.0 V. Electrochemical impedance spectroscopy (EIS) were tested on a DH7000D electrochemical workstation

All the electrochemical measurements were performed at ambient temperature.

## 1.7 Computation methods

All calculations were implemented in Materials Studio with the CASTEP code. The Perdew-Burke-Ernzerhof (PBE) functional of the generalized gradient approximation (GGA) was used to calculate the exchange-correlation energy. ^[3, 4]^ The ultrasoft pseudopotential for every atom was adopted. The cutoff energy was 500 eV, the Γ-centered k-mesh was adopted as 2×2×1 for the geometry optimization of all structures. The convergence tolerances of energy change, maximum force, and maximum displacement were set as 2×10^-6^ eV/atom, 0.05 eV/Å, and 0.002 Å, respectively. The complete linear synchronous transit (LST) and quadratic synchronous transit (QST) methods were employed to search the transition state^[5, 6]^.

The ion diffusion coefficients (D) were estimated through Galvanostatic Intermittent Titration Technique (GITT) and calculated according to the following equation:

$D_{Zn}=\frac{4}{\pi\tau}\left( \frac{n_{m}V_{m}}{S} \right)^{2}\left( \frac{\Delta E_{s}}{\Delta E_{t}} \right)^{2}$ (1)

Here, *τ* is the duration of the current pulse (s); *n_m_* is the number of moles (mol); *V_m_* is the molar volume of the electrode (cm^3^·mol^-1^); *S* is the electrode/electrolyte contact area (cm^2^); Δ*E_s_* is the steady-state voltage change, due to the current pulse and Δ*E_t_* is the voltage change during the constant current pulse, eliminating the iR drop.

The kinetic of capacitive contribution can be obtained through calculating the CV curves at different scan rates. The relationship between current (i) and scan rate (v) can be written as:

$i=av^{b}$ (2)

$\log\left( i \right)=b\cdot\log\left( v \right)+\log\left( a \right)$ (3)

where *i* is the current (A), *v* is the scan rate (mV·s^-1^), and *a* and *b* are adjustable parameters. The value of *b* is from 0.5 to 1, wherein *b*=0.5 indicates a full diffusion controlled process and *b*=1 corresponds to the full capacitive contribution.

$ⅈ\left( V \right)=k_{1}v+k_{2}v^{1/2}$ (4)

where in *k_1_* and *k_2_* are variable parameters. When the voltage is fixed, the response current *i (V)* consists of surface capacitive (*k_1_v*) and diffusion-controlled (*k_2_v^1/2^*) contribution. The following equation can be obtained through equivalent transformation:

$i\left( V \right)/v^{1/2}=k_{1}v^{1/2}+k_{2}$ (5)

There is a linear relationship between *i/v^1/2^* and *v^1/2^*. Thus, *k_1_* and *k_2_* can be concluded by linear fitting. Thereby, the ratio of the capacitive and diffusion-controlled contribution can be obtained.

## 1.8 In-situ XRD Experiment

Measurements were conducted using a MiniFlex-600X diffractometer (Rigaku) with Cu Kα radiation (λ = 1.5406 Å). Electrodes were cycled at a constant current density of 100 mA g^-1^ within electrolyte-specific voltage windows: 0-1.2 V vs. Al^3+^/Al for aqueous electrolyte and 0.1-2.0 V vs. Al^3+^/Al for AU15 electrolyte. XRD patterns were acquired across the 5°-45° (2θ) range with a step size of 0.02° and counting time of 2 s per step, capturing data at critical electrochemical states including open-circuit voltage, charge/discharge plateaus, and cycle endpoints.

## 1.9 In-situ FTIR Experiment

The in-situ FTIR measurements are performed at room temperature using a PerkinElmer Spectrum 3 equipped with a MCT (mercury-cadmium-telluride) detector cooled by liquid nitrogen. The test spectrum has a scanning range of 4000 cm^-1^ to 650 cm^-1^, with a resolution of 4 cm^-1^. In-situ tests were conducted utilizing the electrochemical cell mould produced by Shanghai Yuanfang Technology Co. To monitor changes during charging and discharging, a zinc selenide half-cylinder was employed as the reflector. During the test, the assembled in-situ cell mould (devoid of electrode material) was scanned as a background, and the initial spectrum (devoid of current) was collected. Following a sufficient period of rest for the cell, the spectra during the charging and discharging processes were collected.

# 2. Schematic of coordination process


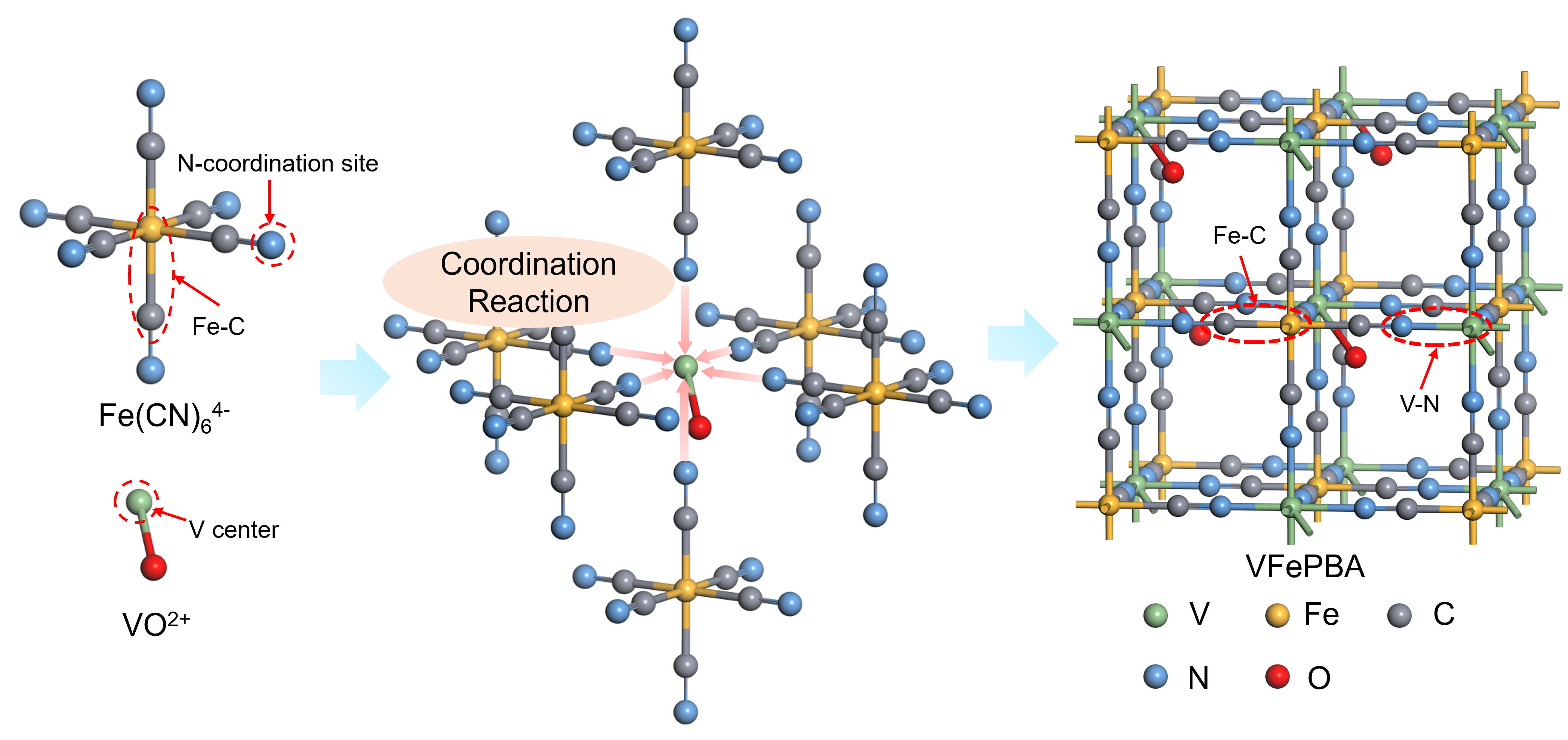


**Figure S1. Schematic of coordination process**

# 3. SEM images of VFePBA at different reaction times.


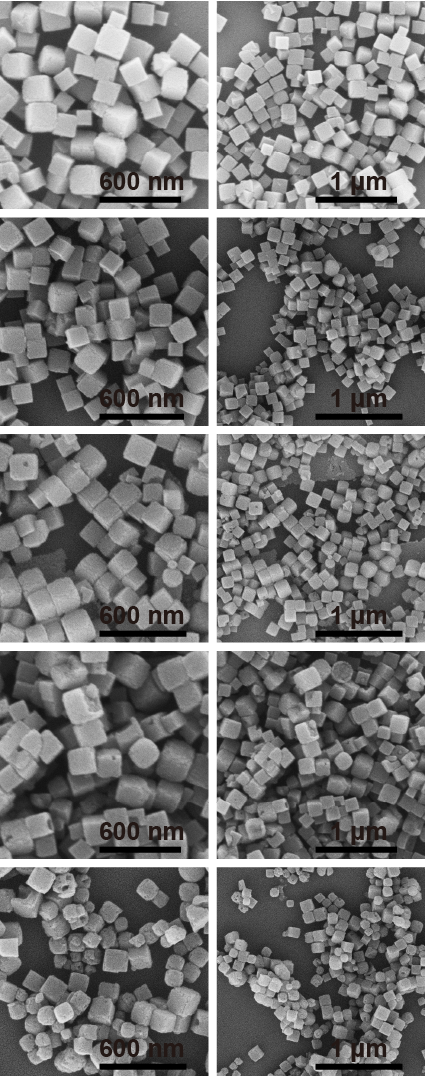


**Figure S2.** SEM images of VFePBA at different reaction times.

# 4. SEM images of VFeCoPBA with different Fe/Co ratios


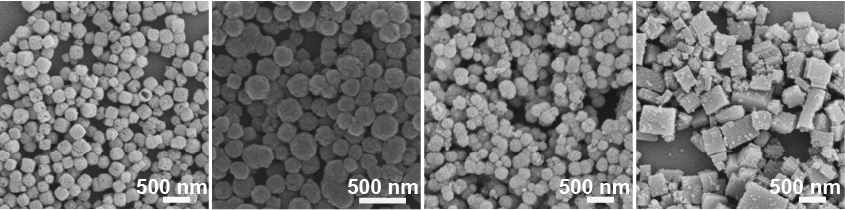


**Figure S3.** SEM images of VFeCoPBA with different Fe/Co ratios , from left to right: 8:2, 6:4, 4:6, 2:8.

# 5. SEM images of VFeNiPBA with different Fe/Ni ratios


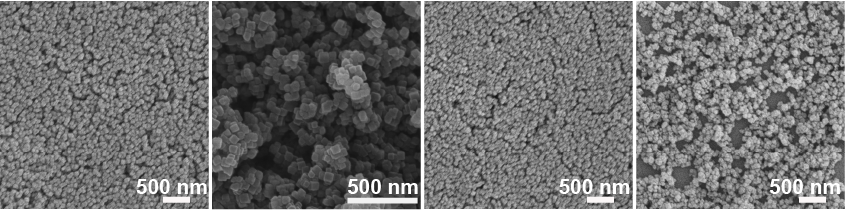


**Figure S4.** SEM images of VFeNiPBA with different Fe/Ni ratios from left to right: 8:2, 6:4, 4:6, 2:8.

# 6. SEM images of VCoNiPBA with different Co/Ni ratios


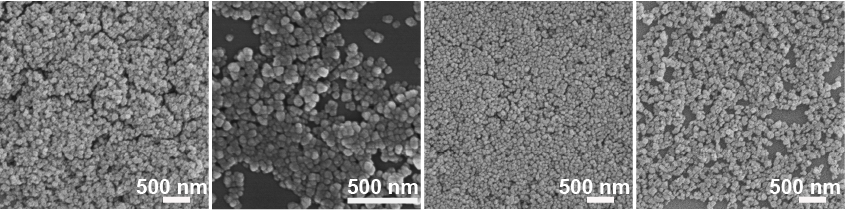


**Figure S5.** SEM images of VCoNiPBA with different Co/Ni ratios from left to right: 8:2, 6:4, 4:6, 2:8.

# 7. SEM, HRTEM, dark-field images and elemental mapping of VFePBA-25^o^C


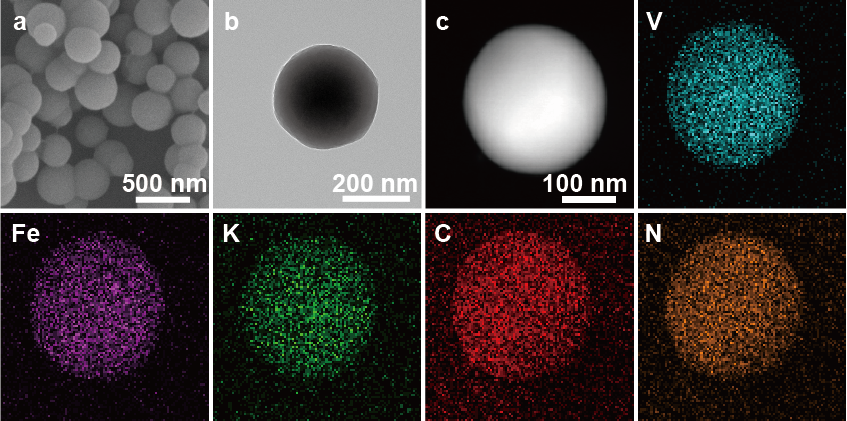


**Figure S6.** SEM, HRTEM, dark-field images and elemental mapping of VFePBA-25^o^C.

# 8. XRD patterns of VFeCoPBA with different Fe/Co ratios


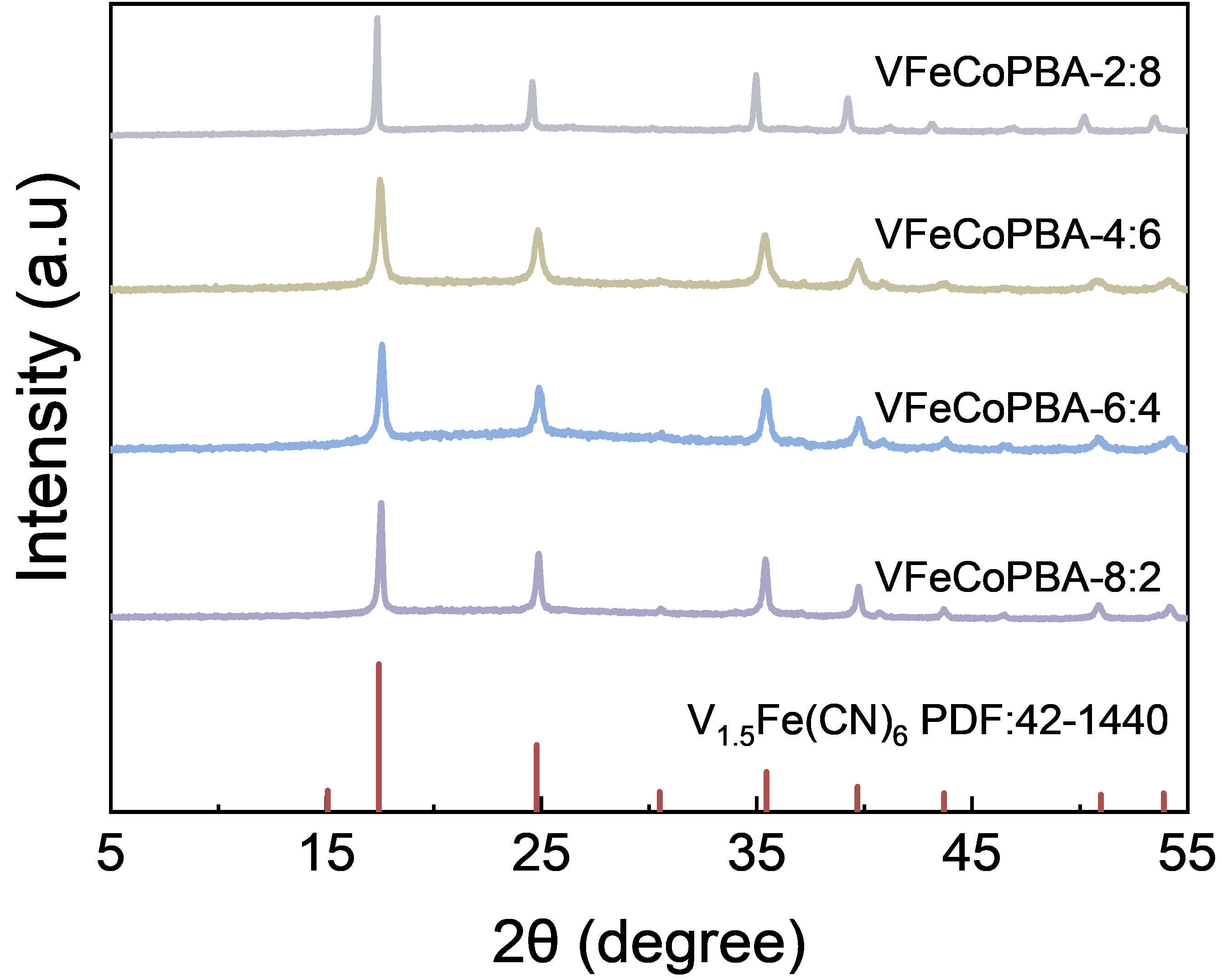


**Figure S7.** XRD patterns of VFeCoPBA with different Fe/Co ratios.

# 9. XRD patterns of VFeNiPBA with different Fe/Ni ratios


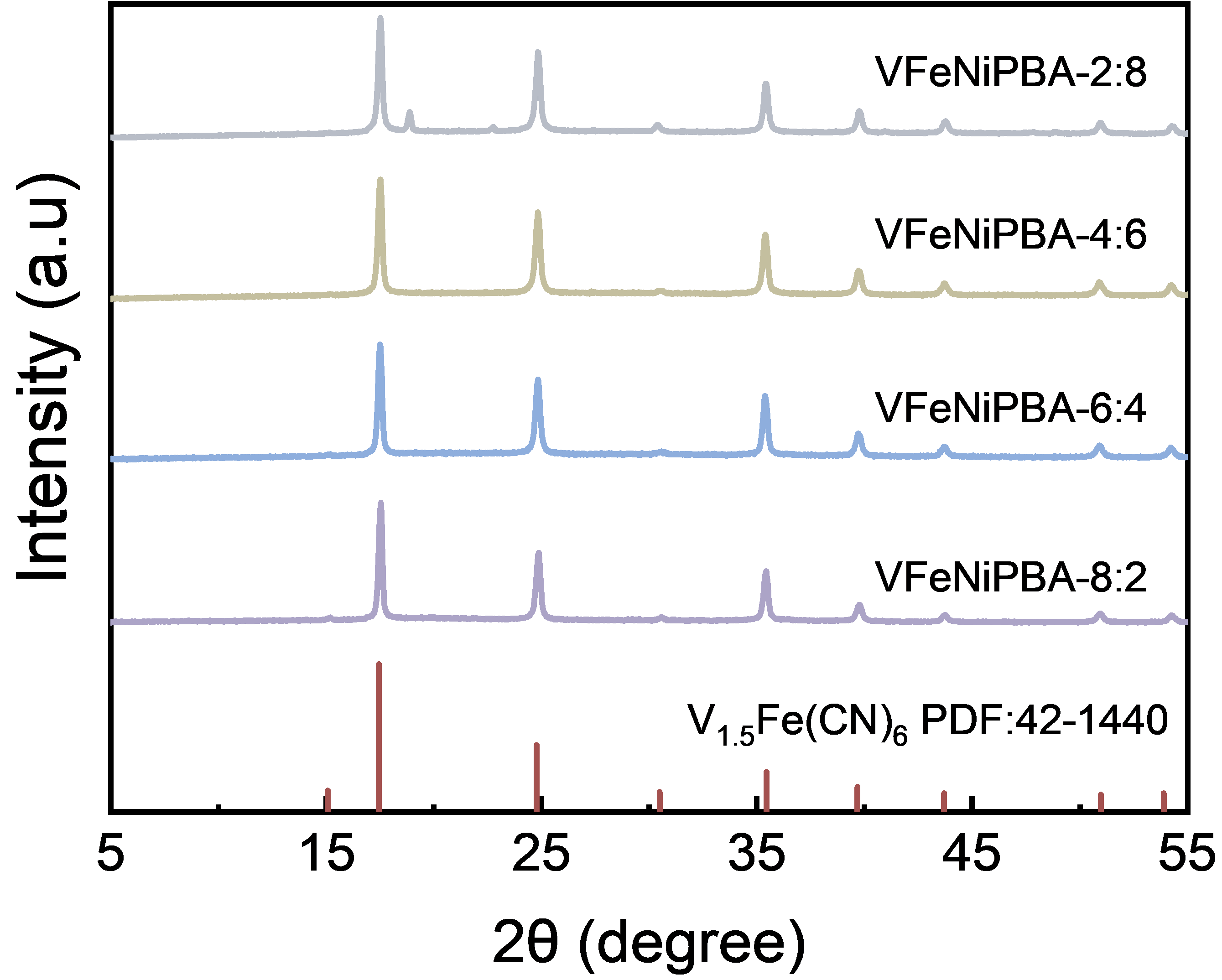


**Figure S8.** XRD patterns of VFeNiPBA with different Fe/Ni ratios.

# 10. XRD patterns of VCoNiPBA with different Co/Ni ratios


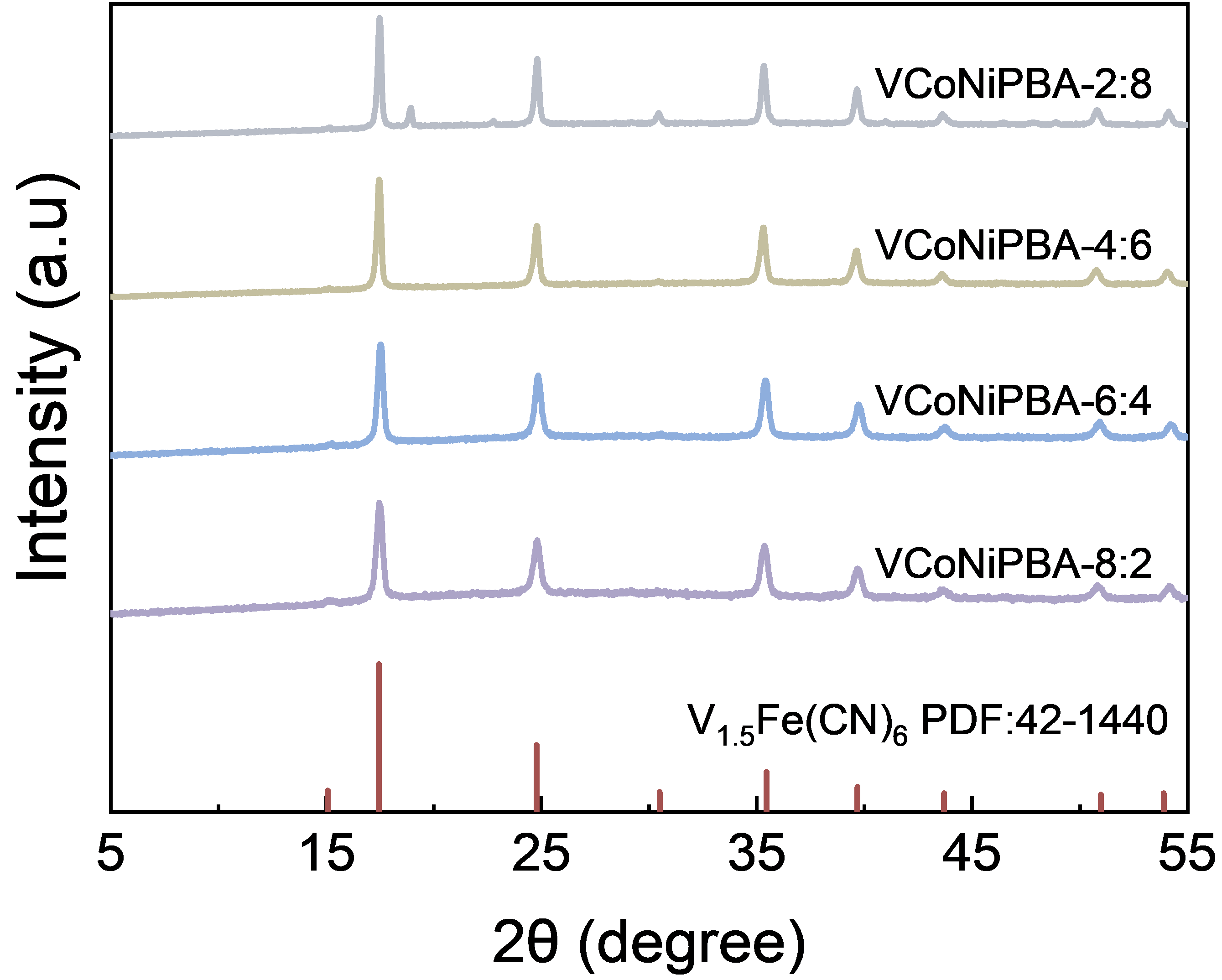


**Figure S9.** XRD patterns of VCoNiPBA with different Co/Ni ratios.

# 11. FTIR spectra of VFeCoPBA with different Fe/Co ratios


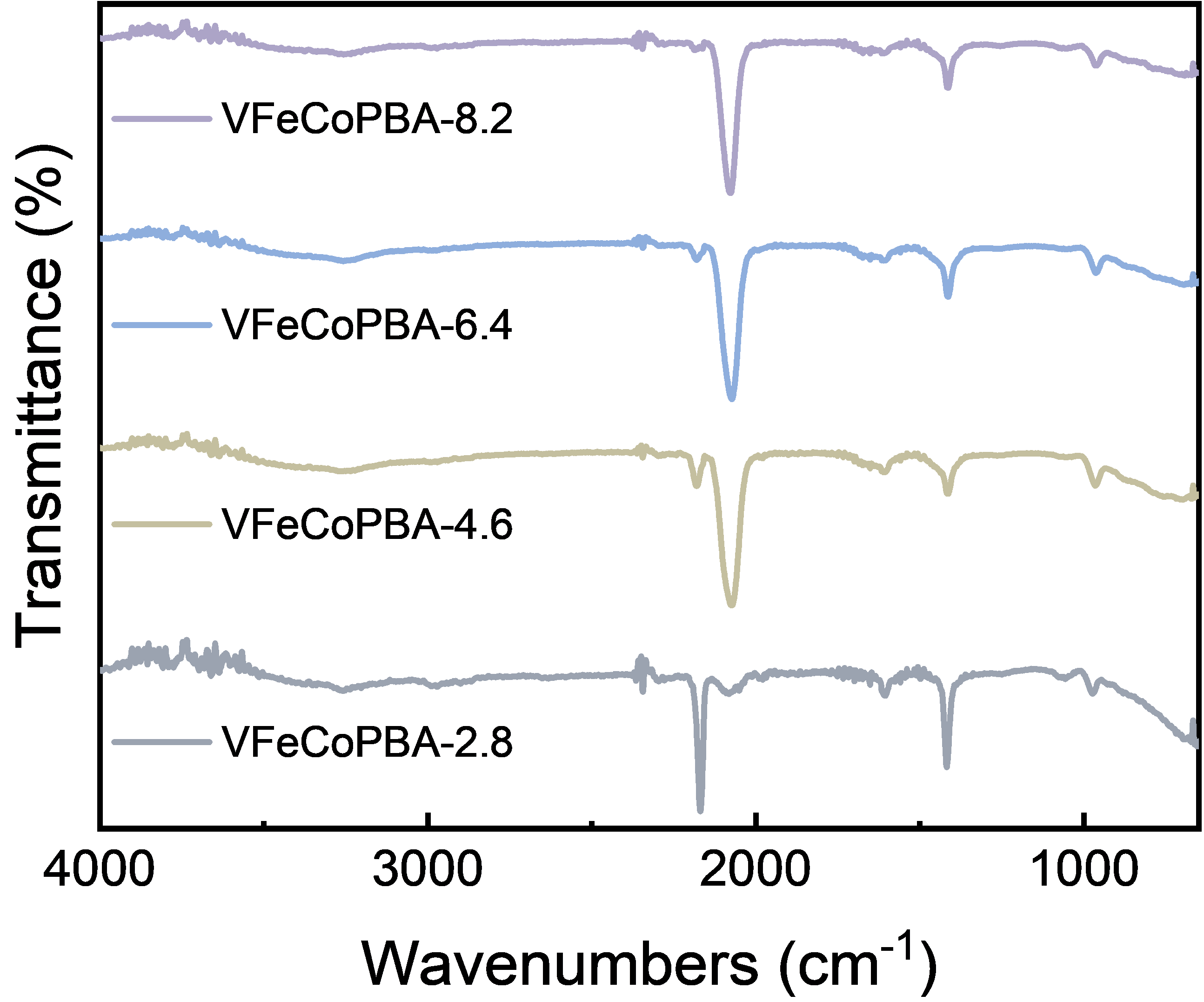


**Figure S10.** FTIR spectra of VFeCoPBA with different Fe/Co ratios.

# 12. FTIR spectra of VFeNiPBA with different Fe/Ni ratios


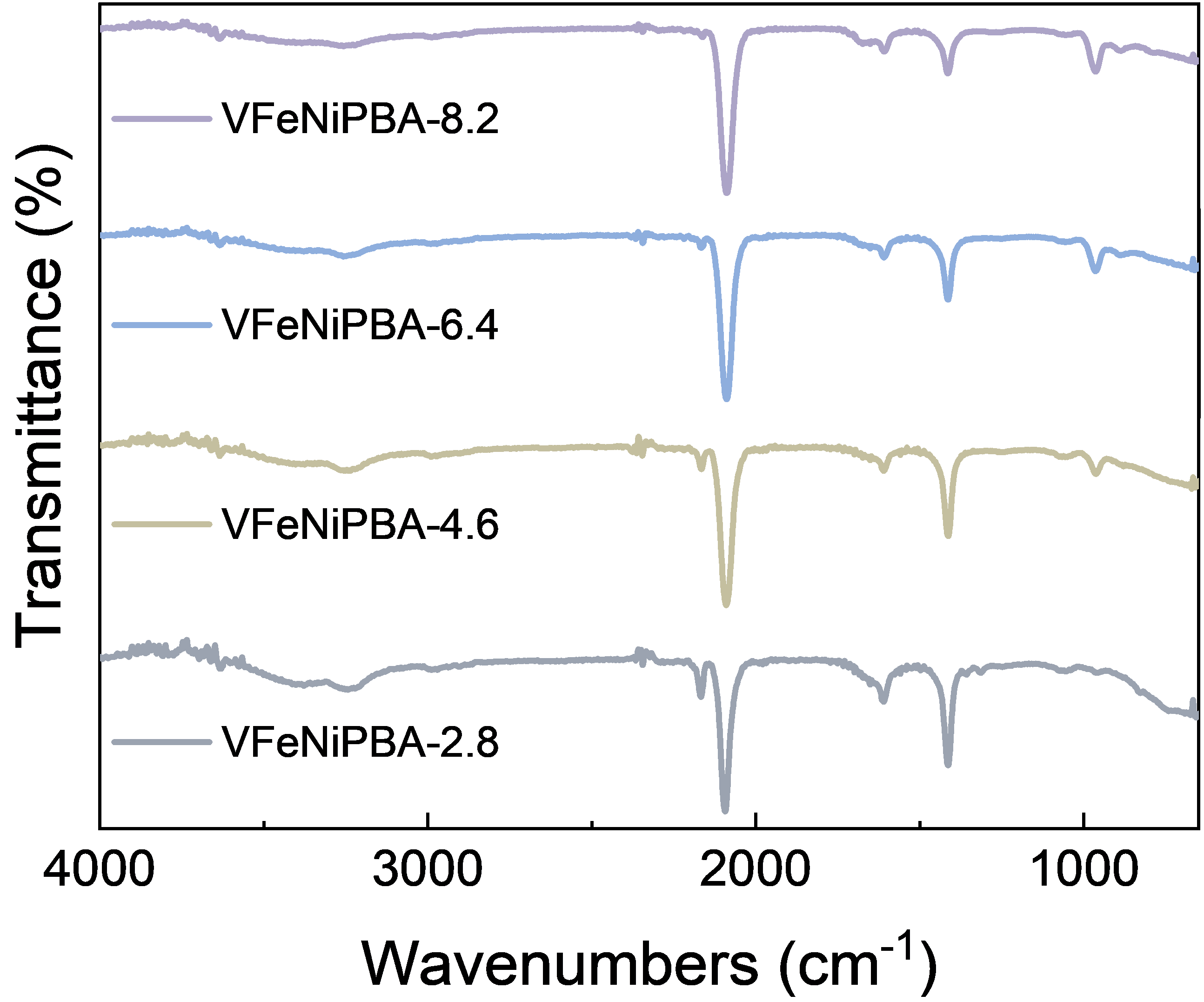


**Figure S11.** FTIR spectra of VFeNiPBA with different Fe/Ni ratios.

# 13. FTIR spectra of VCoNiPBA with different Co/Ni ratios


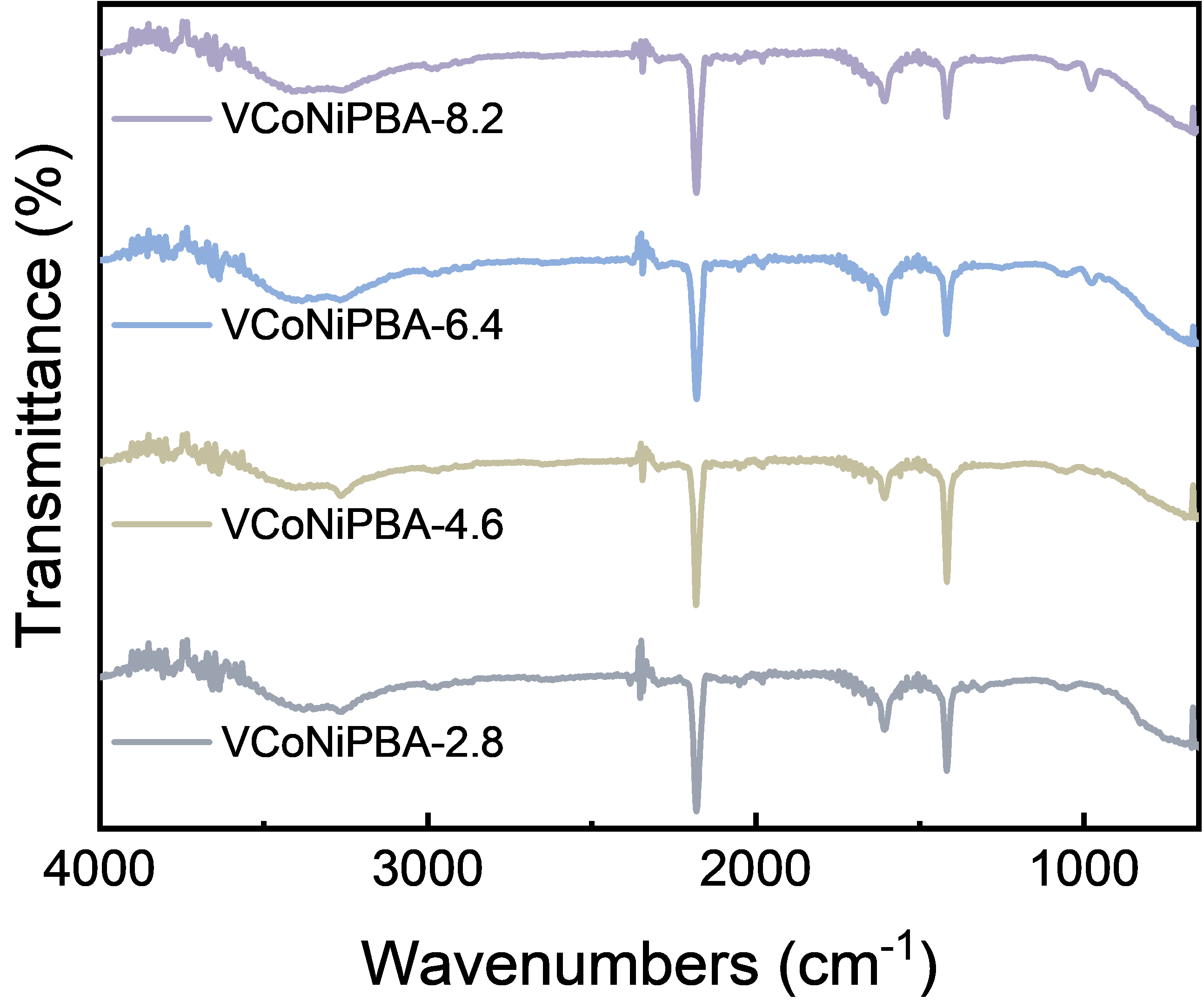


**Figure S12.** FTIR spectra of VCoNiPBA with different Co/Ni ratios.

# 14. Raman spectra of VFeCoPBA with different Fe/Co ratios


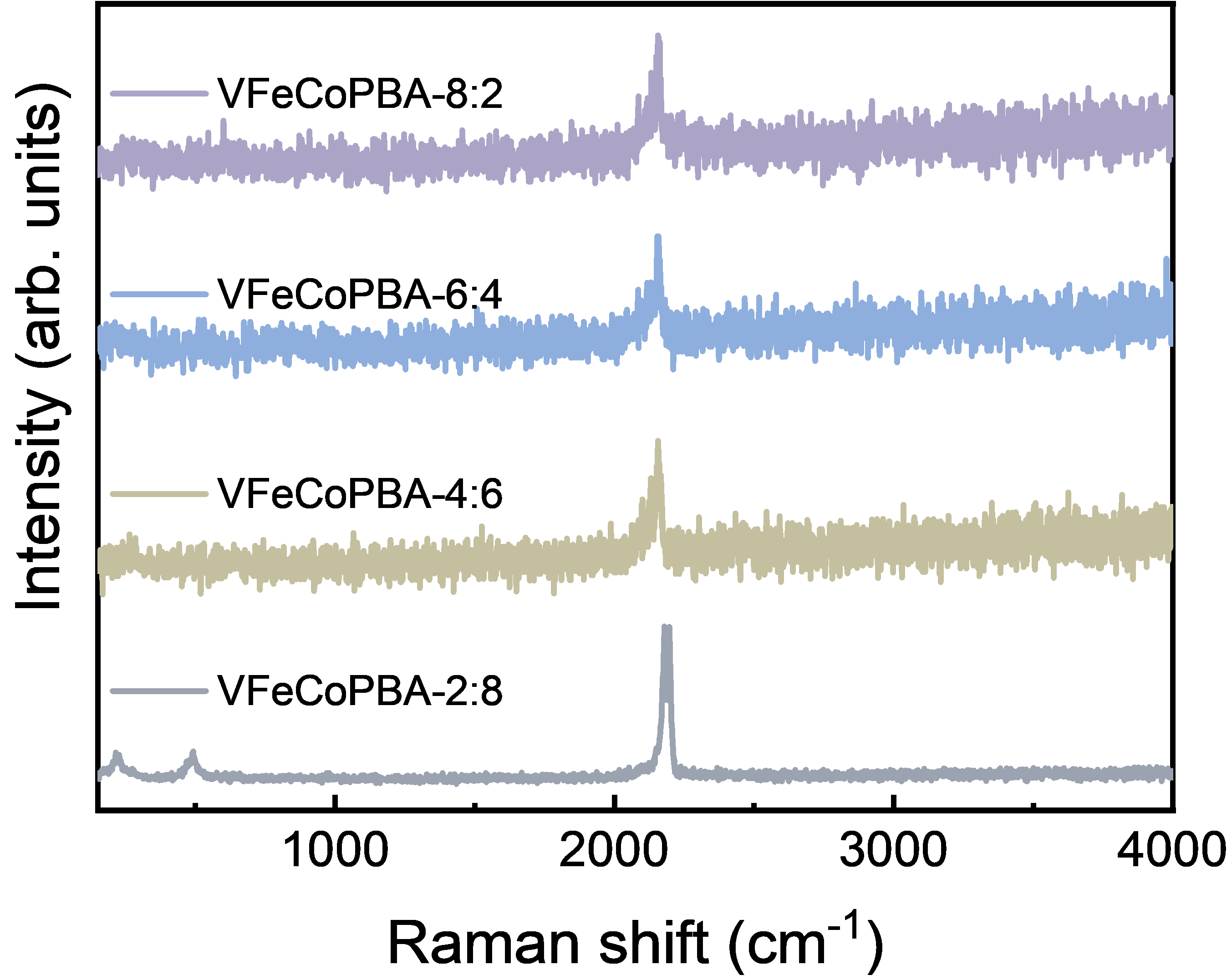


**Figure S13.** Raman spectra of VFeCoPBA with different Fe/Co ratios.

# 15. Raman spectra of VFeNiPBA with different Fe/Ni ratios


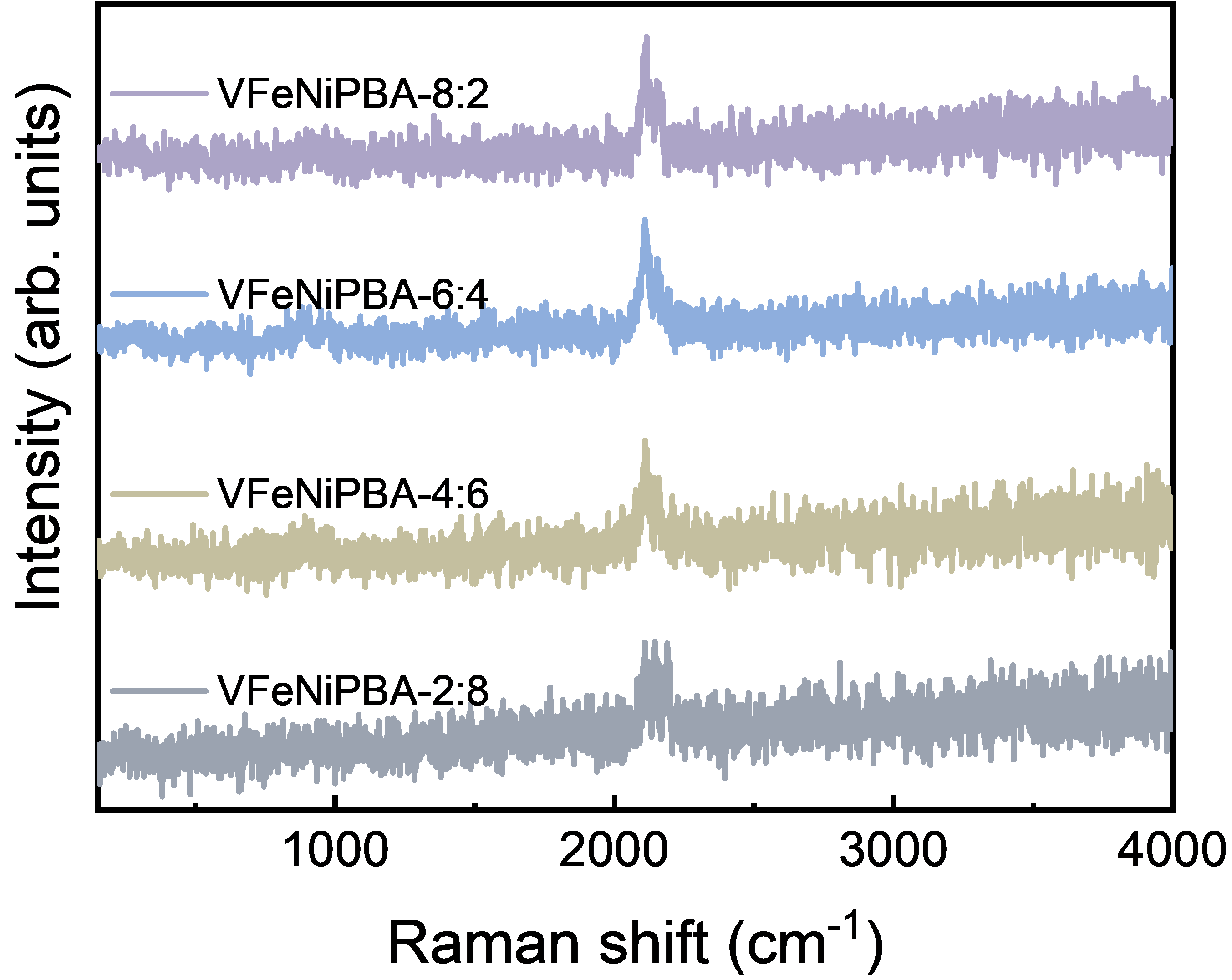


**Figure S14.** Raman spectra of VFeNiPBA with different Fe/Ni ratios.

# 16. Raman spectra of VCoNiPBA with different Co/Ni ratios


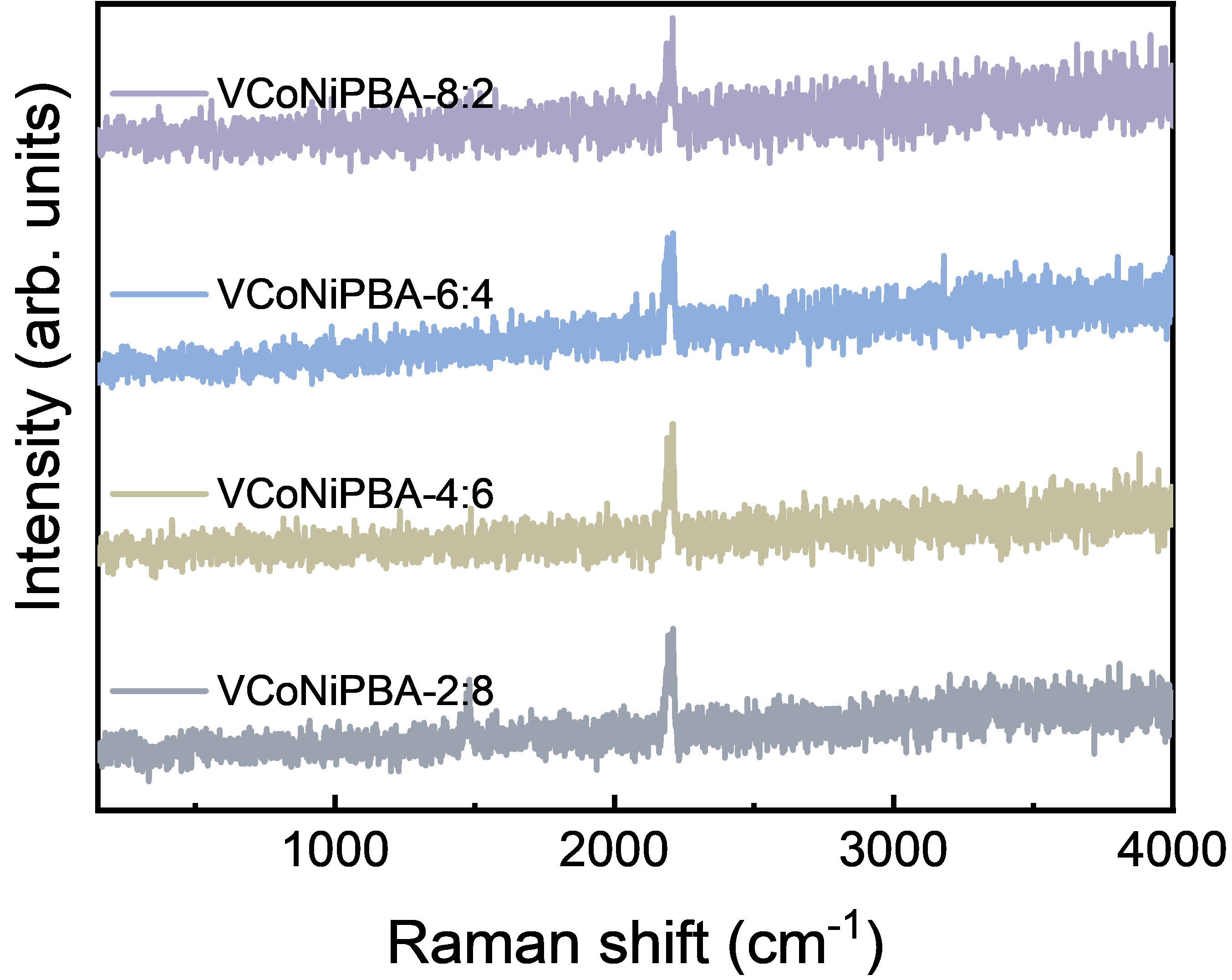


**Figure S15.** Raman spectra of VCoNiPBA with different Co/Ni ratios.

# 17. XPS survey spectra


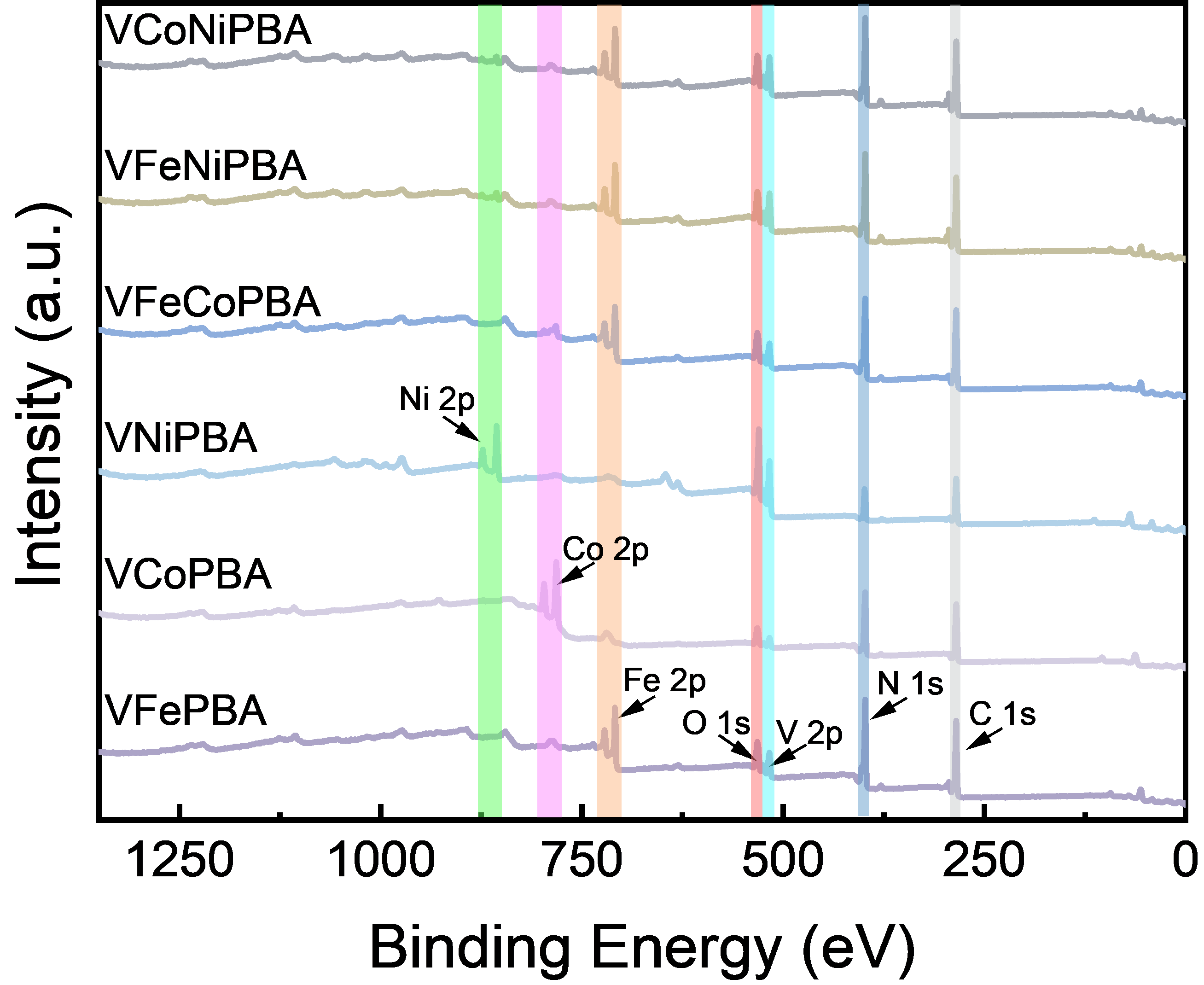


**Figure S16.** XPS survey spectra of samples with different elemental compositions.

# 18. Fe 2p XPS spectrum of VFePBA-25^o^C.


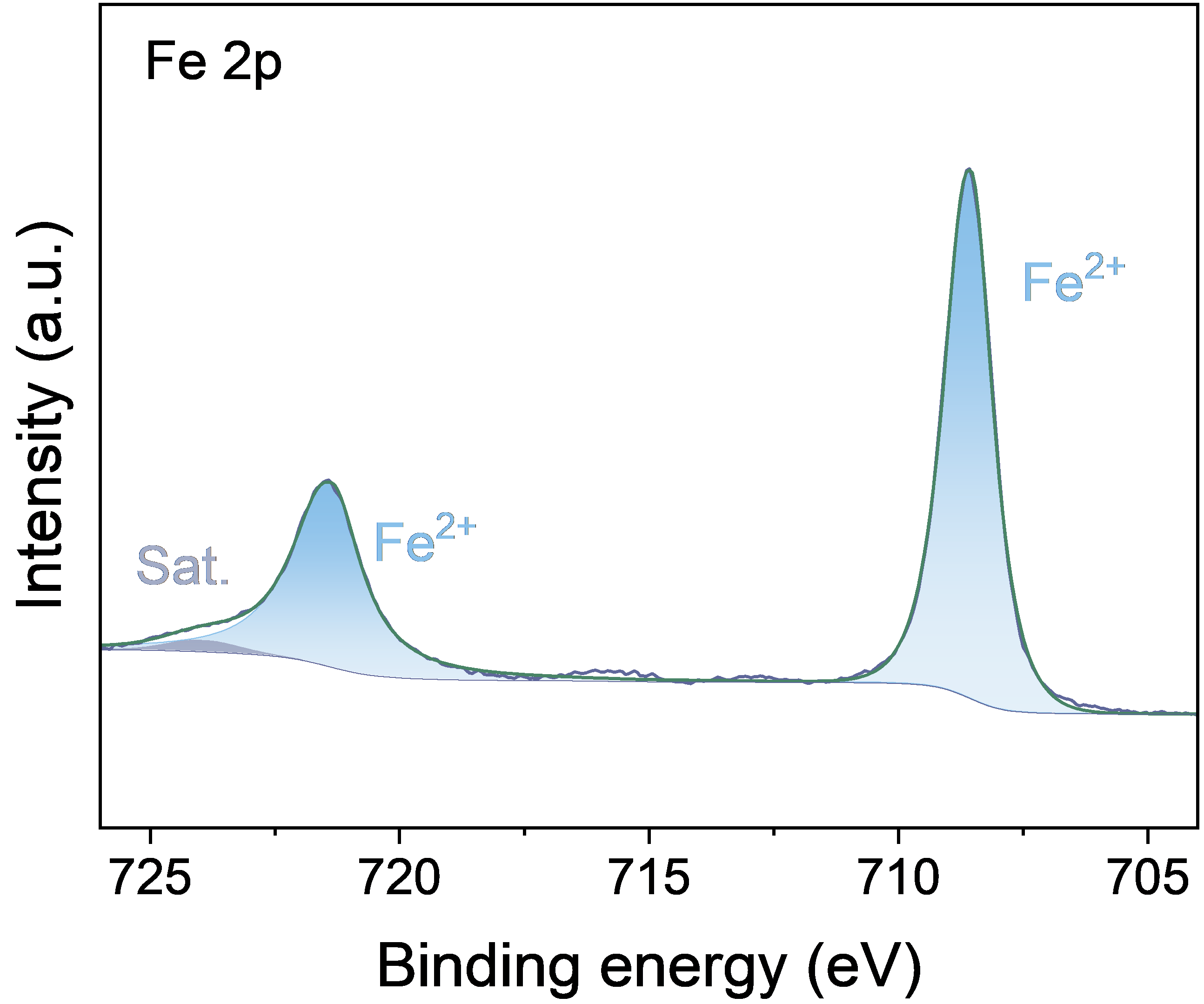


**Figure S17.** Fe 2p XPS spectrum of VFePBA-25^o^C.

# 19. Fe 2p XPS spectrum of VFeCoPBA


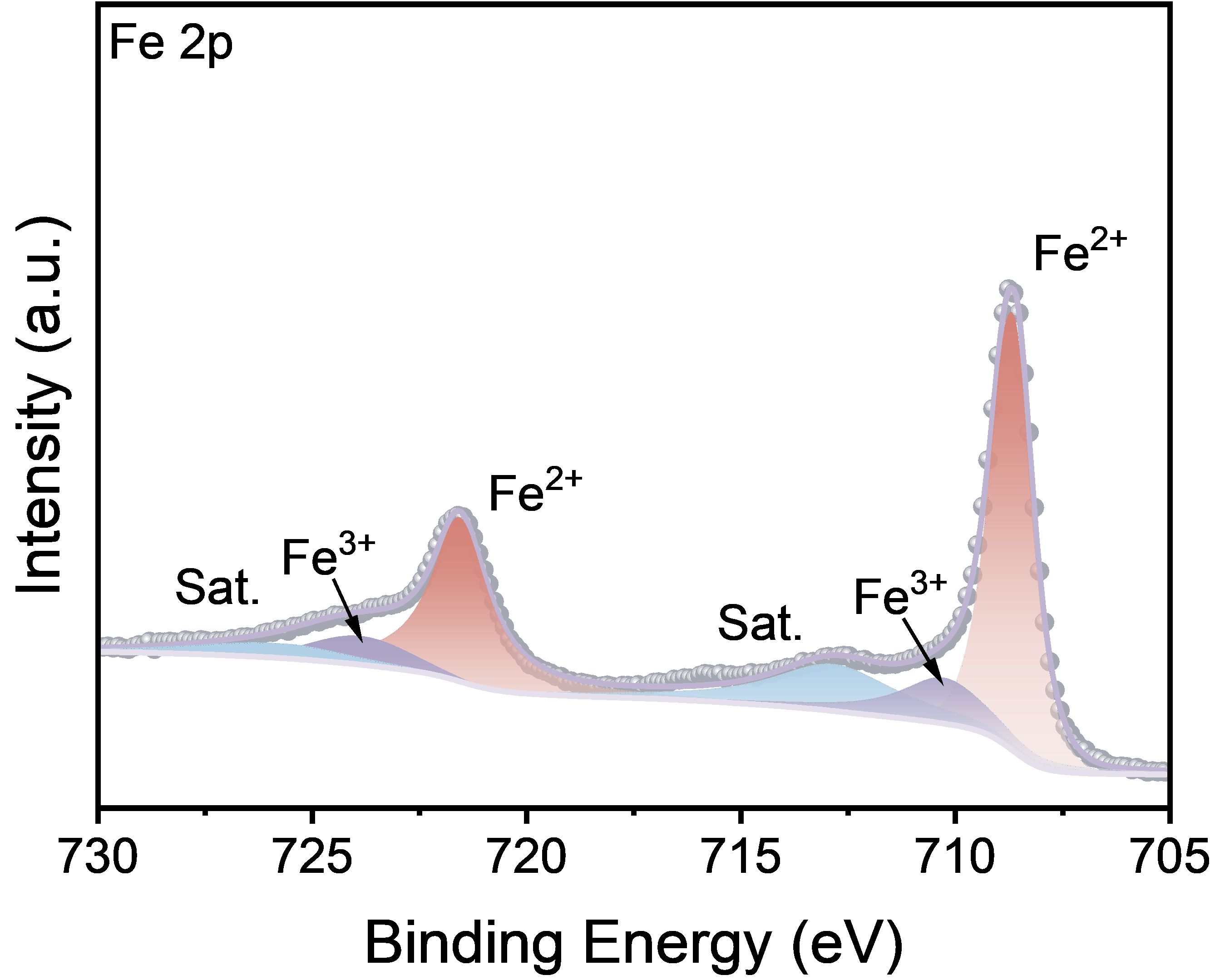


**Figure S18**. Fe 2p XPS spectrum of VFeCoPBA.

# 20. Fe 2p XPS spectrum of VFeNiPBA


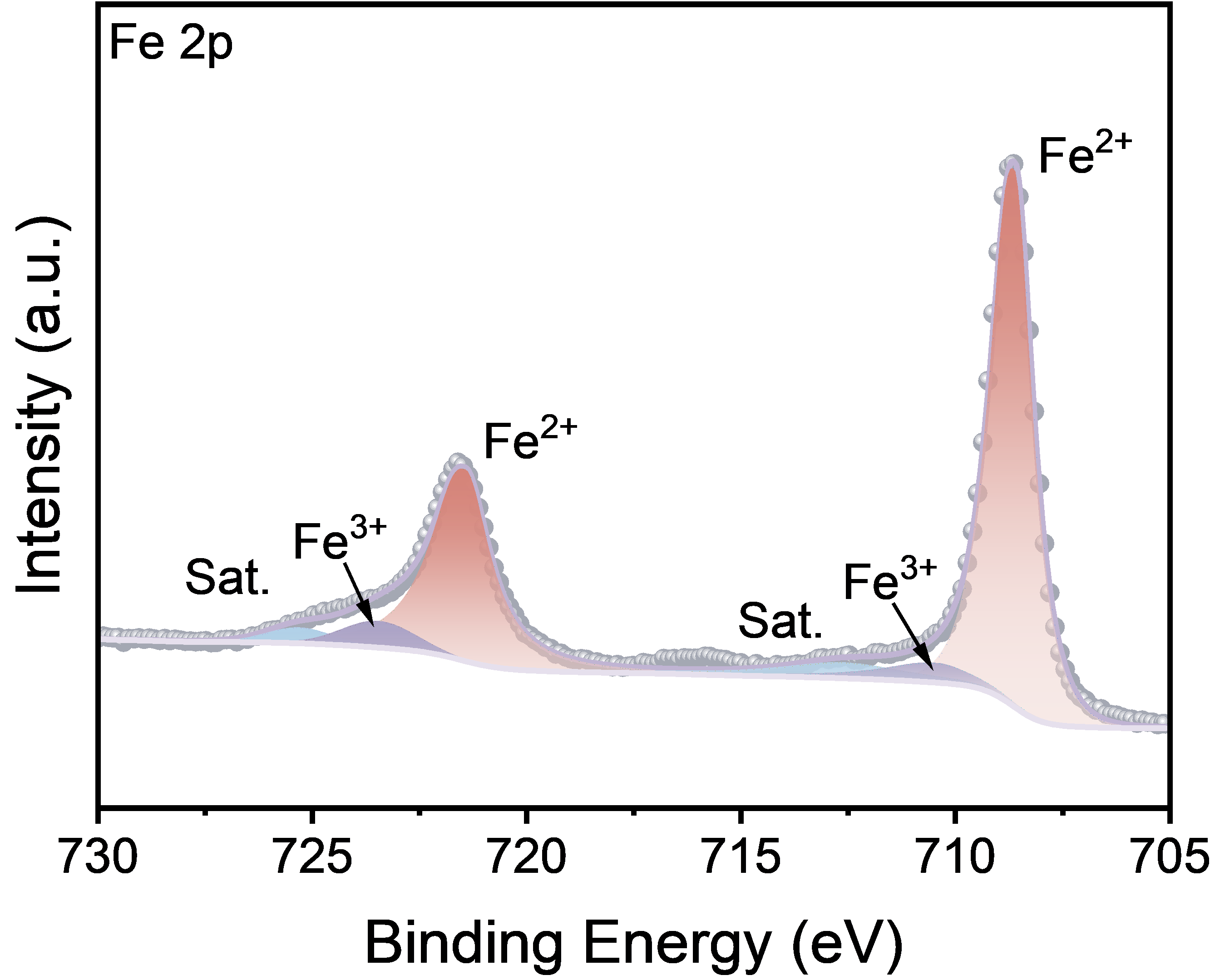


**Figure S19.** Fe 2p XPS spectrum of VFeNiPBA.

# 21. Co 2p XPS spectrum of VFeCoPBA


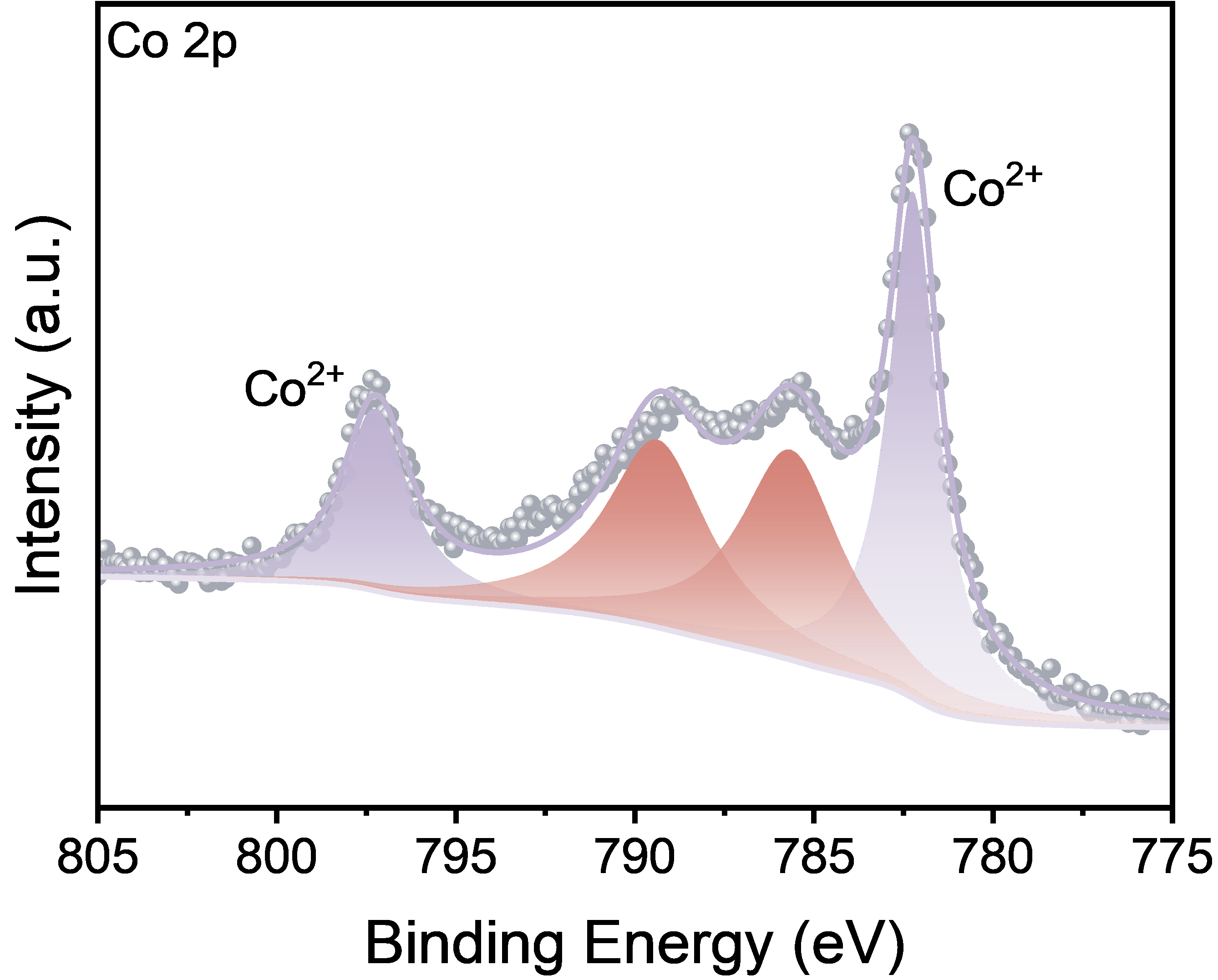


**Figure S20.** Co 2p XPS spectrum of VFeCoPBA.

# 22. Co 2p XPS spectrum of VCoNiPBA


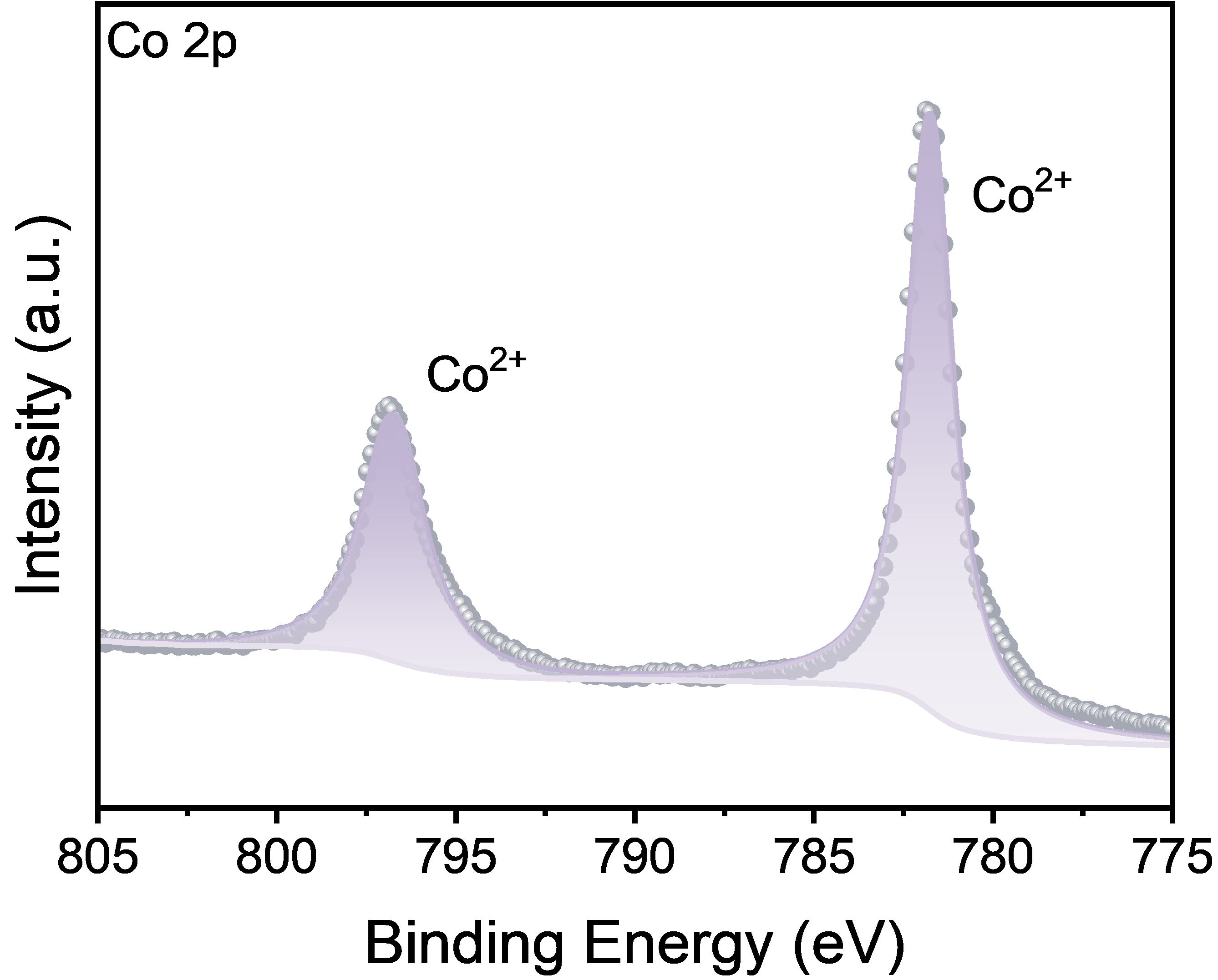


**Figure S21.** Co 2p XPS spectrum of VCoNiPBA.

# 23. Ni 2p XPS spectrum of VFeNiPBA


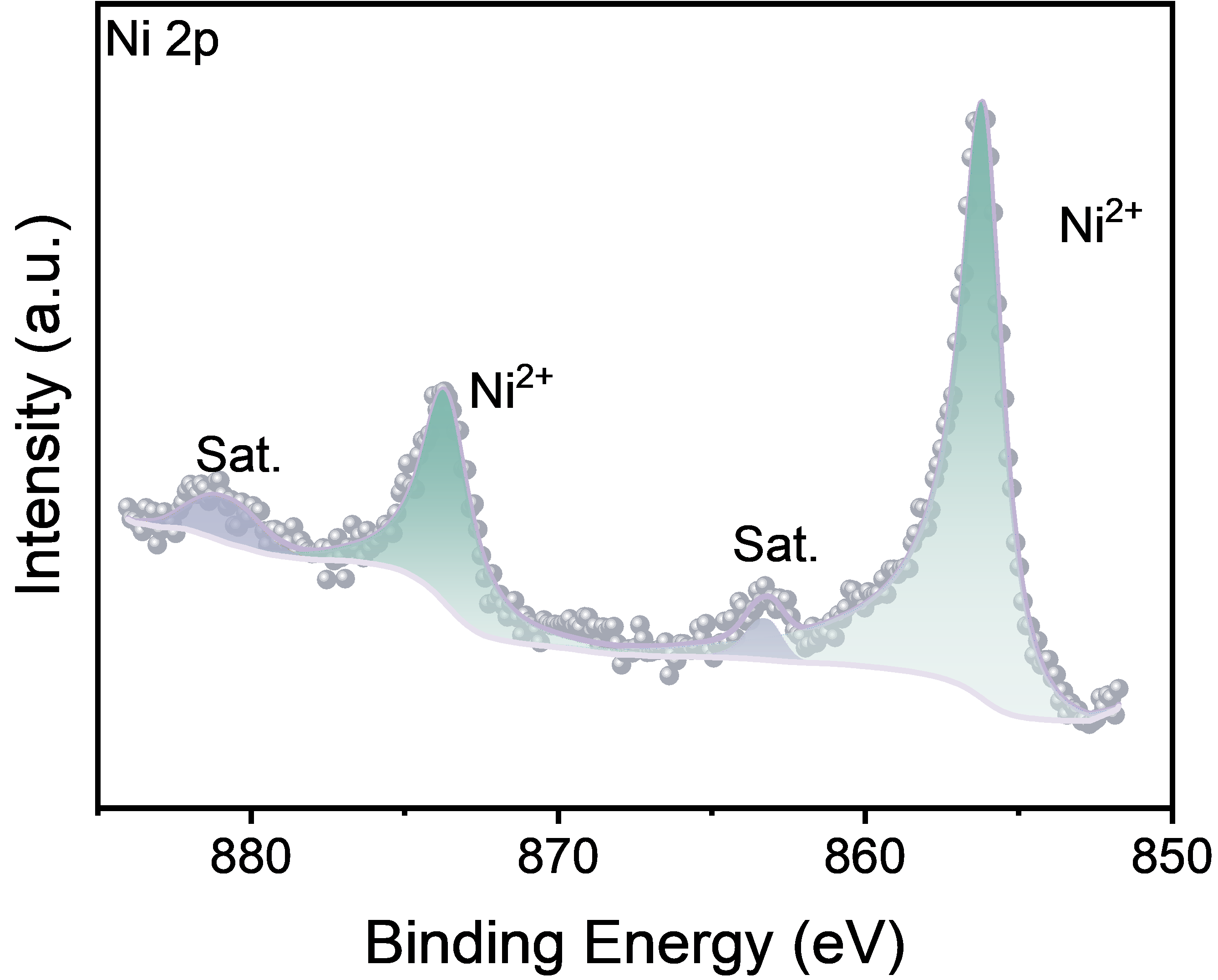


**Figure S22.** Ni 2p XPS spectrum of VFeNiPBA.

# 24. Ni 2p XPS spectrum of VCoNiPBA


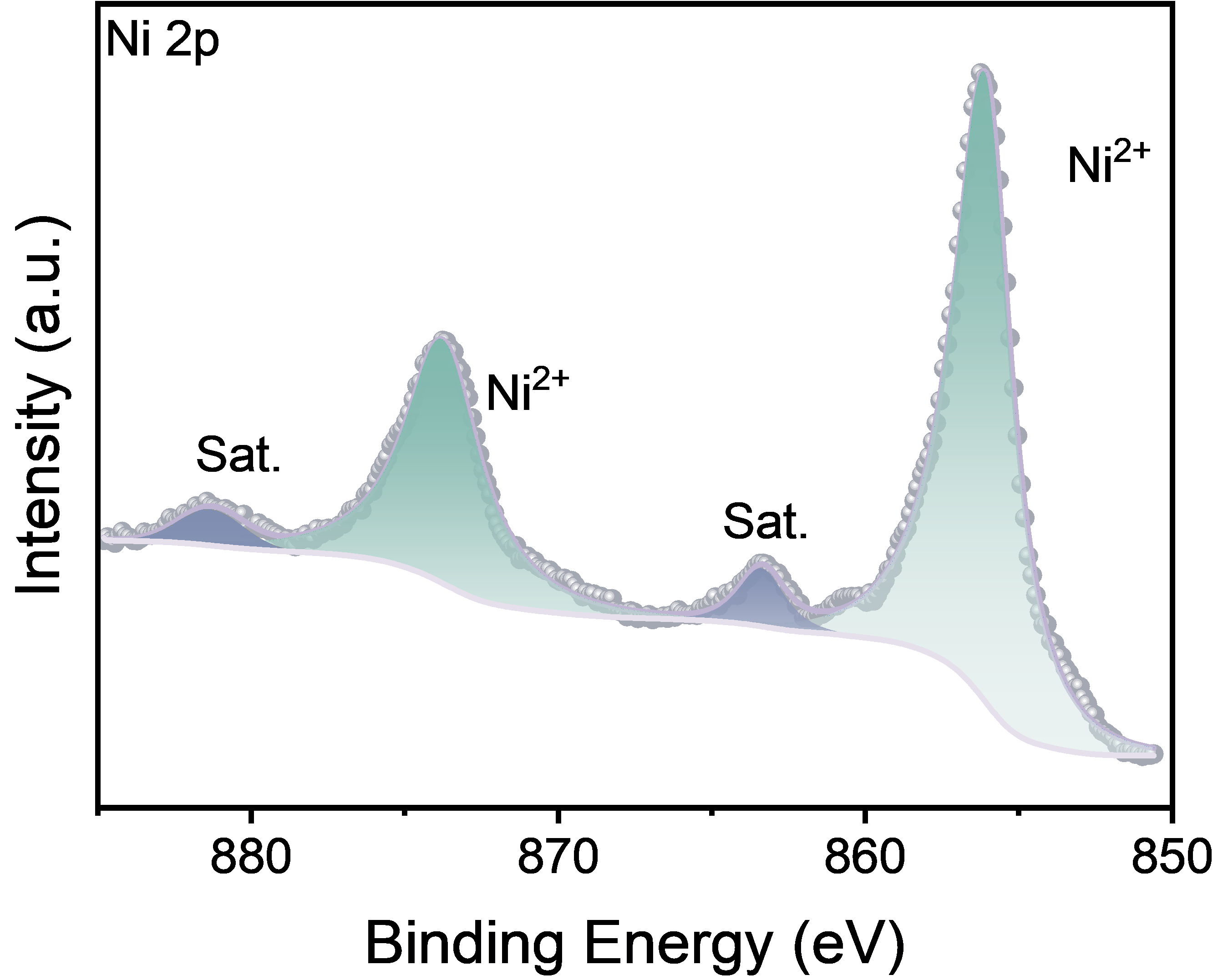


**Figure S23.** Ni 2p XPS spectrum of VCoNiPBA.

# 25. V 2p XPS spectrum of VFePBA-25^o^C.


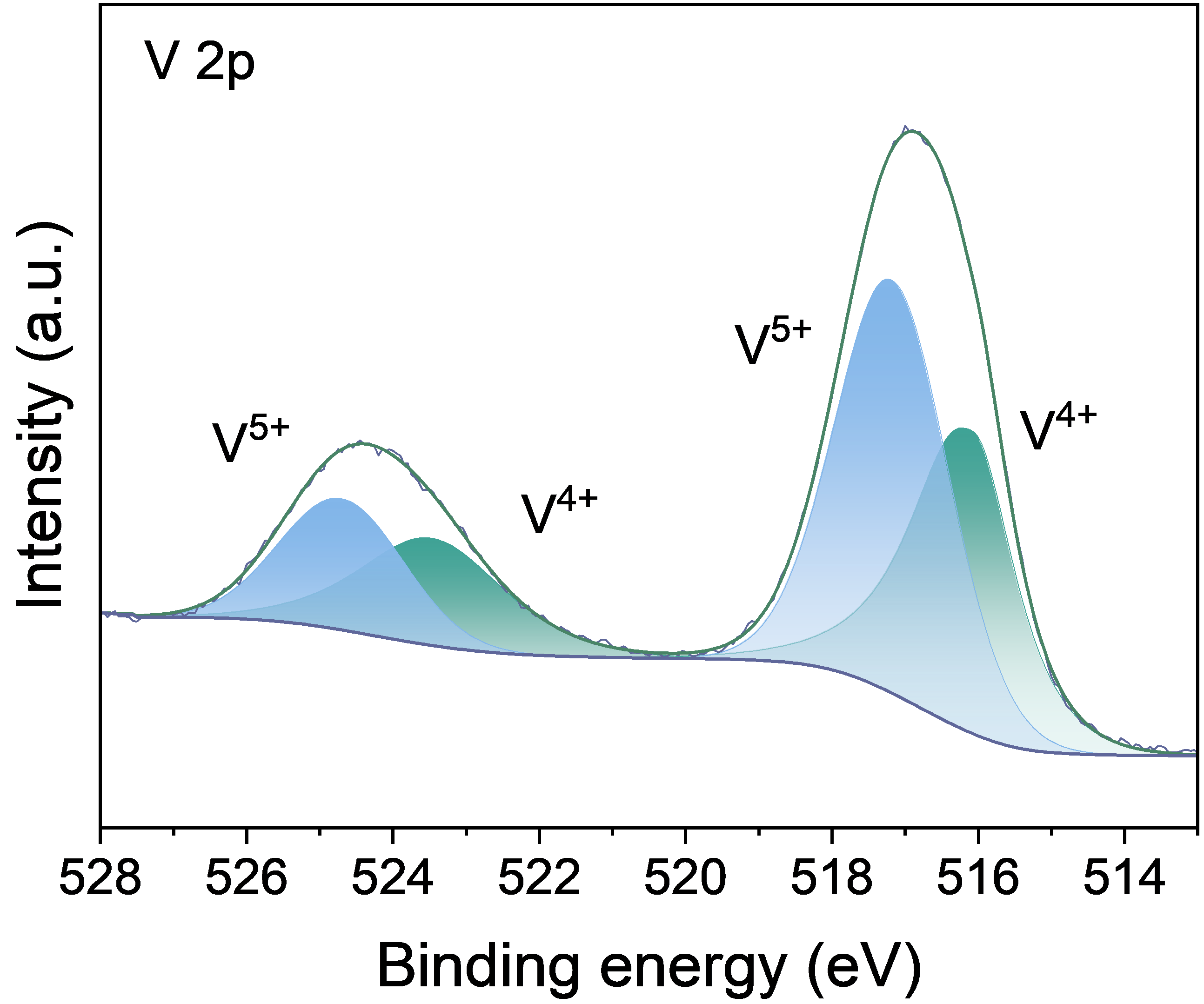


**Figure S24.** V 2p XPS spectrum of VFePBA-25^o^C.

# 26. BET surface area and pore size distribution


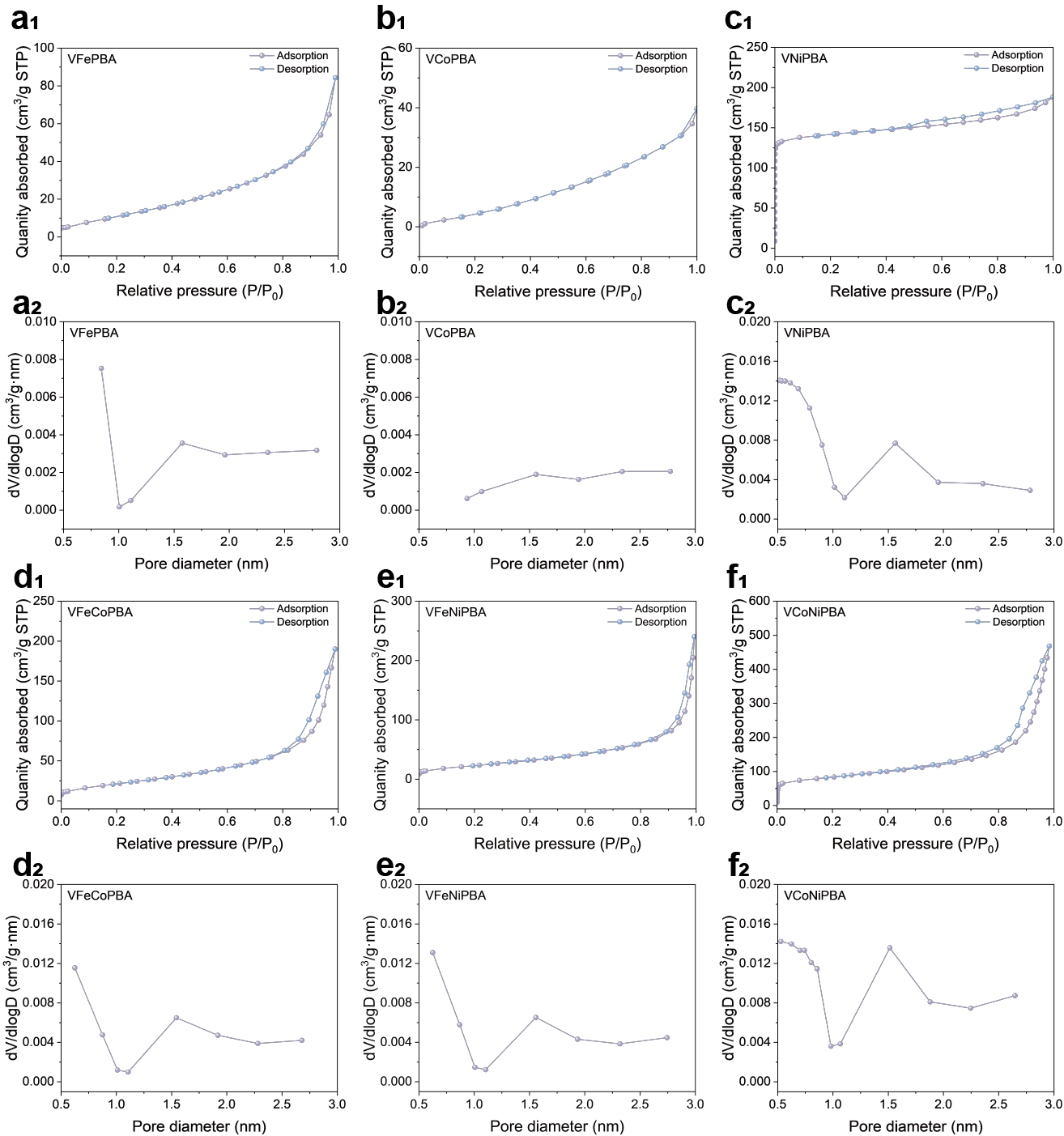


**Figure S25.** BET surface area and pore size distribution of samples with different elemental compositions.

# 27. CV Profiles of VFeCoPBA with Varied Fe/Co Ratios


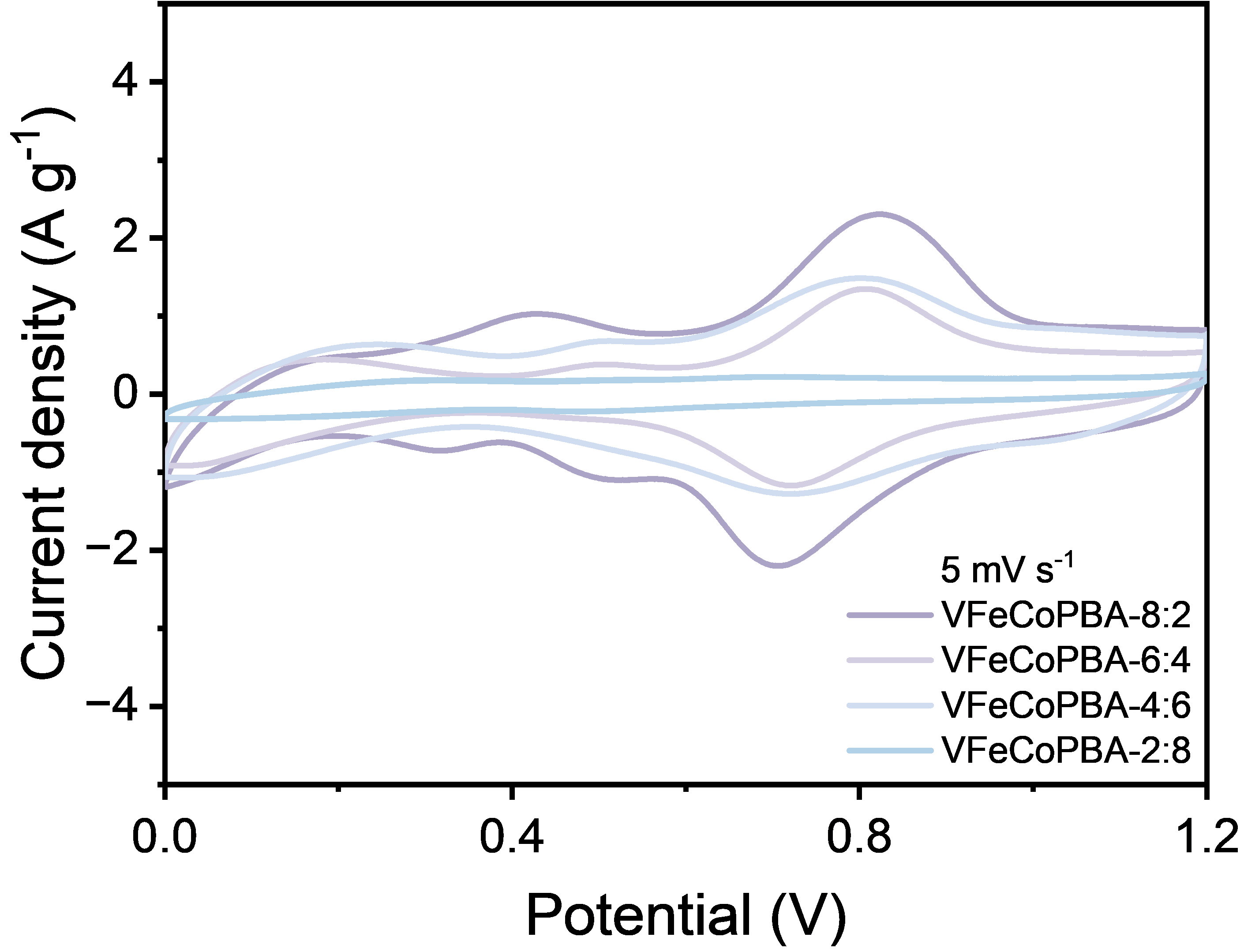


**Figure S26.** CV Profiles of VFeCoPBA with Varied Fe/Co Ratios at 5 mV s^-1^.

# 28. CV Profiles of VFeNiPBA with Varied Fe/Co Ratios


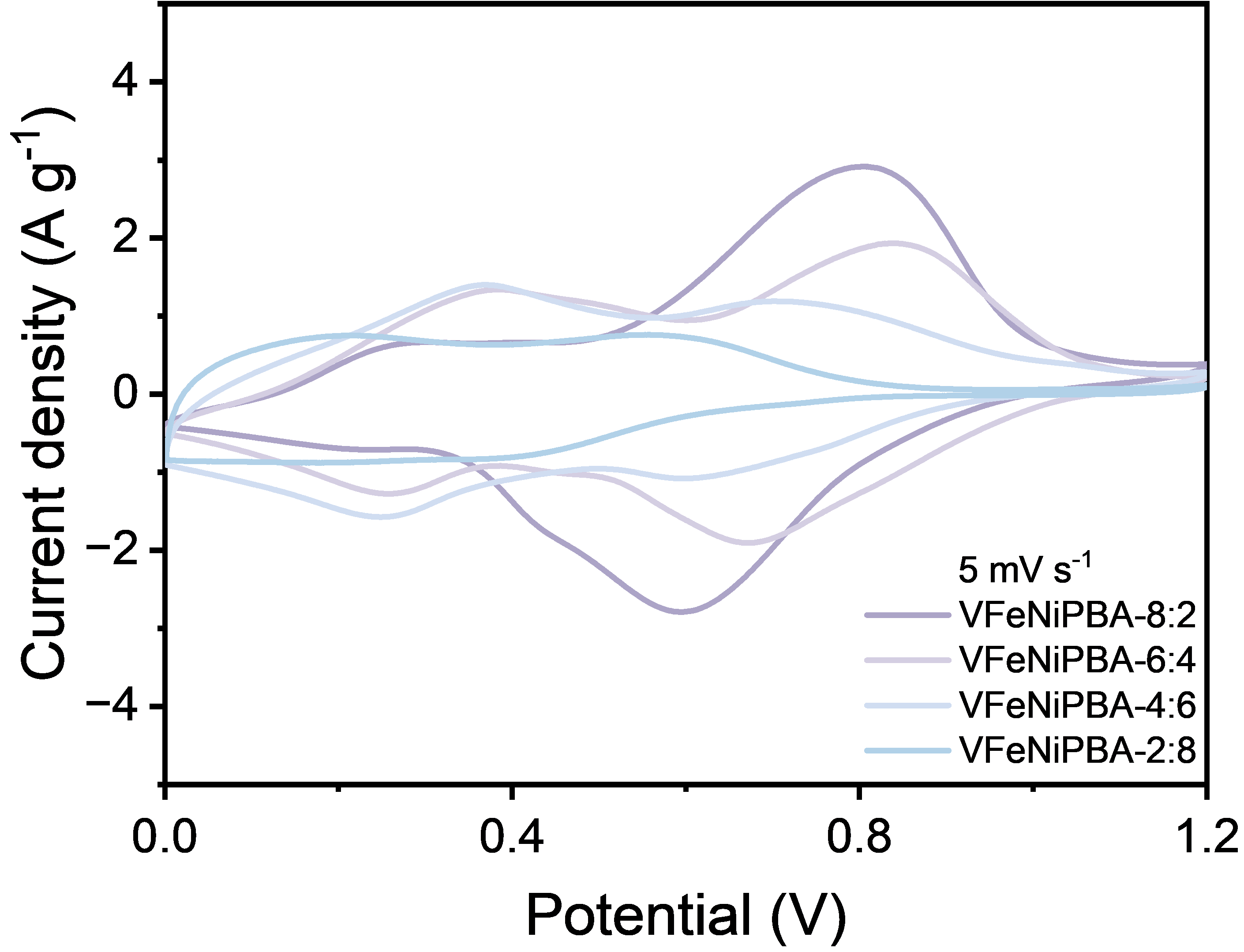


**Figure S27.** CV Profiles of VFeNiPBA with Varied Fe/Co Ratios at 5 mV s^-1^.

# 29. Galvanostatic Charge-Discharge Profiles of VFeCoPBA with Varied Fe/Co Ratios


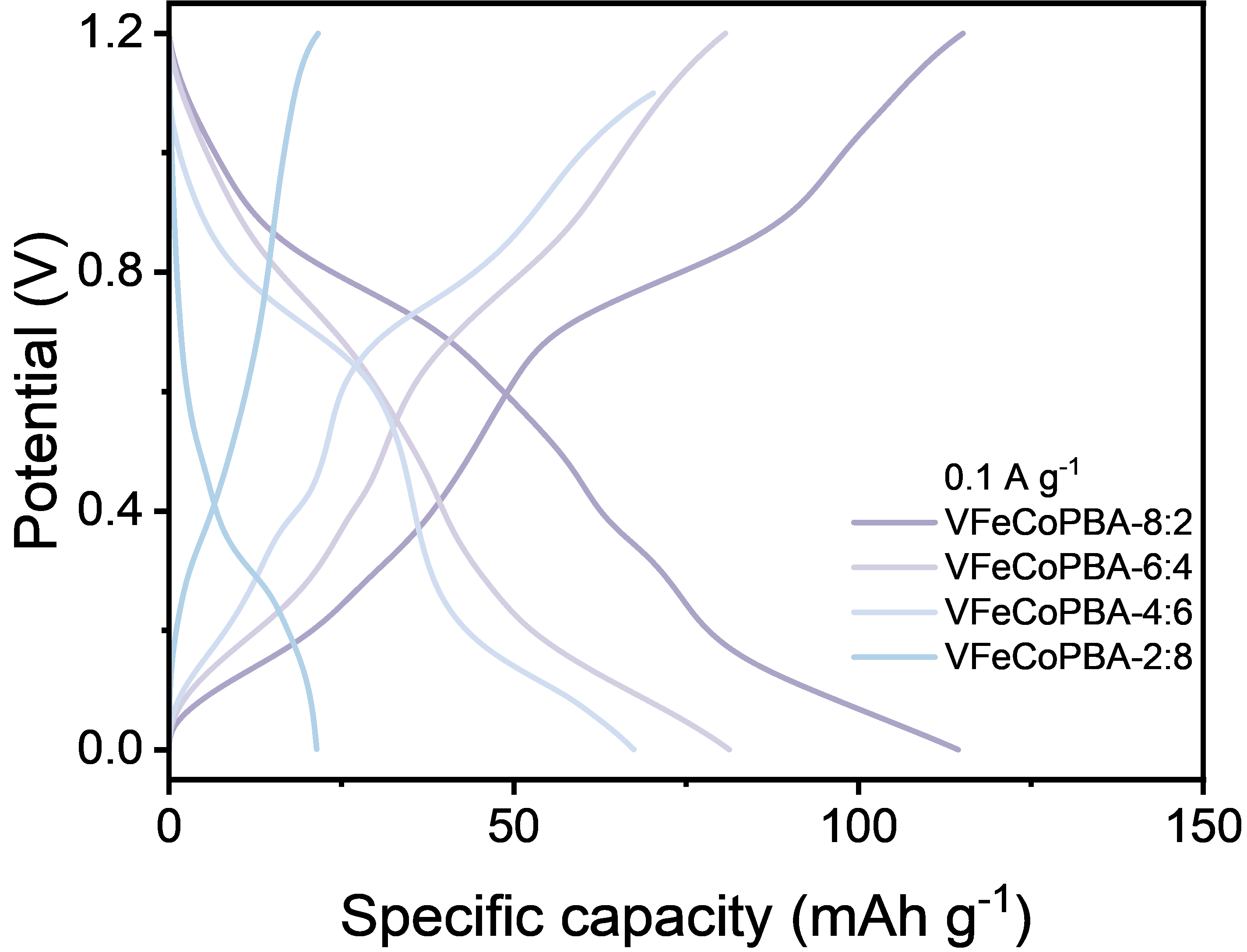


**Figure S28.** Galvanostatic Charge-Discharge Profiles of VFeCoPBA with Varied Fe/Co Ratios at 0.1 A g^-1^.

# 30. Galvanostatic Charge-Discharge Profiles of VFeNiPBA with Varied Fe/Co Ratios


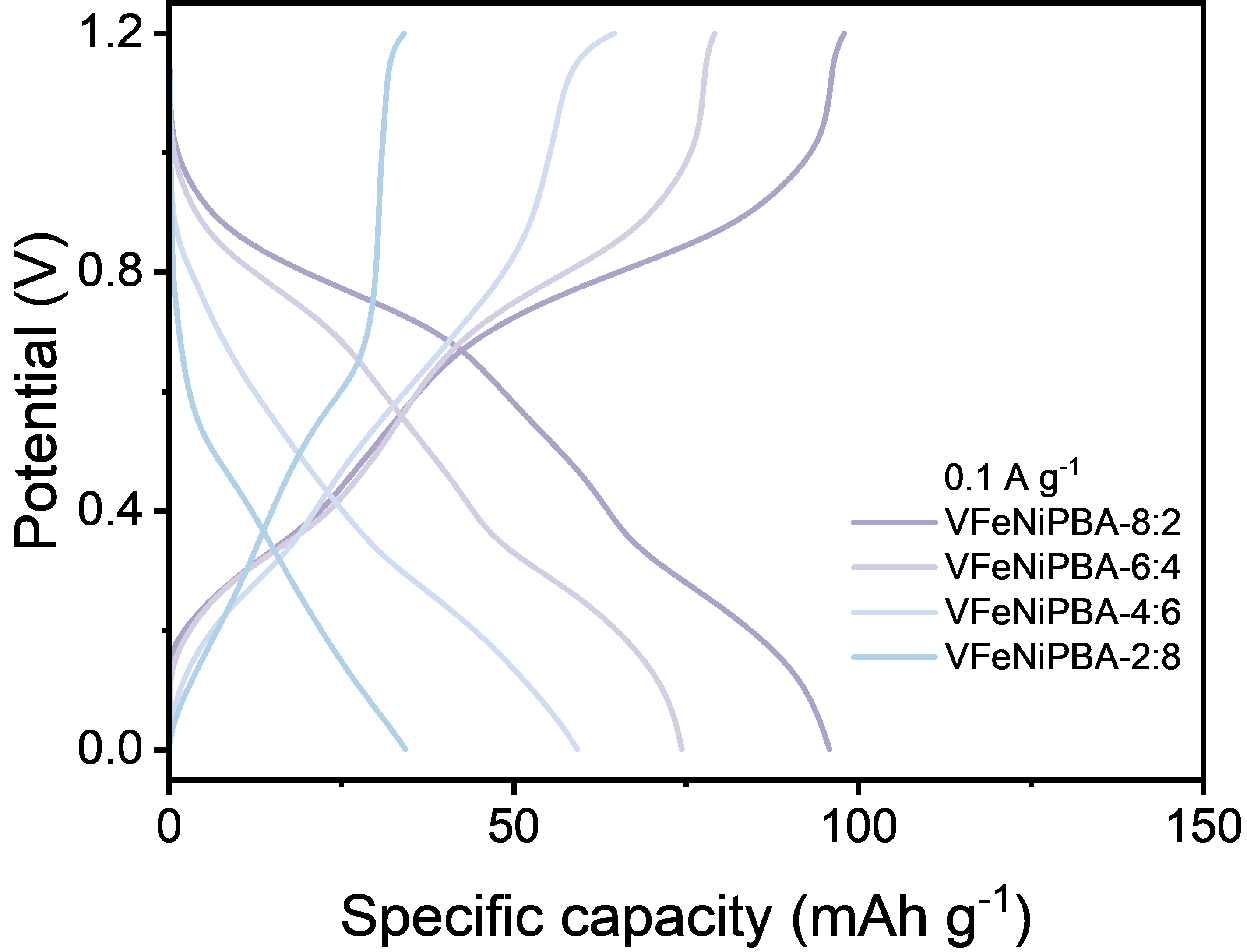


**Figure S29.** Galvanostatic Charge-Discharge Profiles of VFeNiPBA with Varied Fe/Co Ratios at 0.1 A g^-1^.

# 31. CV curves, b values, and Capacitive Contribution Ratio of VFePBA


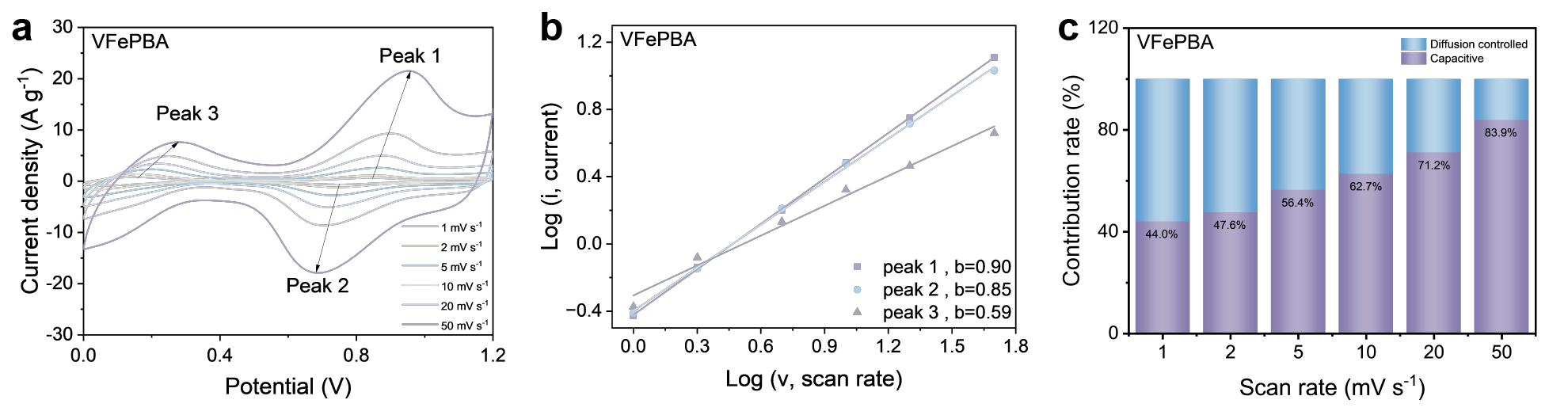


**Figure S30.** (a) CV curves at various scan rates, (b) The linear fitting plots of the b values from CV curves, (c) Capacitive Contribution Ratio at Different Scan Rates of VFePBA.

# 32. Capacitive Contribution Ratio at different scan rates of VFePBA


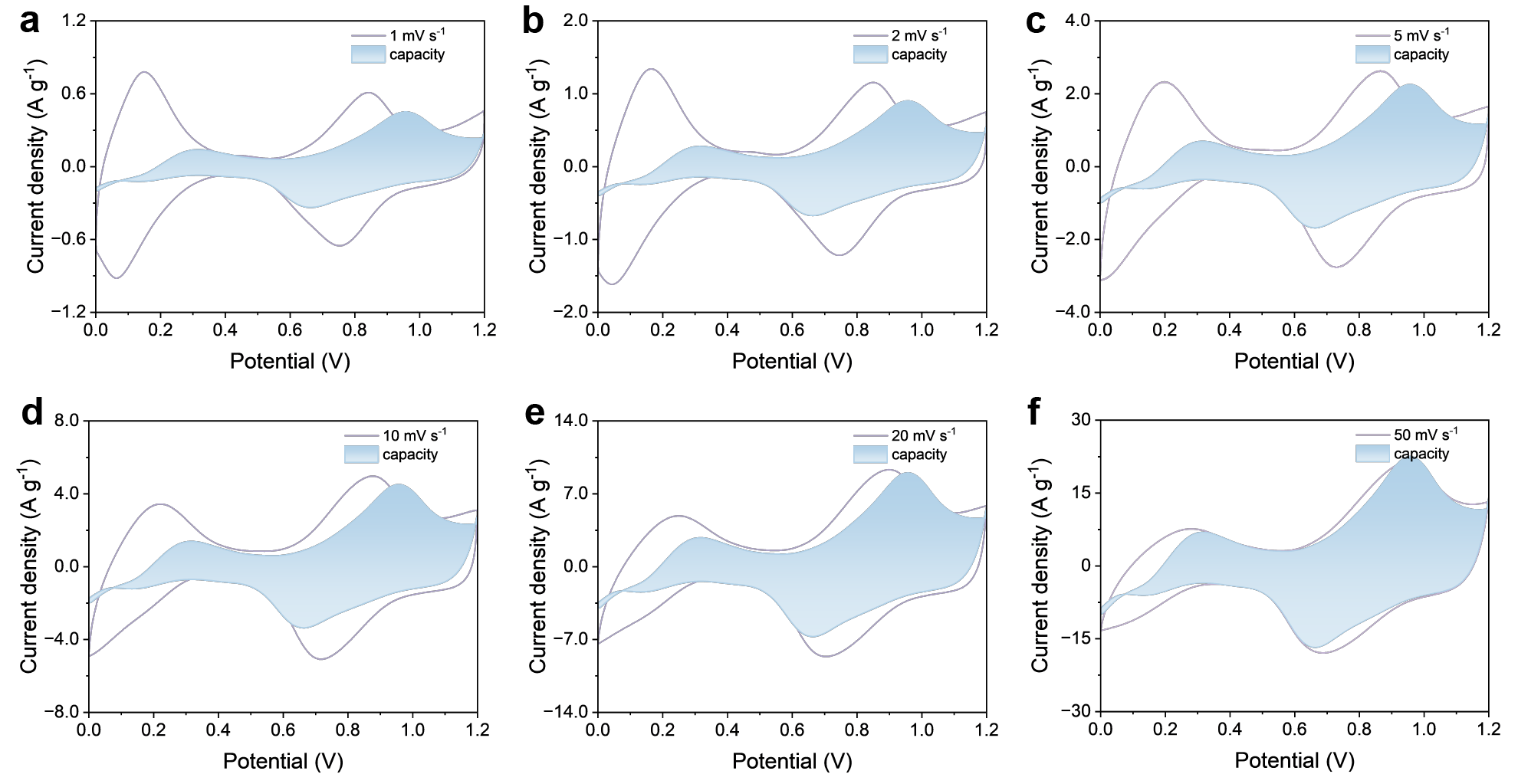


**Figure S31.** Capactitive contribution to total storage for the VFePBA electrode at different scan rates: (a) 1 mV·s^-1^, (b) 2 mV·s^-1^, (c) 5 mV·s^-1^, (d) 10 mV·s^-1^, (e) 20 mV·s^-1^, and (f) 50 mV·s^-1^.

# 33. CV curves, b values, and Capacitive Contribution Ratio of VFeCoPBA-8:2


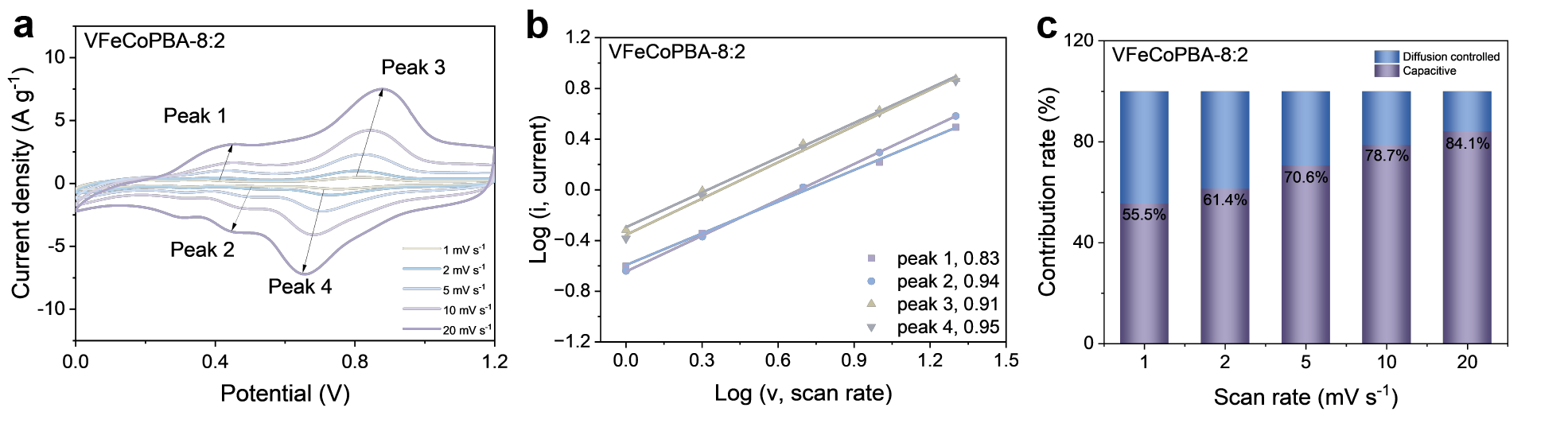


**Figure S32.** (a) CV curves at various scan rates, (b) The linear fitting plots of the b values from CV curves, (c) Capacitive Contribution Ratio at Different Scan Rates of VFeCoPBA-8:2.

# 34. Capacitive Contribution Ratio at different scan rates of VFeCoPBA-8:2


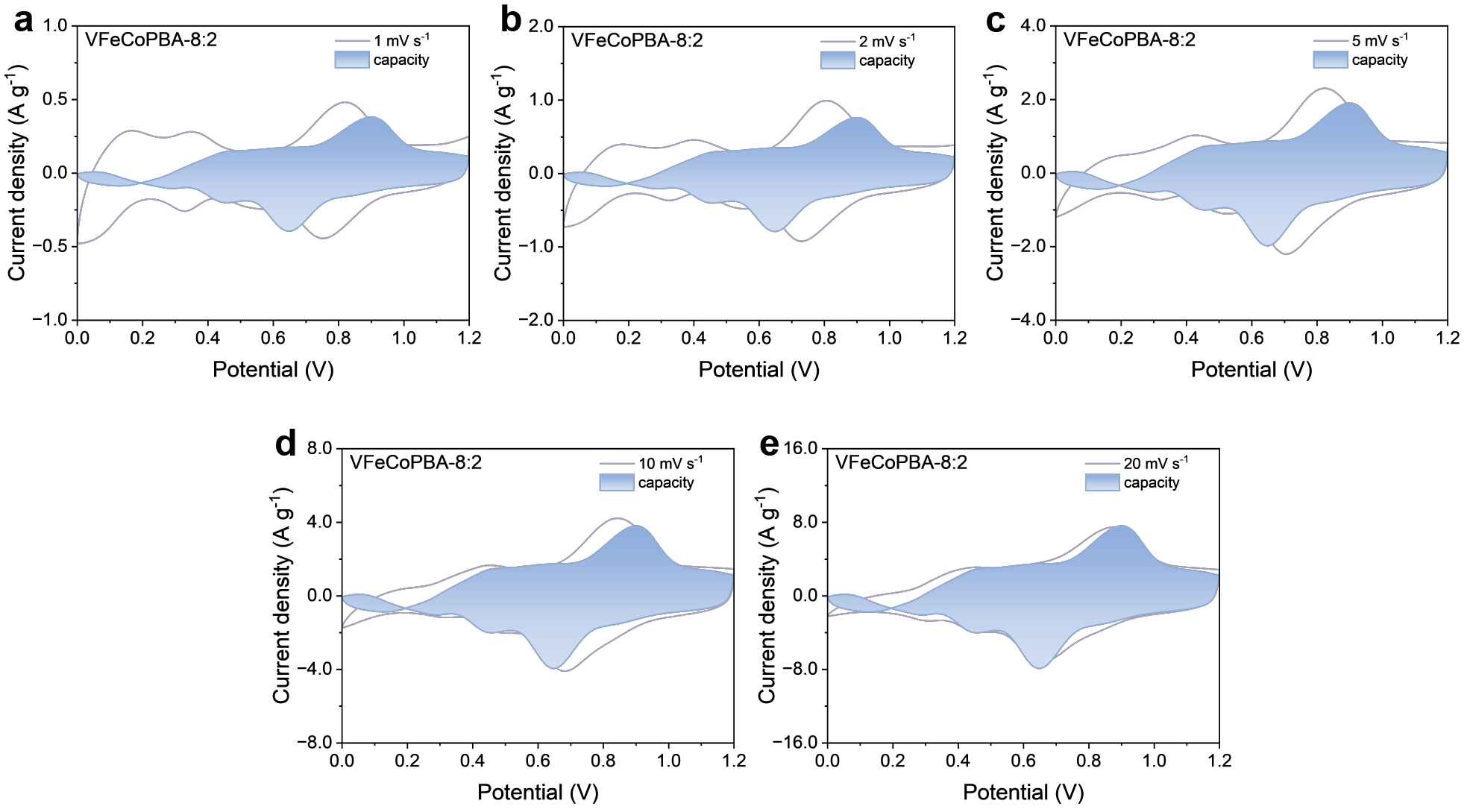


**Figure S33.** Capactitive contribution to total storage for the VFeCoPBA-8:2 electrode at different scan rates: (a) 1 mV·s^-1^, (b) 2 mV·s^-1^, (c) 5 mV·s^-1^, (d) 10 mV·s^-1^, and (e) 20 mV·s^-1^.

# 35. CV curves, b values, and Capacitive Contribution Ratio of VFeCoPBA-6:4


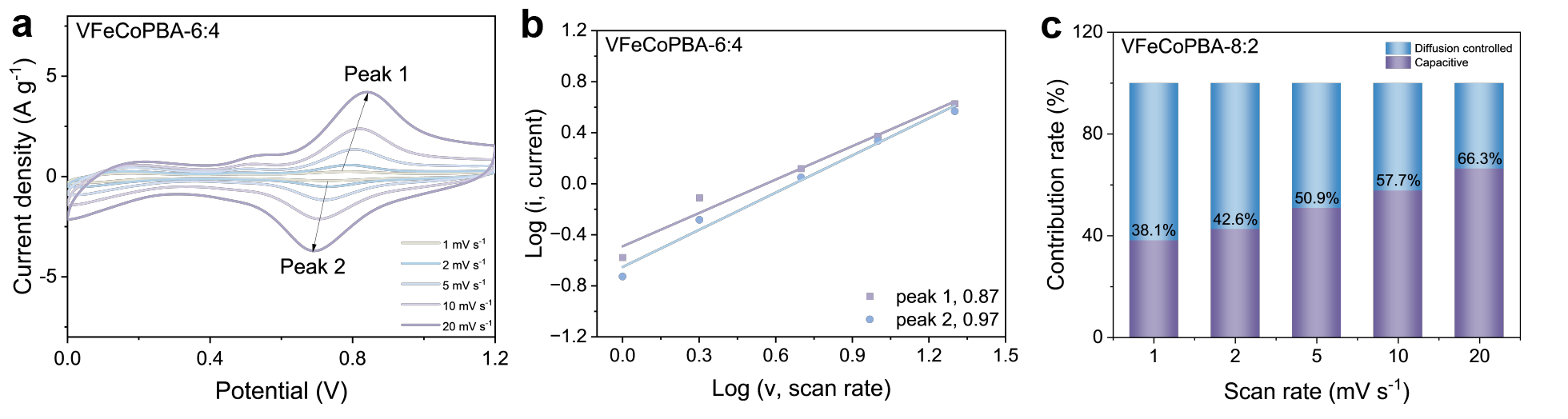


**Figure S34.** (a) CV curves at various scan rates, (b) The linear fitting plots of the b values from CV curves, (c) Capacitive Contribution Ratio at Different Scan Rates of VFeCoPBA-6:4.

# 36. Capacitive Contribution Ratio at different scan rates of VFeCoPBA-6:4


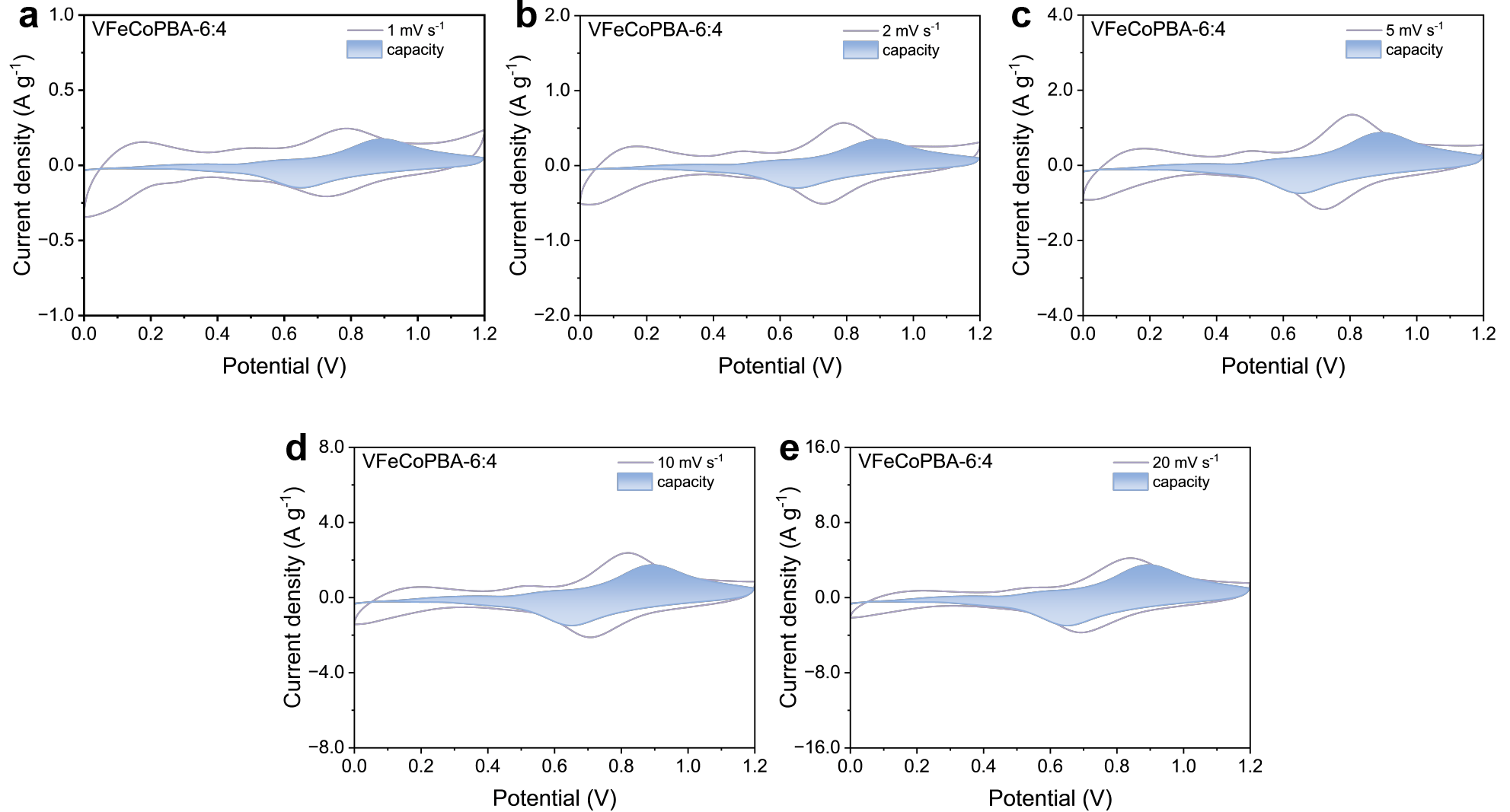


**Figure S35.** Capactitive contribution to total storage for the VFeCoPBA-6:4 electrode at different scan rates: (a) 1 mV·s^-1^, (b) 2 mV·s^-1^, (c) 5 mV·s^-1^, (d) 10 mV·s^-1^, and (e) 20 mV·s^-1^.

# 37. CV curves, b values, and Capacitive Contribution Ratio of VFeCoPBA-4:6


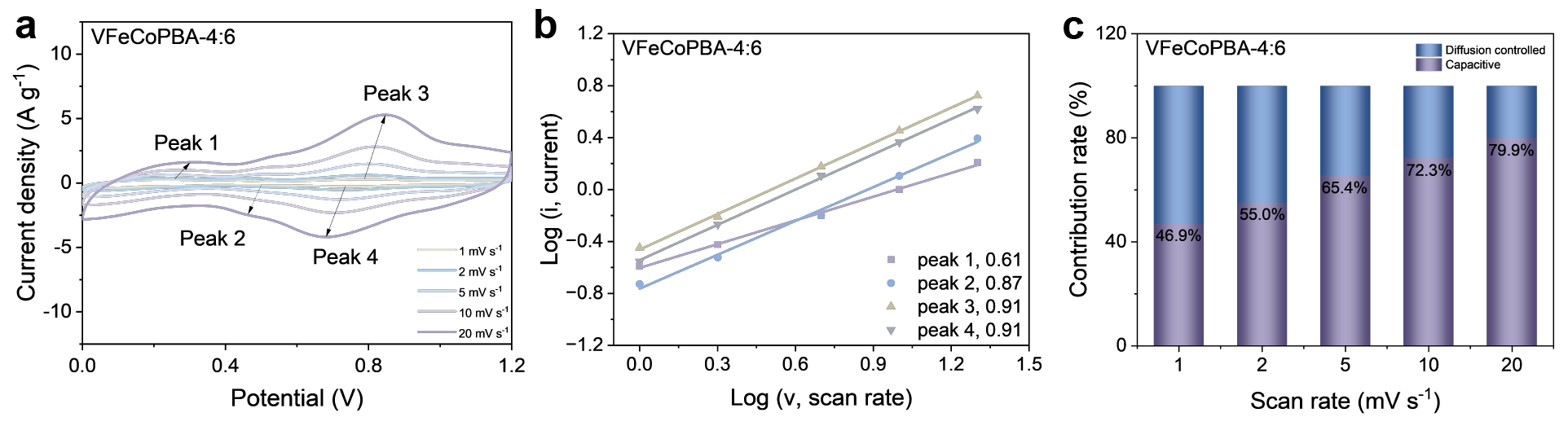


**Figure S36.** (a) CV curves at various scan rates, (b) The linear fitting plots of the b values from CV curves, (c) Capacitive Contribution Ratio at Different Scan Rates of VFeCoPBA-4:6.

# 38. Capacitive Contribution Ratio at different scan rates of VFeCoPBA-4:6


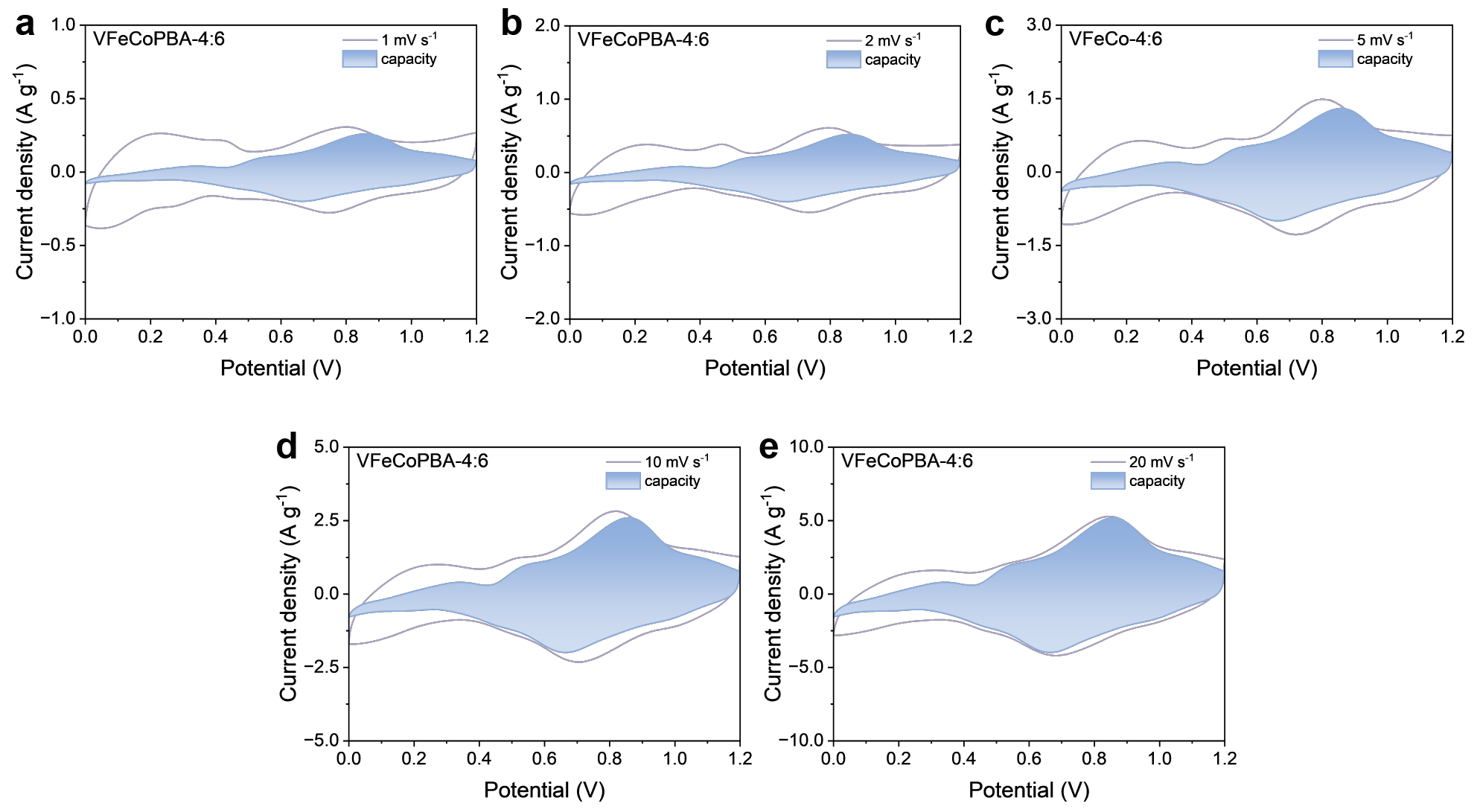


**Figure S37.** Capactitive contribution to total storage for the VFeCoPBA-4:6 electrode at different scan rates: (a) 1 mV·s^-1^, (b) 2 mV·s^-1^, (c) 5 mV·s^-1^, (d) 10 mV·s^-1^, and (e) 20 mV·s^-1^.

# 39. CV curves, b values, and Capacitive Contribution Ratio of VFeCoPBA-2:8


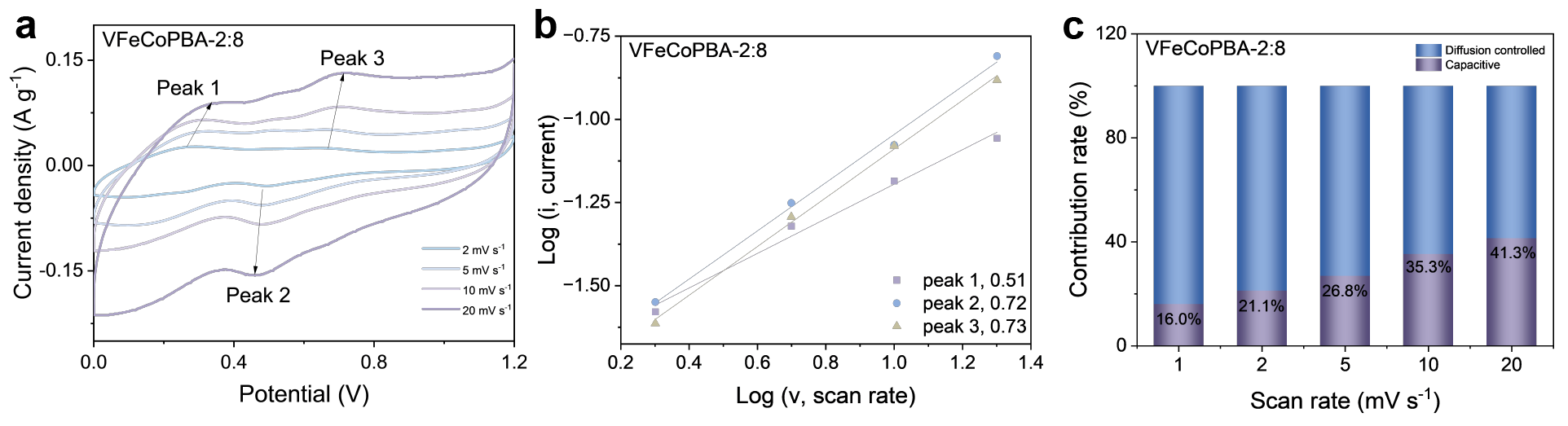


**Figure S38.** (a) CV curves at various scan rates, (b) The linear fitting plots of the b values from CV curves, (c) Capacitive Contribution Ratio at Different Scan Rates of VFeCoPBA-2:8.

# 40. Capacitive Contribution Ratio at different scan rates of VFeCoPBA-2:8


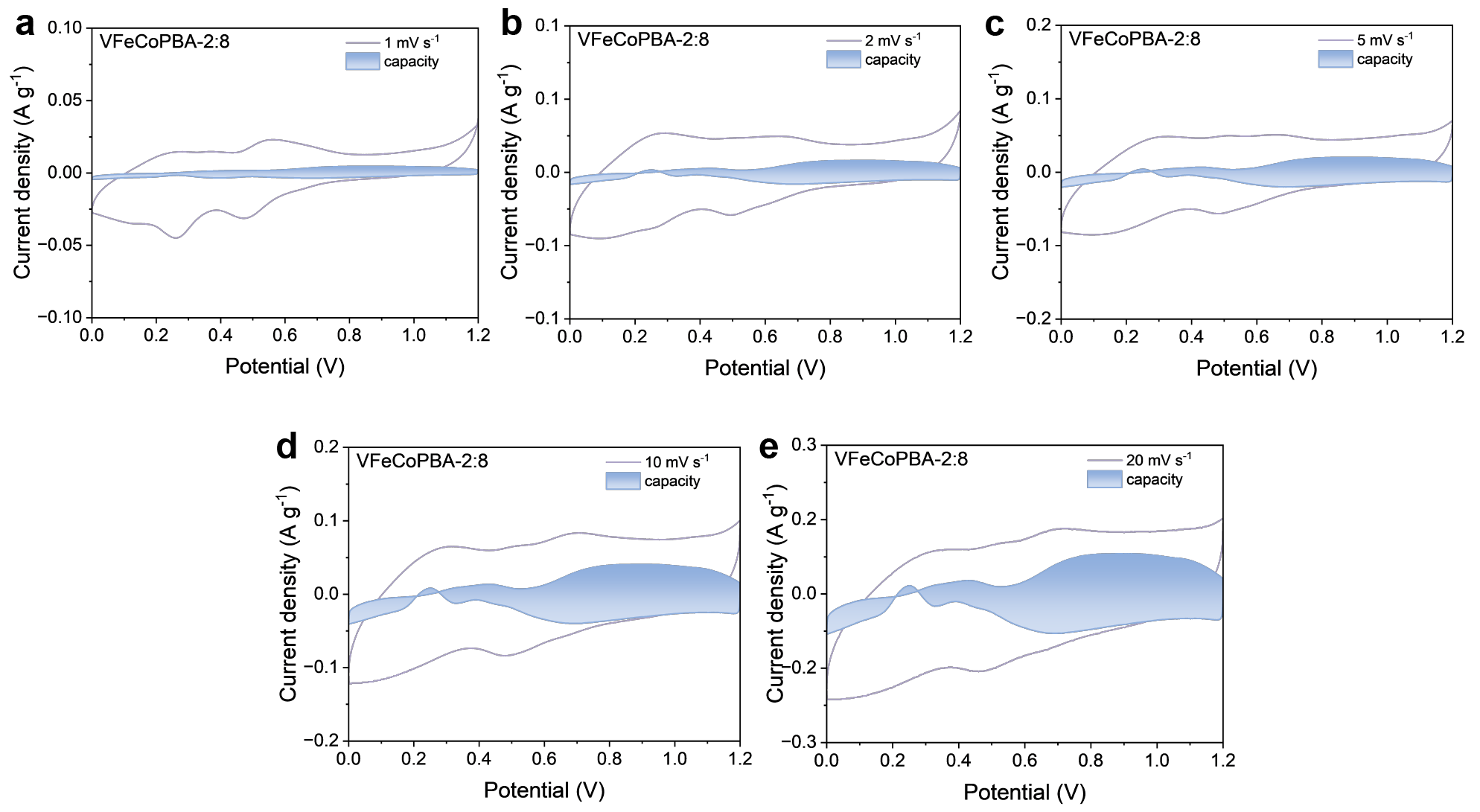


**Figure S39.** Capactitive contribution to total storage for the VFeCoPBA-2:8 electrode at different scan rates: (a) 1 mV·s^-1^, (b) 2 mV·s^-1^, (c) 5 mV·s^-1^, (d) 10 mV·s^-1^, and (e) 20 mV·s^-1^.

# 41. CV curves, b values, and Capacitive Contribution Ratio of VFeNiPBA-8:2


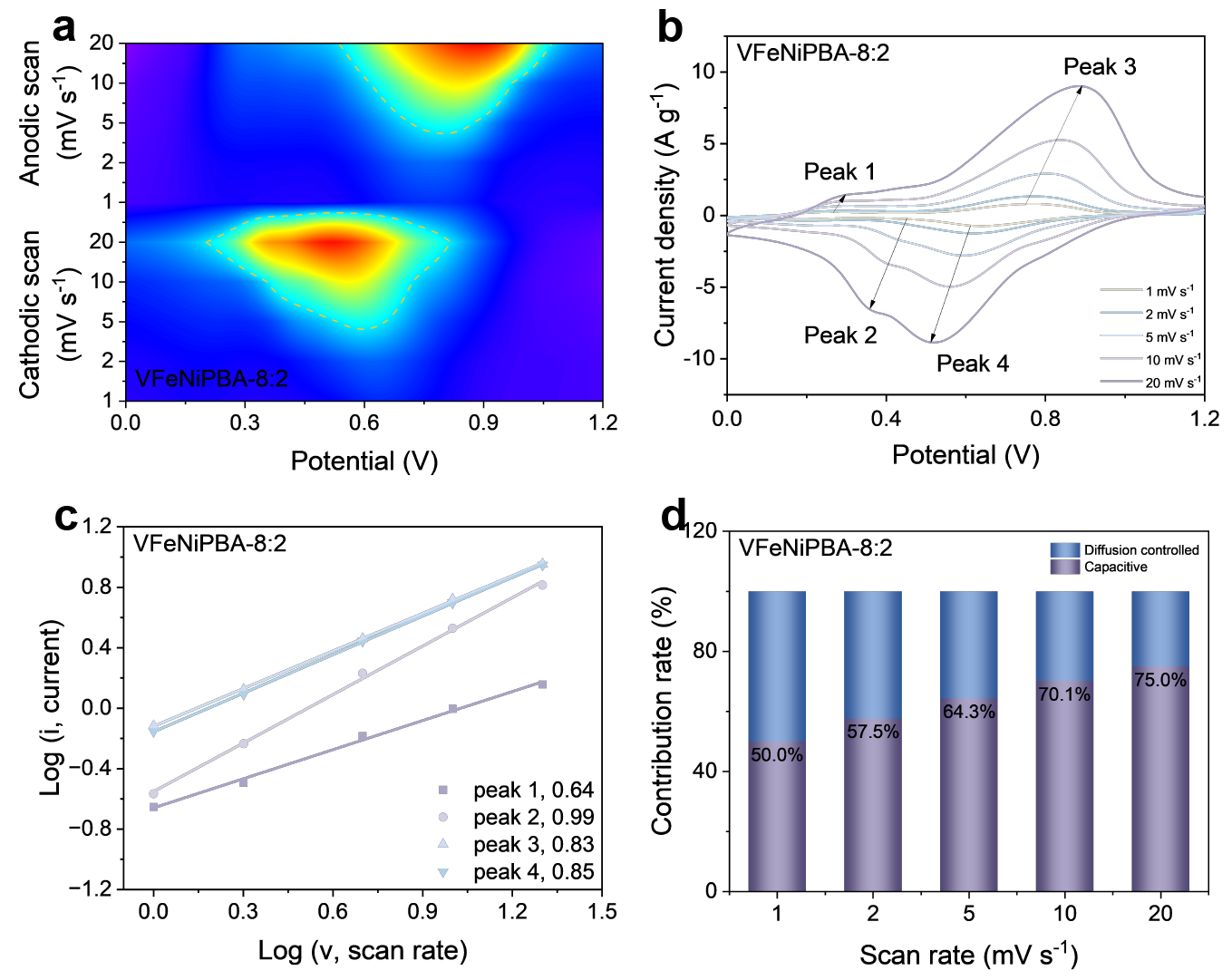


**Figure S40.** (a, b) CV curves at various scan rates, (c) The linear fitting plots of the b values from CV curves, (d) Capacitive Contribution Ratio at Different Scan Rates of VFeNiPBA-8:2.

# 42. Capacitive Contribution Ratio at different scan rates of VFeNiPBA-8:2


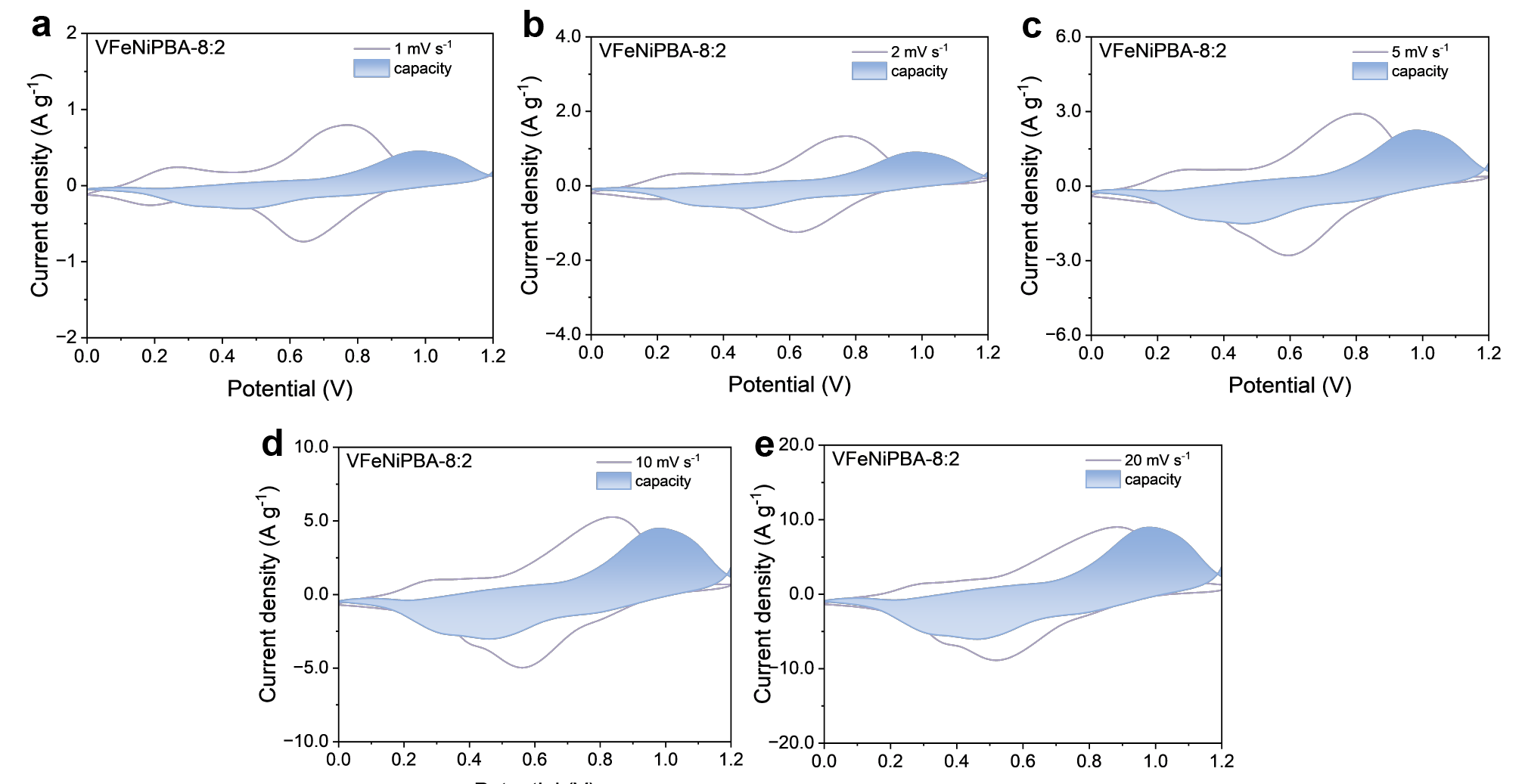


**Figure S41.** Capactitive contribution to total storage for the VFeNiPBA-8:2 electrode at different scan rates: (a) 1 mV·s^-1^, (b) 2 mV·s^-1^, (c) 5 mV·s^-1^, (d) 10 mV·s^-1^, and (e) 20 mV·s^-1^.

# 43. CV curves, b values, and Capacitive Contribution Ratio of VFeNiPBA-6:4


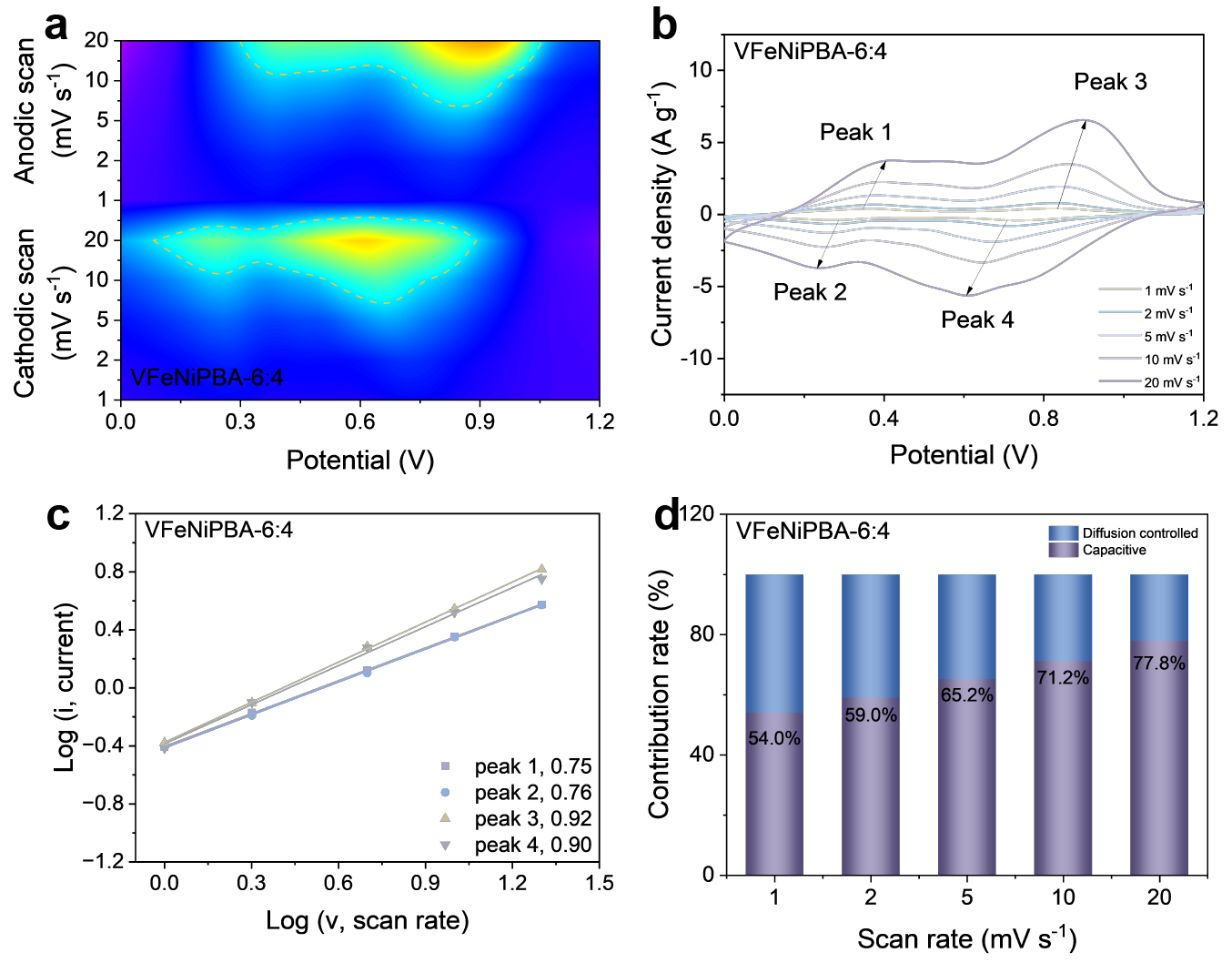


**Figure S42.** (a, b) CV curves at various scan rates, (c) The linear fitting plots of the b values from CV curves, (d) Capacitive Contribution Ratio at Different Scan Rates of VFeNiPBA-6:4.

# 44. Capacitive Contribution Ratio at different scan rates of VFeNiPBA-6:4


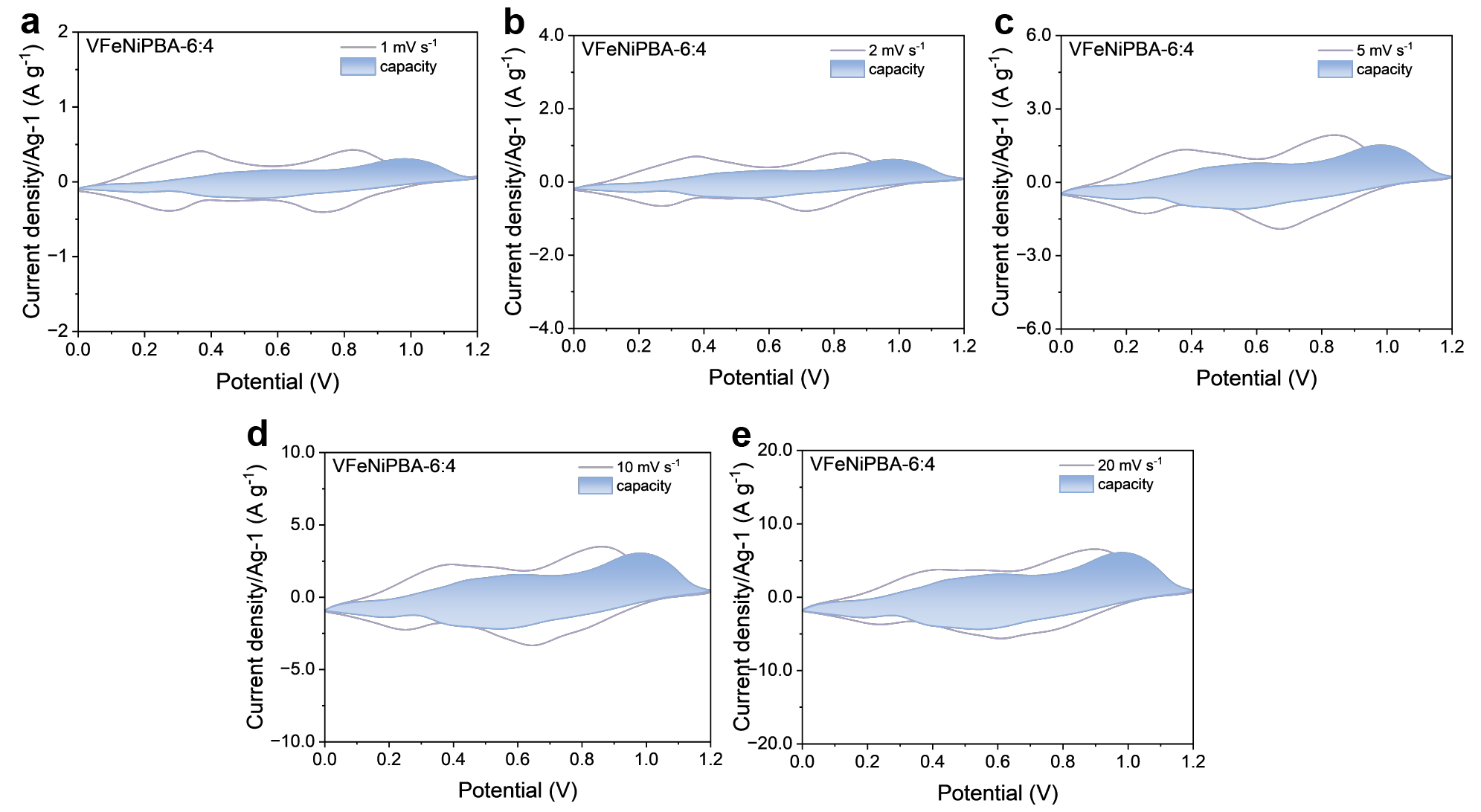


**Figure S43.** Capactitive contribution to total storage for the VFeNiPBA-6:4 electrode at different scan rates: (a) 1 mV·s^-1^, (b) 2 mV·s^-1^, (c) 5 mV·s^-1^, (d) 10 mV·s^-1^, and (e) 20 mV·s^-1^.

# 45. CV curves, b values, and Capacitive Contribution Ratio of VFeNiPBA-4:6


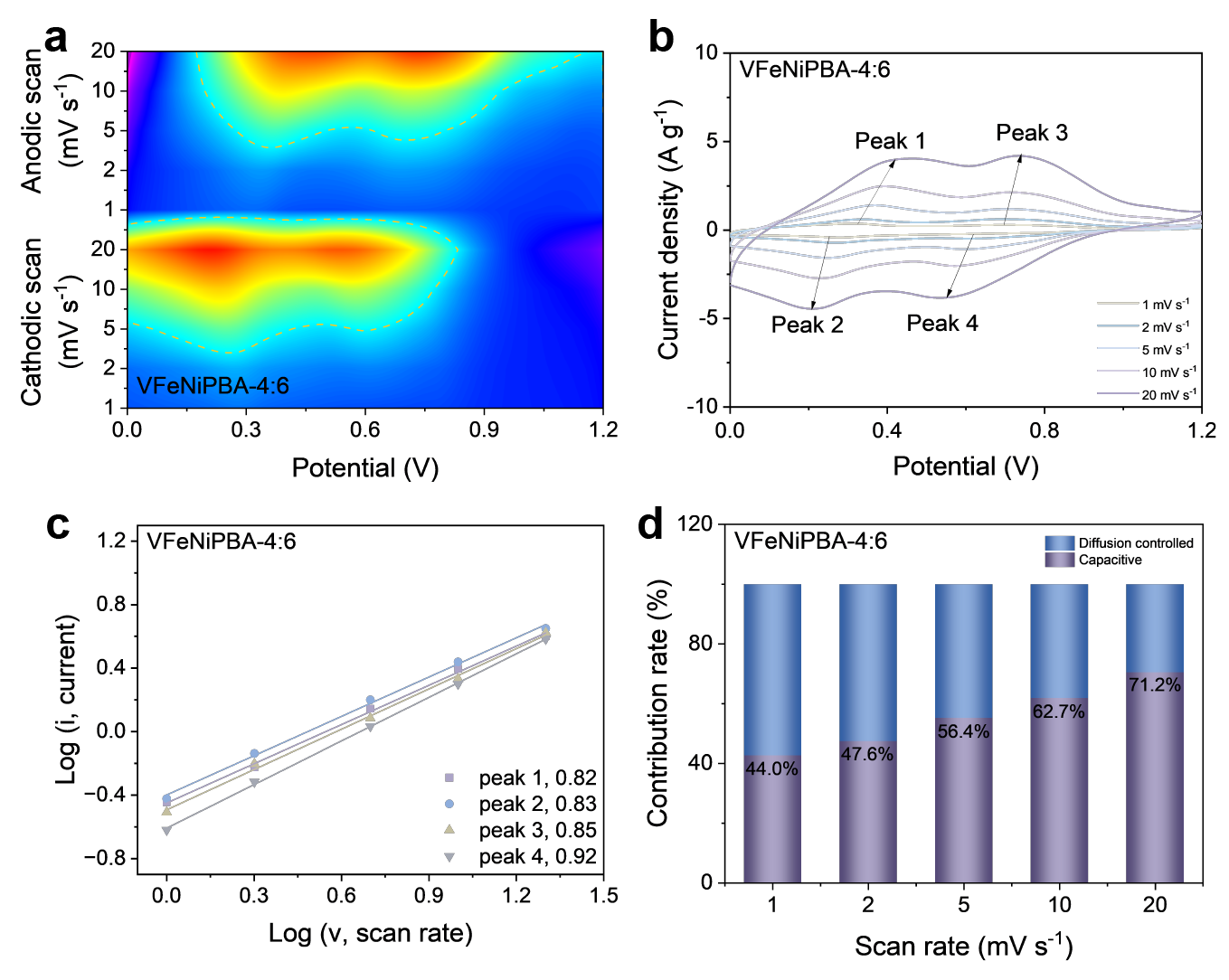


**Figure S44.** (a, b) CV curves at various scan rates, (c) The linear fitting plots of the b values from CV curves, (d) Capacitive Contribution Ratio at Different Scan Rates of VFeNiPBA-4:6.

# 46. Capacitive Contribution Ratio at different scan rates of VFeNiPBA-4:6


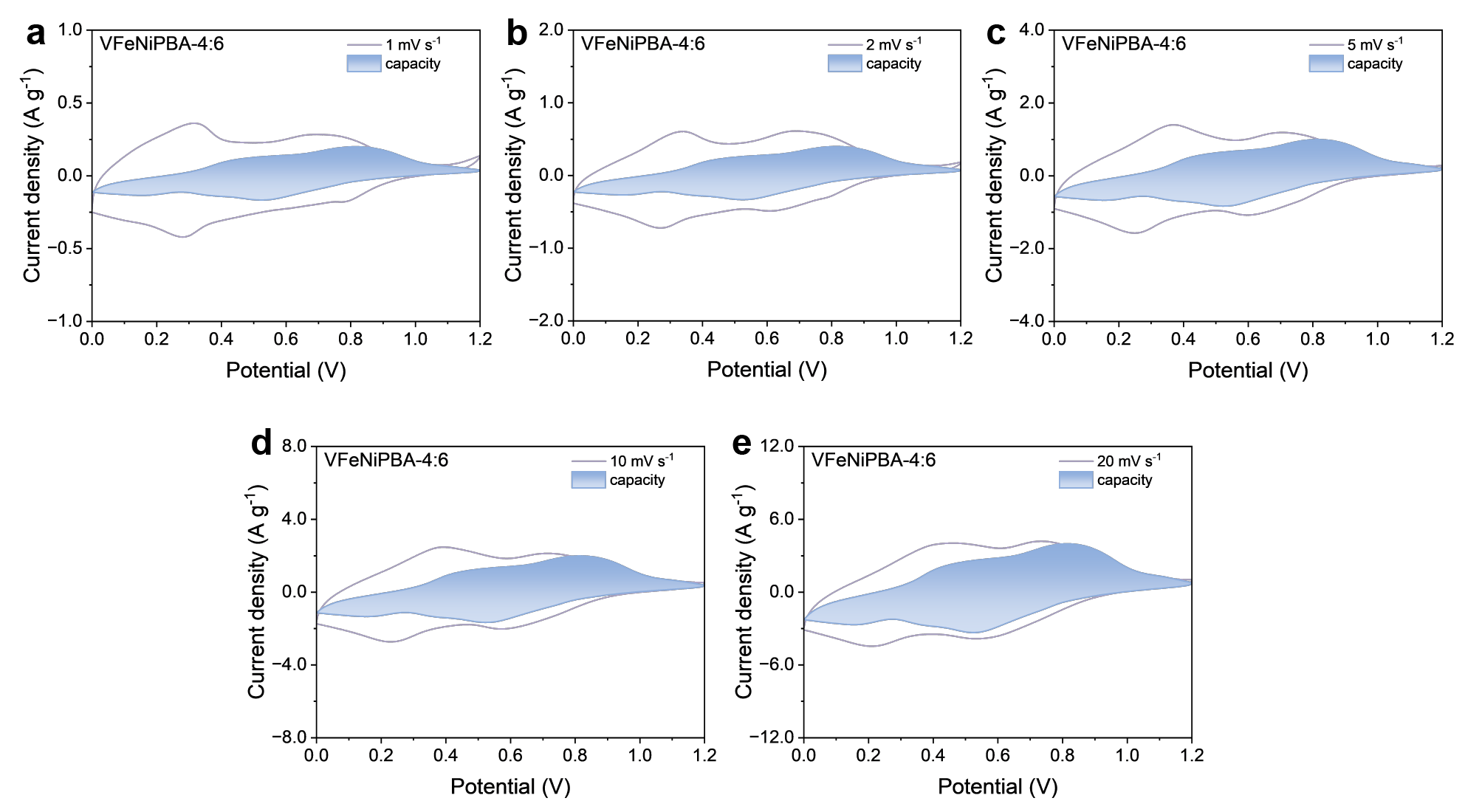


**Figure S45.** Capactitive contribution to total storage for the VFeNiPBA-4:6 electrode at different scan rates: (a) 1 mV·s^-1^, (b) 2 mV·s^-1^, (c) 5 mV·s^-1^, (d) 10 mV·s^-1^, and (e) 20 mV·s^-1^.

# 47. CV curves, b values, and Capacitive Contribution Ratio of VFeNiPBA-2:8


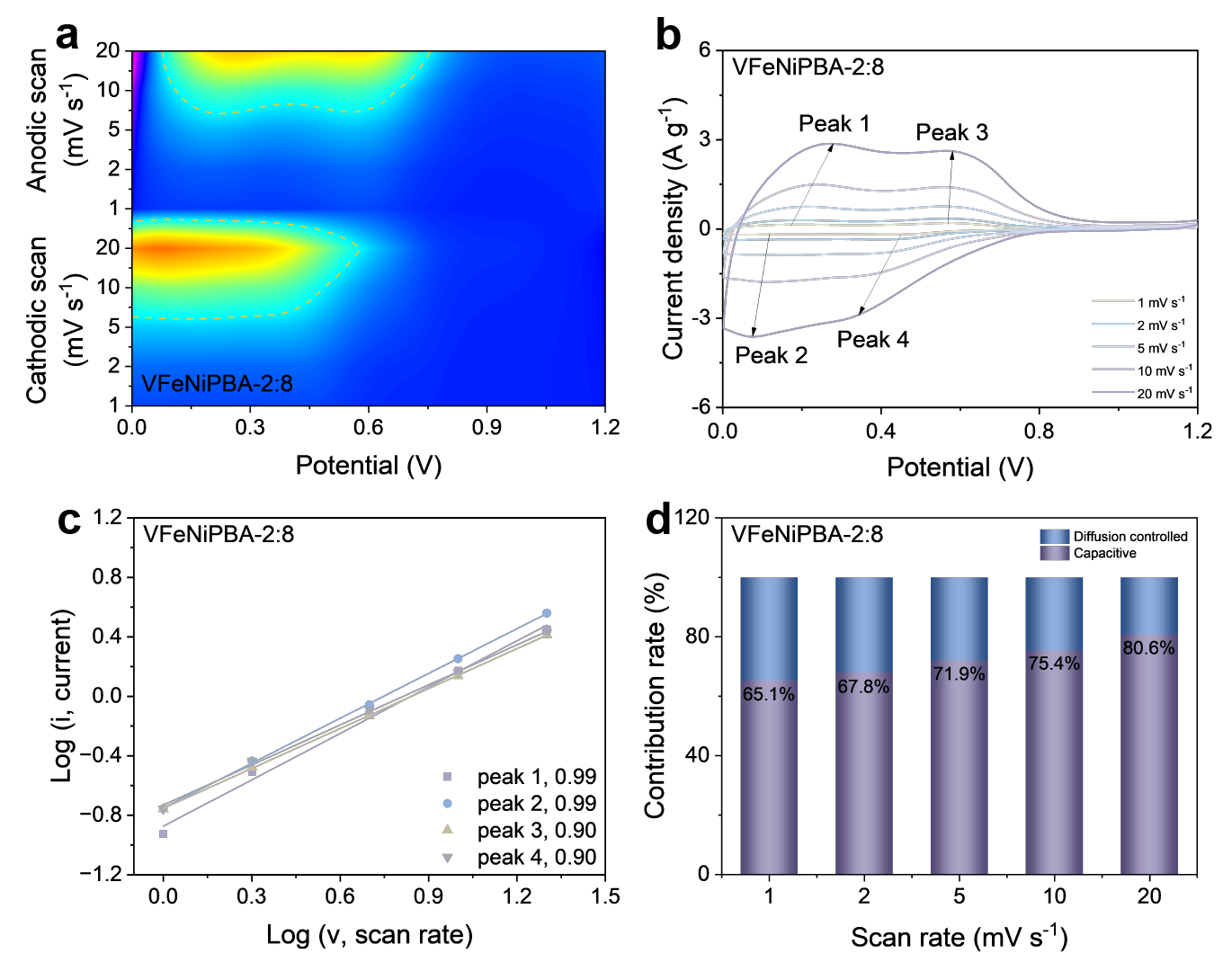


**Figure S46.** (a, b) CV curves at various scan rates, (c) The linear fitting plots of the b values from CV curves, (d) Capacitive Contribution Ratio at Different Scan Rates of VFeNiPBA-2:8.

# 48. Capacitive Contribution Ratio at different scan rates of VFeNiPBA-2:8


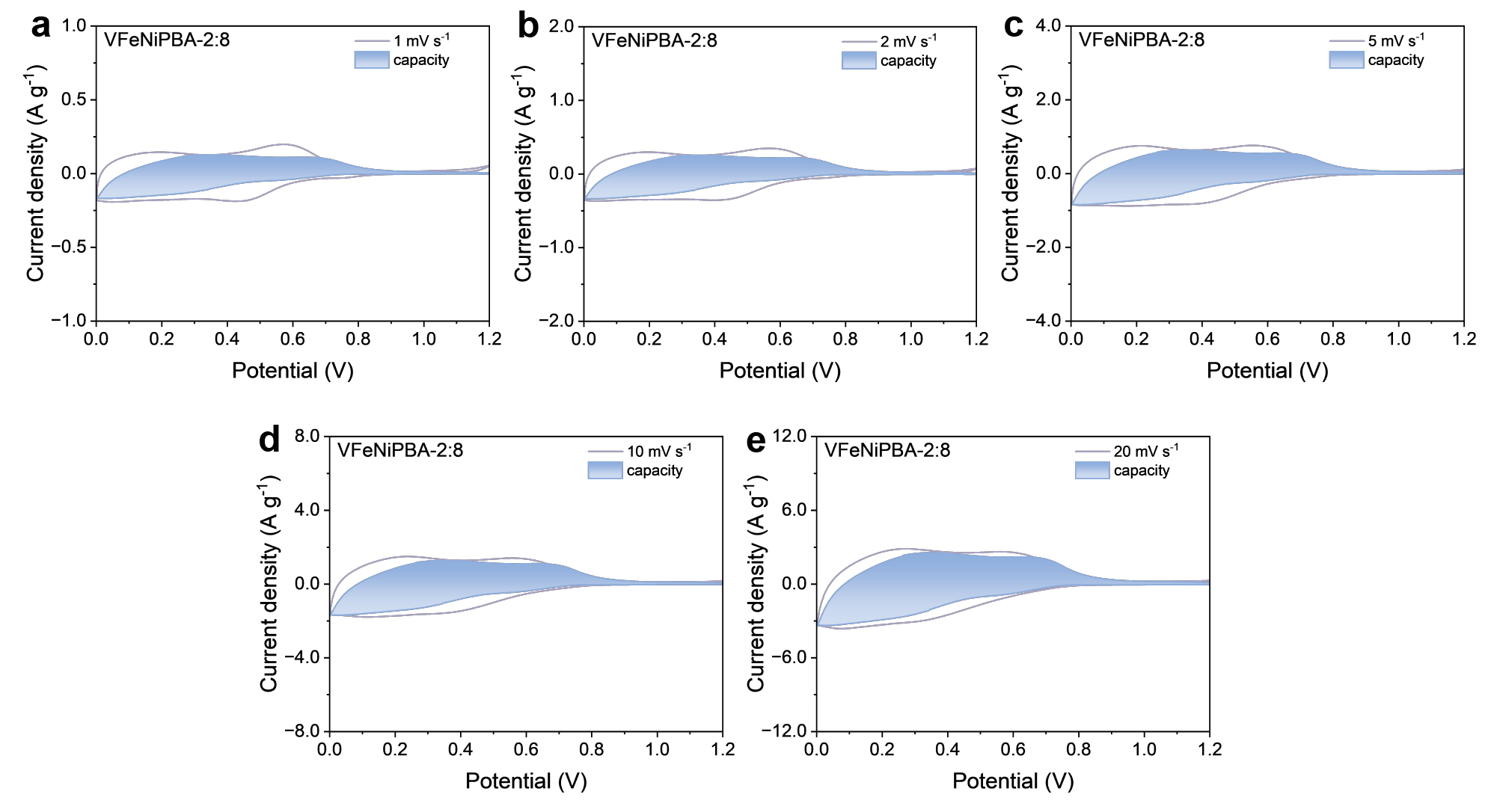


**Figure S47.** Capactitive contribution to total storage for the VFeNiPBA-2:8 electrode at different scan rates: (a) 1 mV·s^-1^, (b) 2 mV·s^-1^, (c) 5 mV·s^-1^, (d) 10 mV·s^-1^, and (e) 20 mV·s^-1^.

# 49. Rate performance of VFeNiPBA-8:2


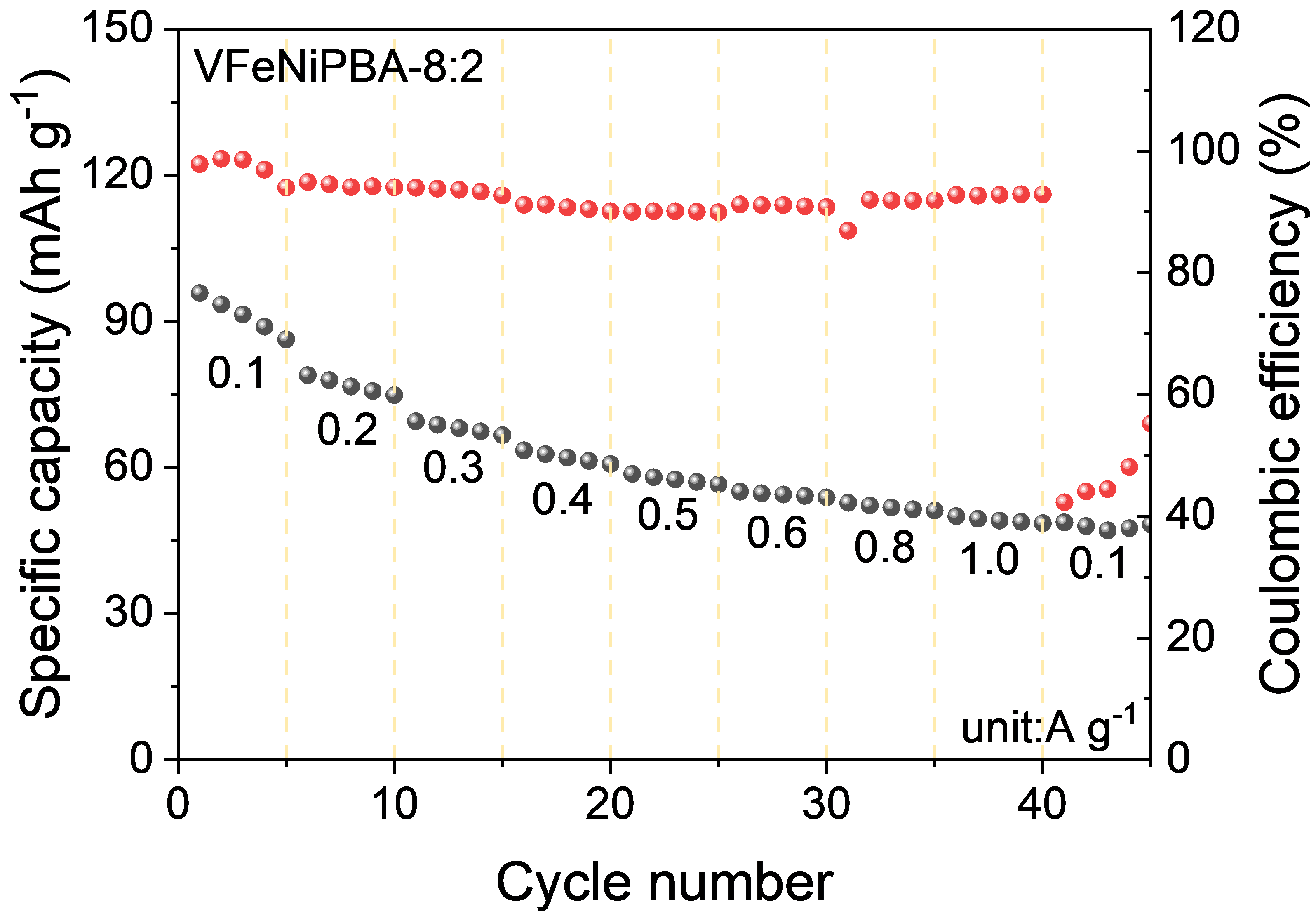


**Figure S48.** Rate performance of VFeNiPBA-8:2.

# 50. Rate performance of VFeNiPBA-6:4


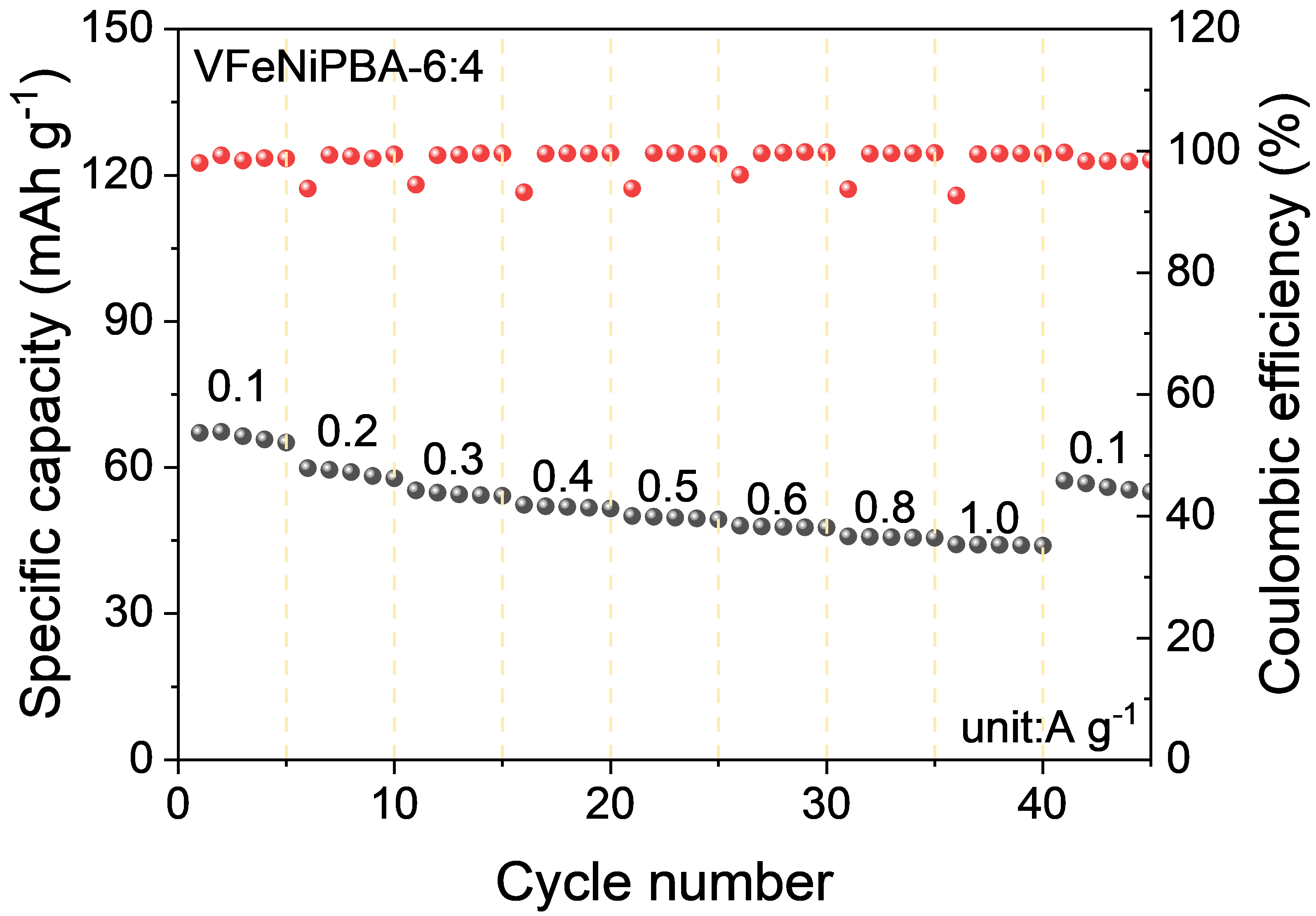


**Figure S49.** Rate performance of VFeNiPBA-6:4.

# 51. Rate performance of VFeNiPBA-4:6


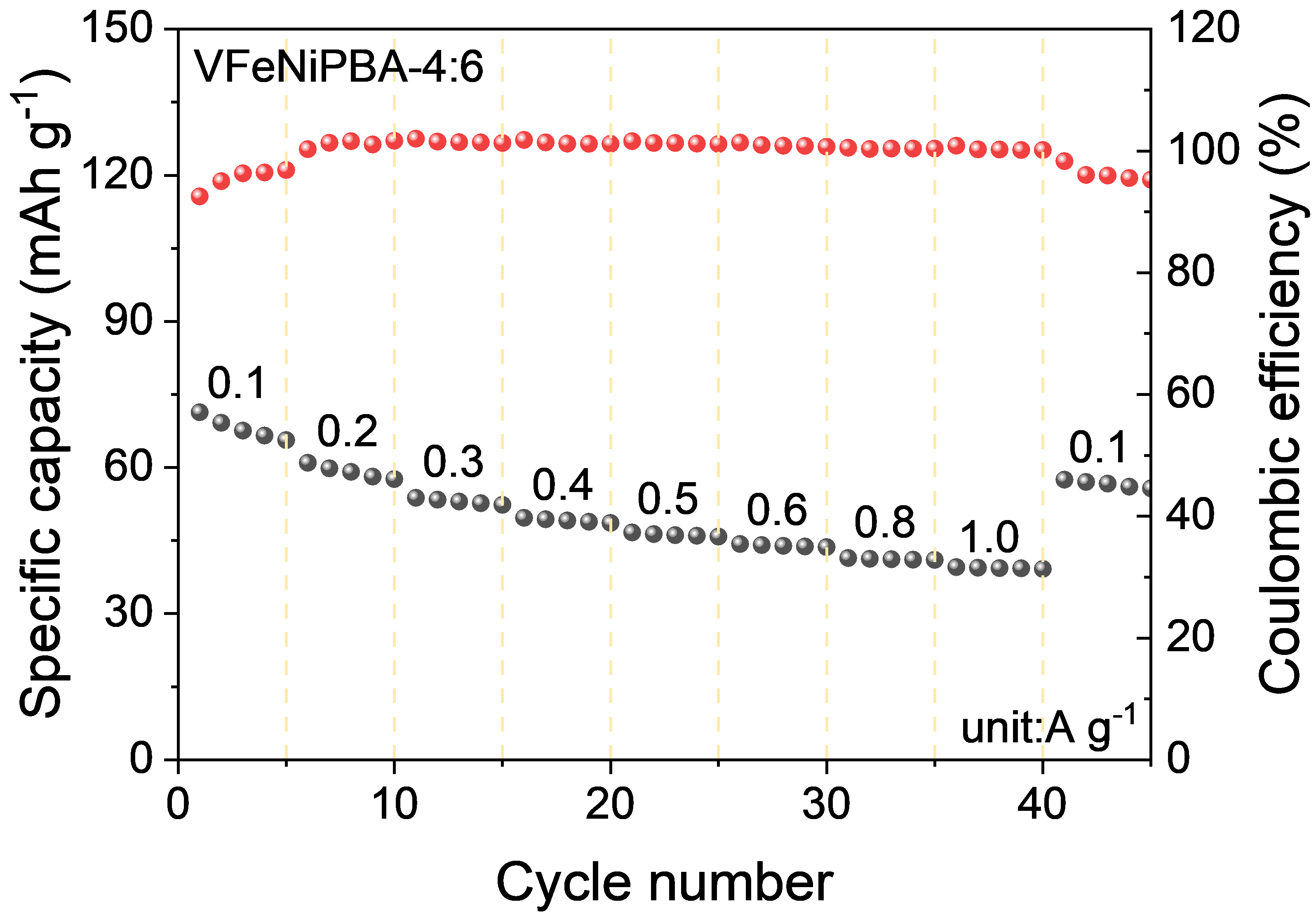


**Figure S50.** Rate performance of VFeNiPBA-4:6.

# 52. CV curves, b values, and Capacitive Contribution Ratio of VFePBA-25^o^C


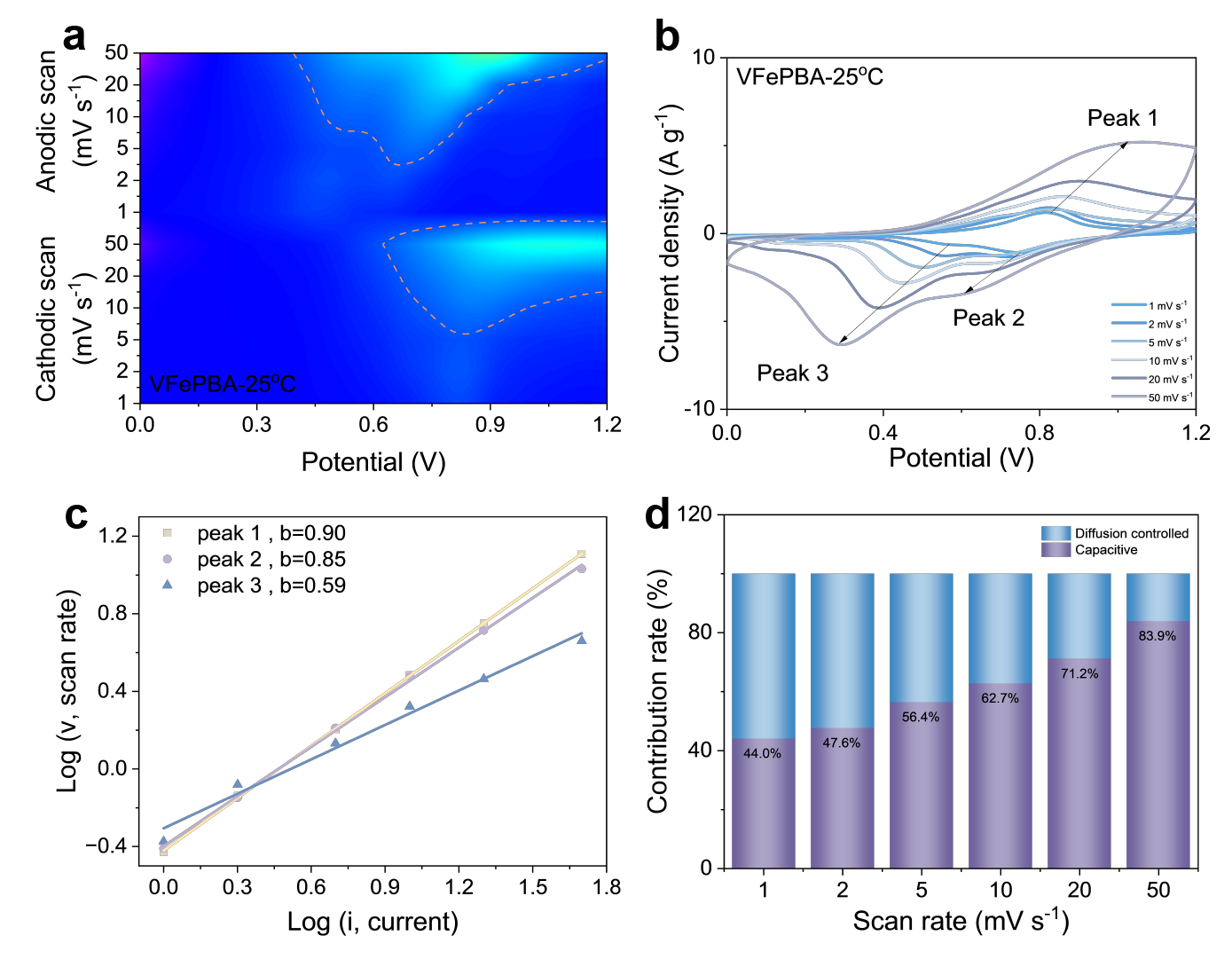


**Figure S51.** (a, b) CV curves at various scan rates, (c) The linear fitting plots of the b values from CV curves, (d) Capacitive Contribution Ratio at Different Scan Rates of VFePBA-25^o^C.

# 53. Capacitive Contribution Ratio at different scan rates of VFePBA-25^o^C


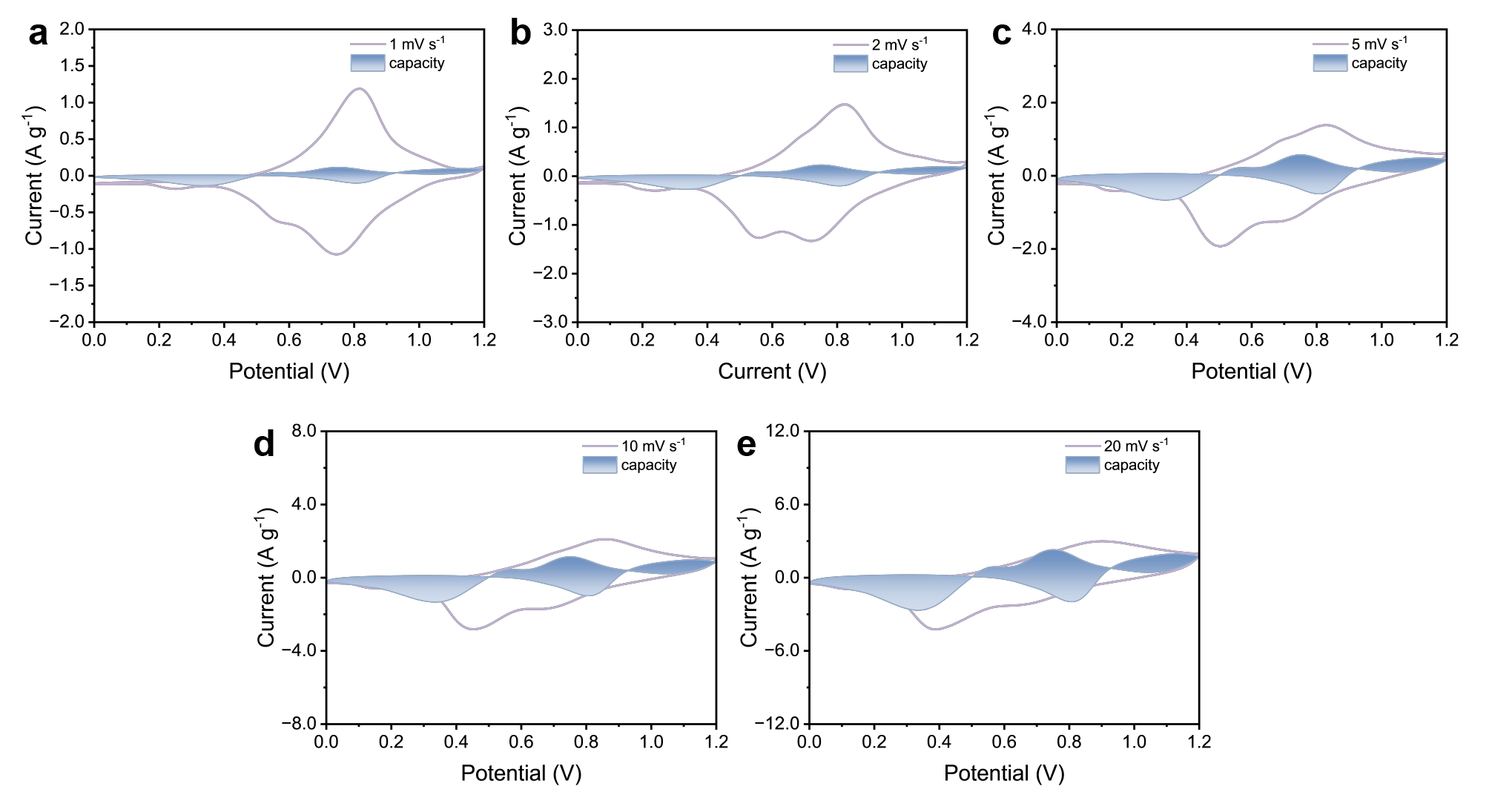


**Figure S52.** Capactitive contribution to total storage for the VFePBA-25^o^C electrode at different scan rates: (a) 1 mV·s^-1^, (b) 2 mV·s^-1^, (c) 5 mV·s^-1^, (d) 10 mV·s^-1^, and (e) 20 mV·s^-1^.

# 54. Cycling performance of VFePBA


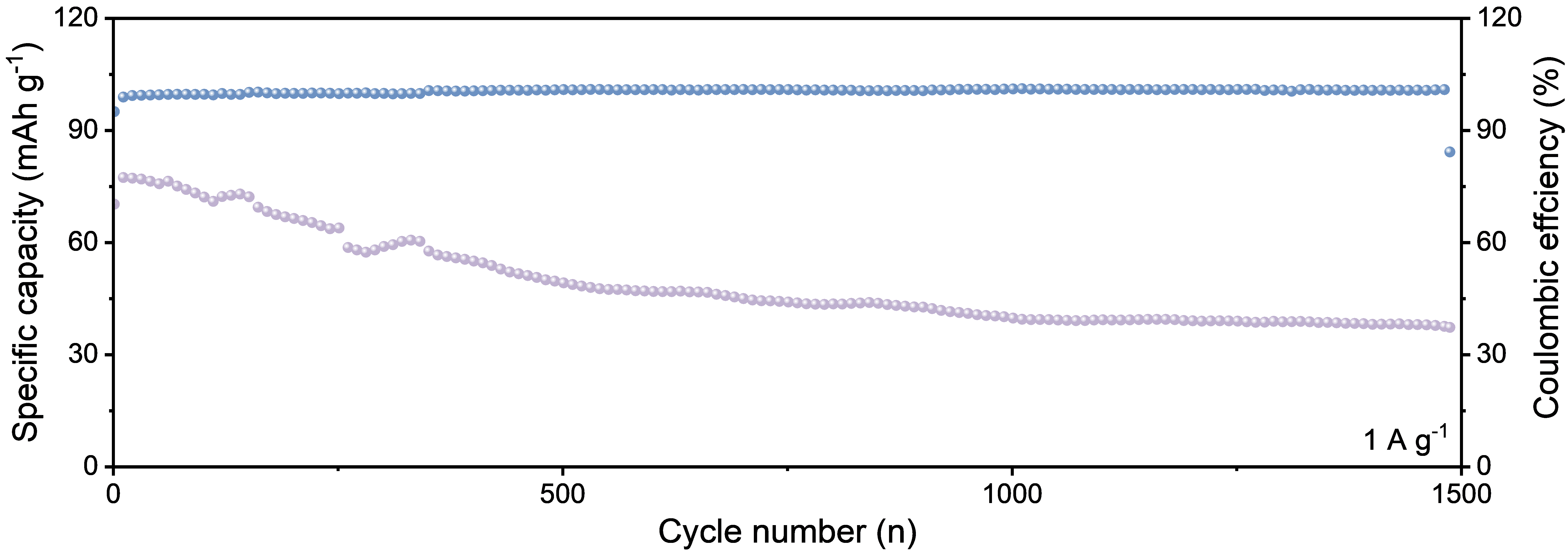


**Figure S53.** Cycling performance of VFePBA.

# 55. Charge-discharge profiles of VFePBA


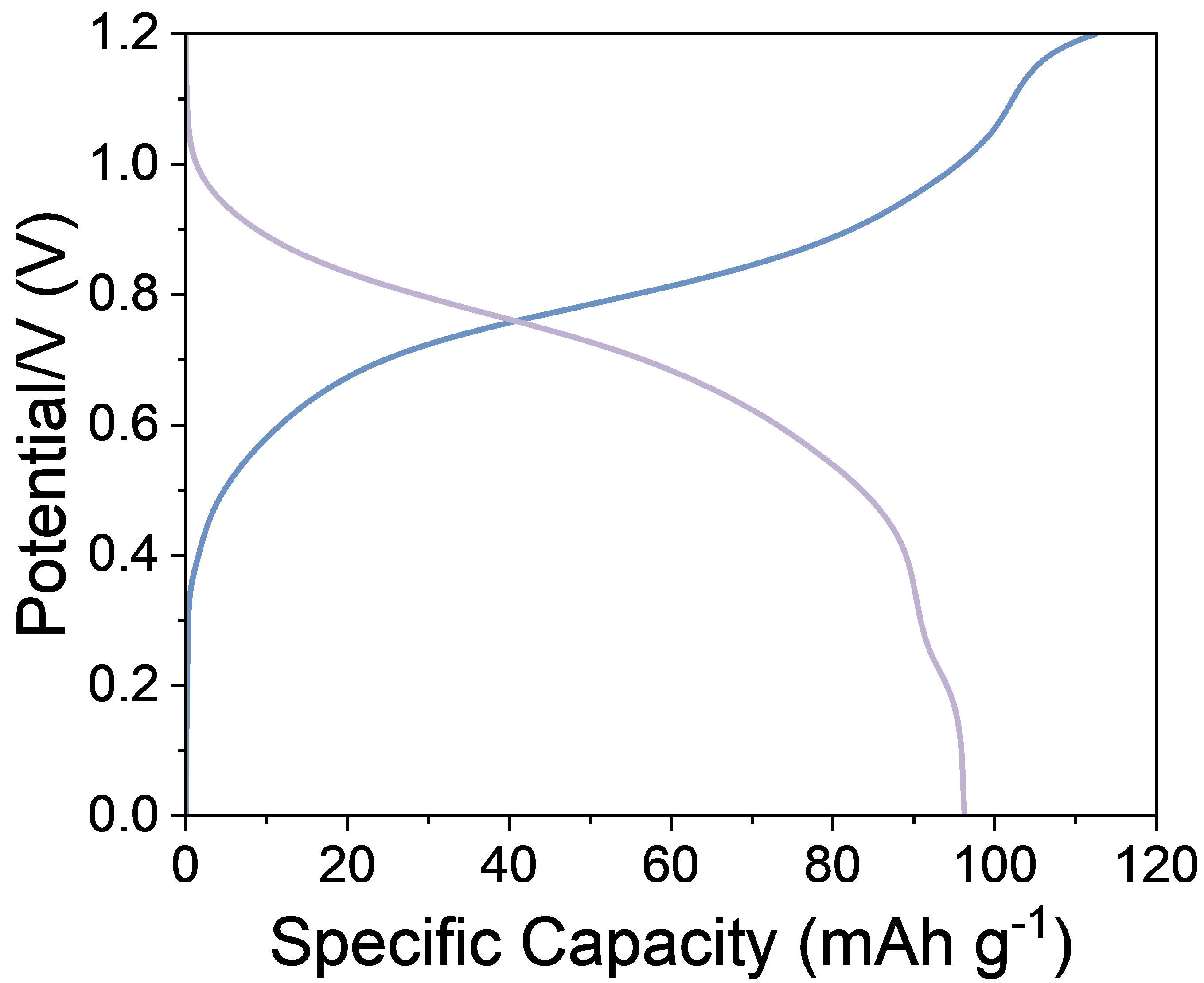


**Figure S54.** Charge-discharge profiles of VFePBA.

# 56. Cycling performance of VFePBA-25**o**C


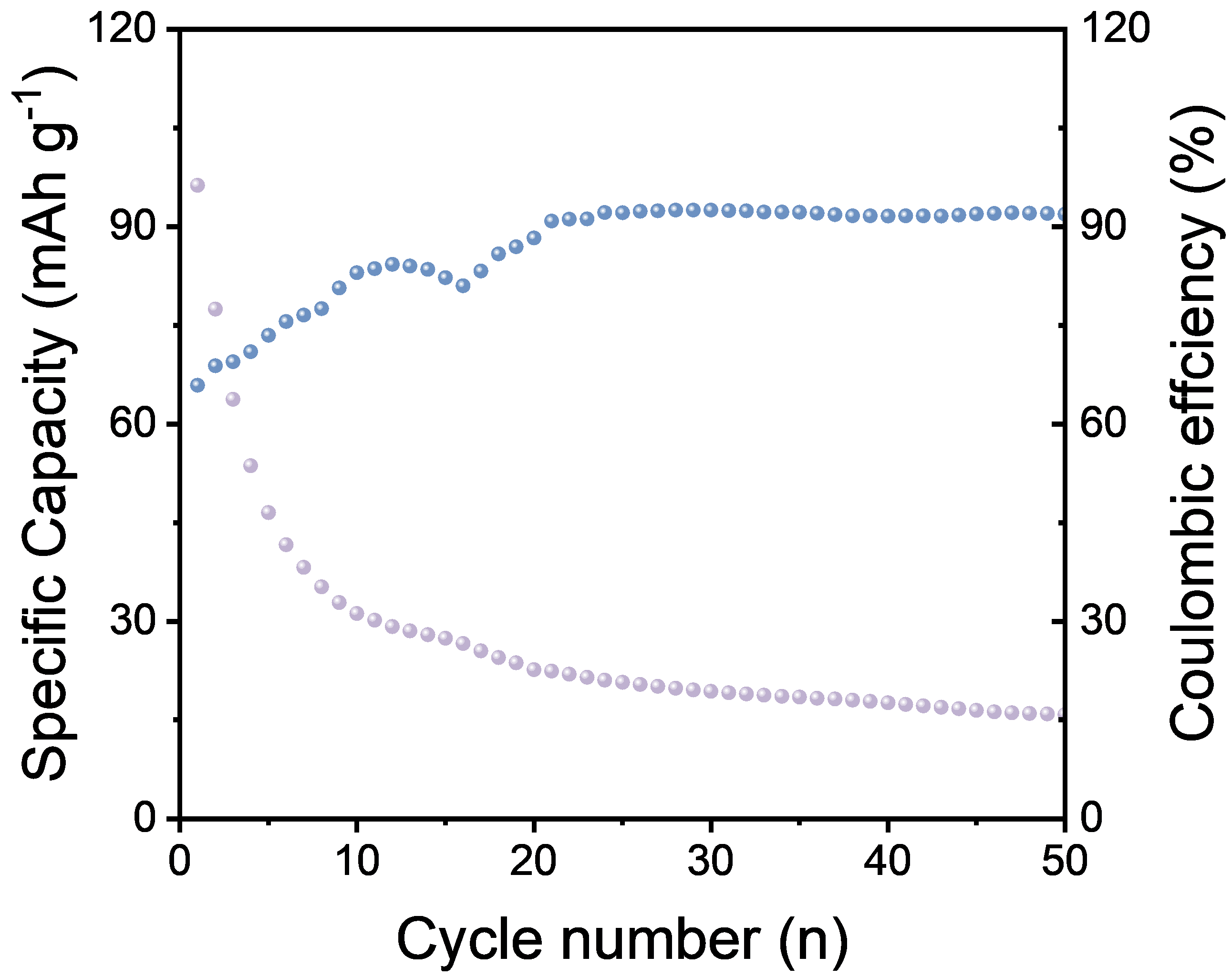


**Figure S55.** Cycling performance of VFePBA-25^o^C.

# 57. In-situ EIS results of VFePBA


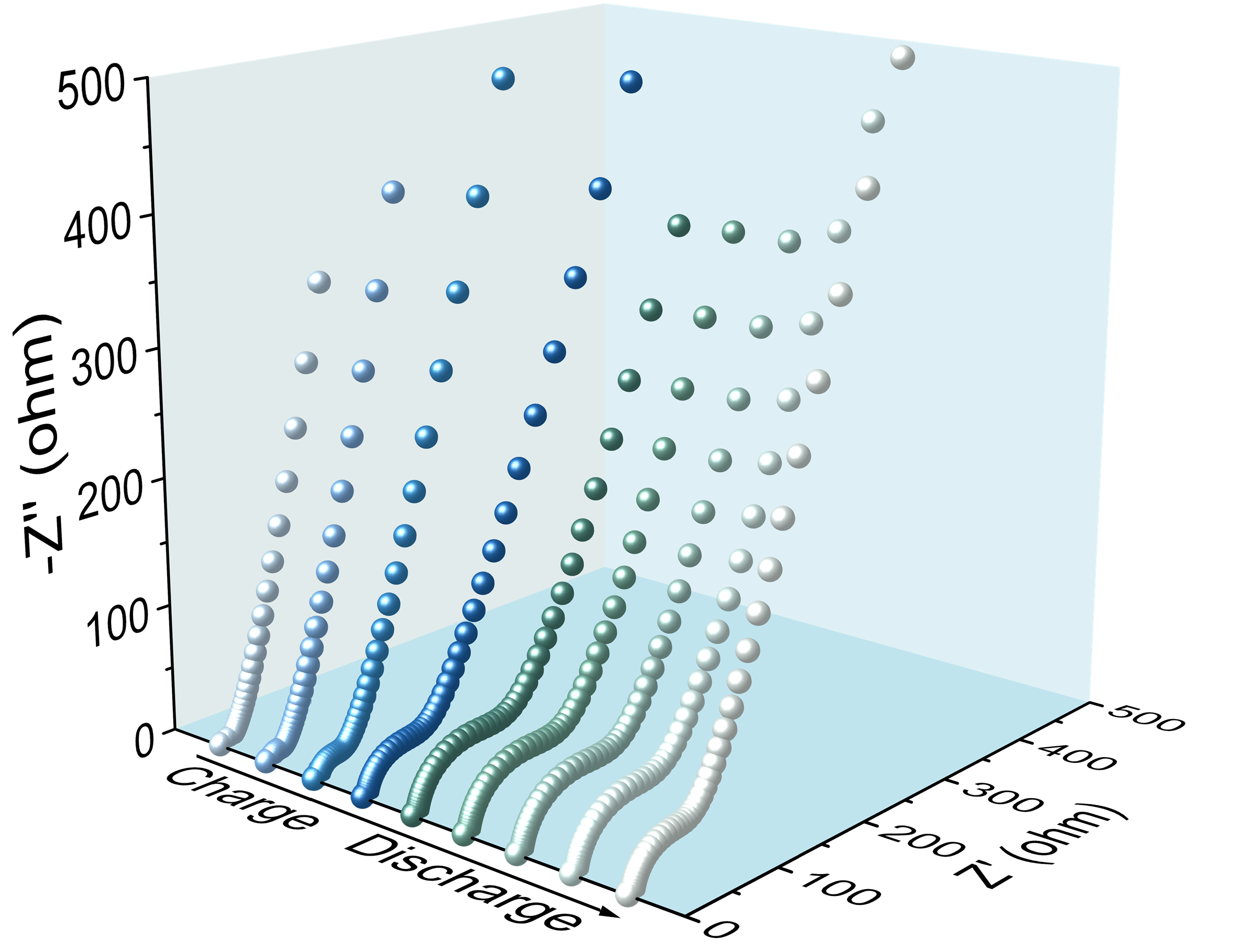


**Figure S56.** In-situ EIS results of VFePBA.

# 58. Cycling performance in different aqueous electrolytes of VFePBA


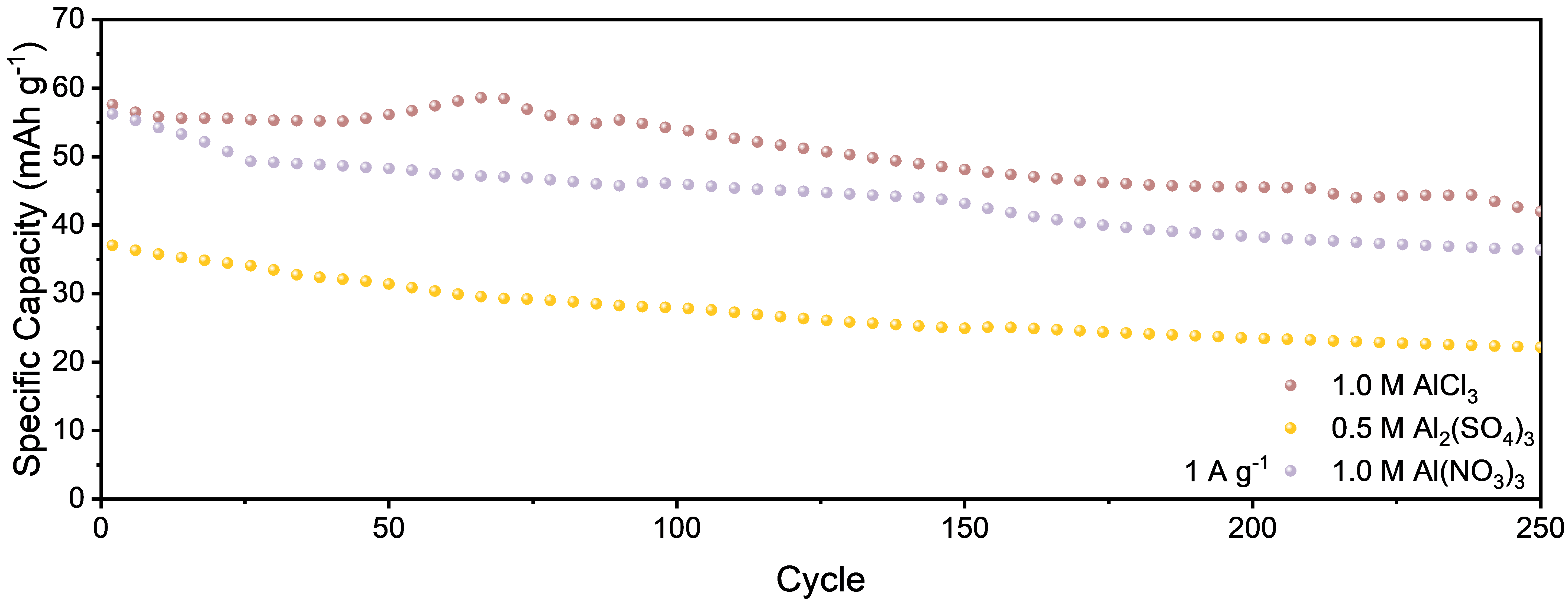


**Figure S57.** Cycling performance in different electrolytes of VFePBA.

# 59. Cycling performance of VFePBA in Al||1 M Al(OTF)_3_ + 0.2 M Mn(OTF)_2_||VFePBA


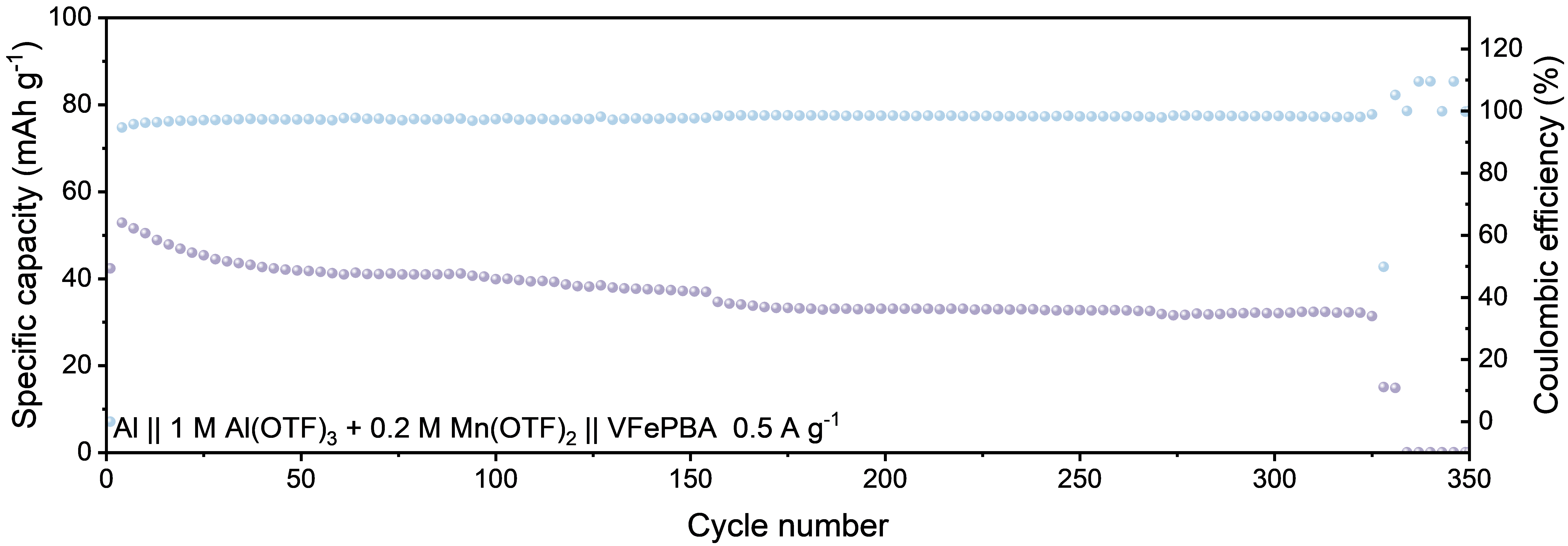


**Figure S58.** Cycling performance of VFePBA in Al||1 M Al(OTF)_3_ + 0.2 M Mn(OTF)_2_||VFePBA.

# 60. Cycling performance of VFePBA in Zn||1 M Al(OTF)_3_ + 0.2 M Mn(OTF)_2_||VFePBA


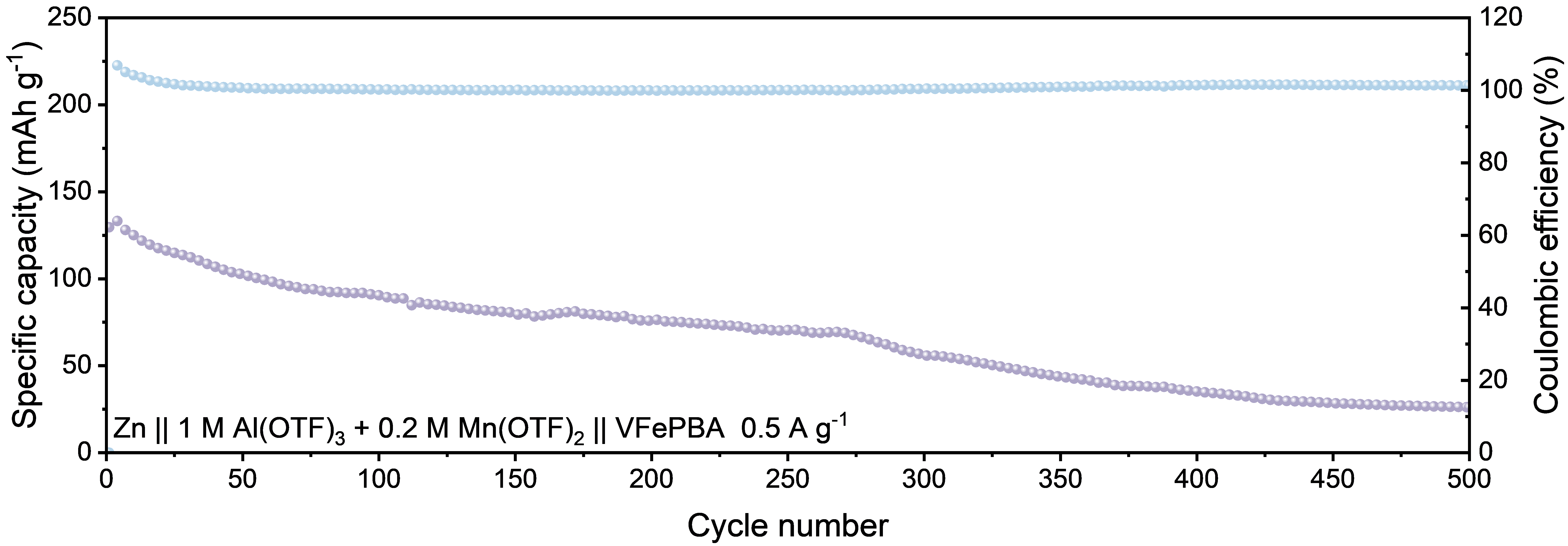


**Figure S59.** Cycling performance of VFePBA in Zn || 1 M Al(OTF)_3_ + 0.2 M Mn(OTF)_2_ || VFePBA.

# 61. CV curves, b values, and Capacitive Contribution Ratio of Zn||AU15||VFePBA


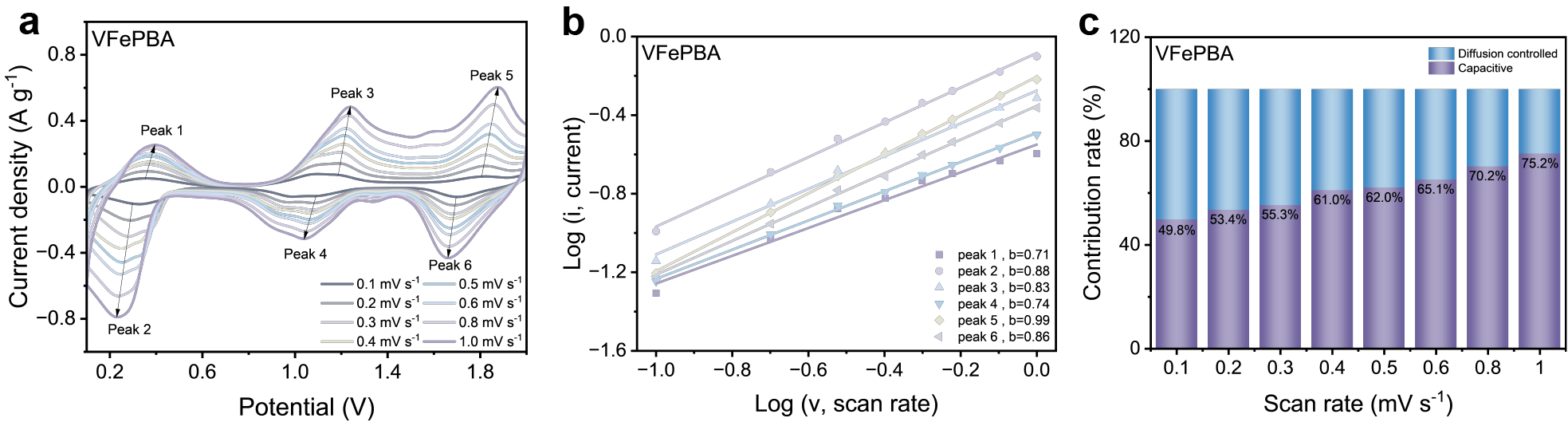


**Figure S60.** (a) CV curves at various scan rates, (b) The linear fitting plots of the b values from CV curves, (c) Capacitive Contribution Ratio at Different Scan Rates of Zn||AU15||VFePBA.

# 62. Capacitive Contribution Ratio at different scan rates of Zn||AU15||VFePBA


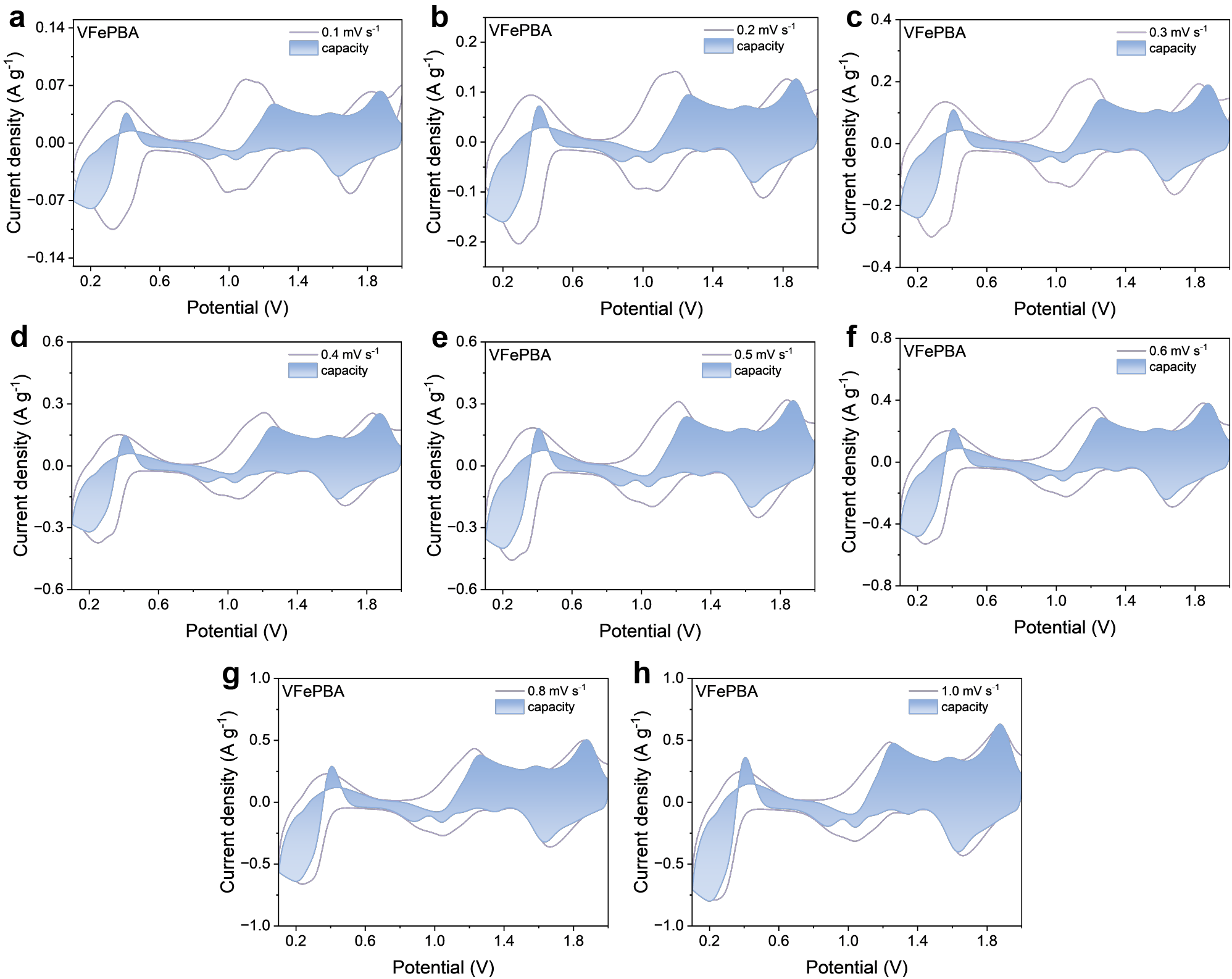


**Figure S61.** Capactitive contribution to total storage for the Zn||AU15||VFePBA at different scan rates: (a) 0.1 mV·s^-1^, (b) 0.2 mV·s^-1^, (c) 0.3 mV·s^-1^, (d) 0.4 mV·s^-1^, (e) 0.5 mV·s^-1^, (f) 0.6 mV·s^-1^, (g) 0.8 mV·s^-1^, and (h) 1 mV·s^-1^.

# 63. EIS and Equivalent Circuit Diagram of the Zn||AU15||VFePBA System


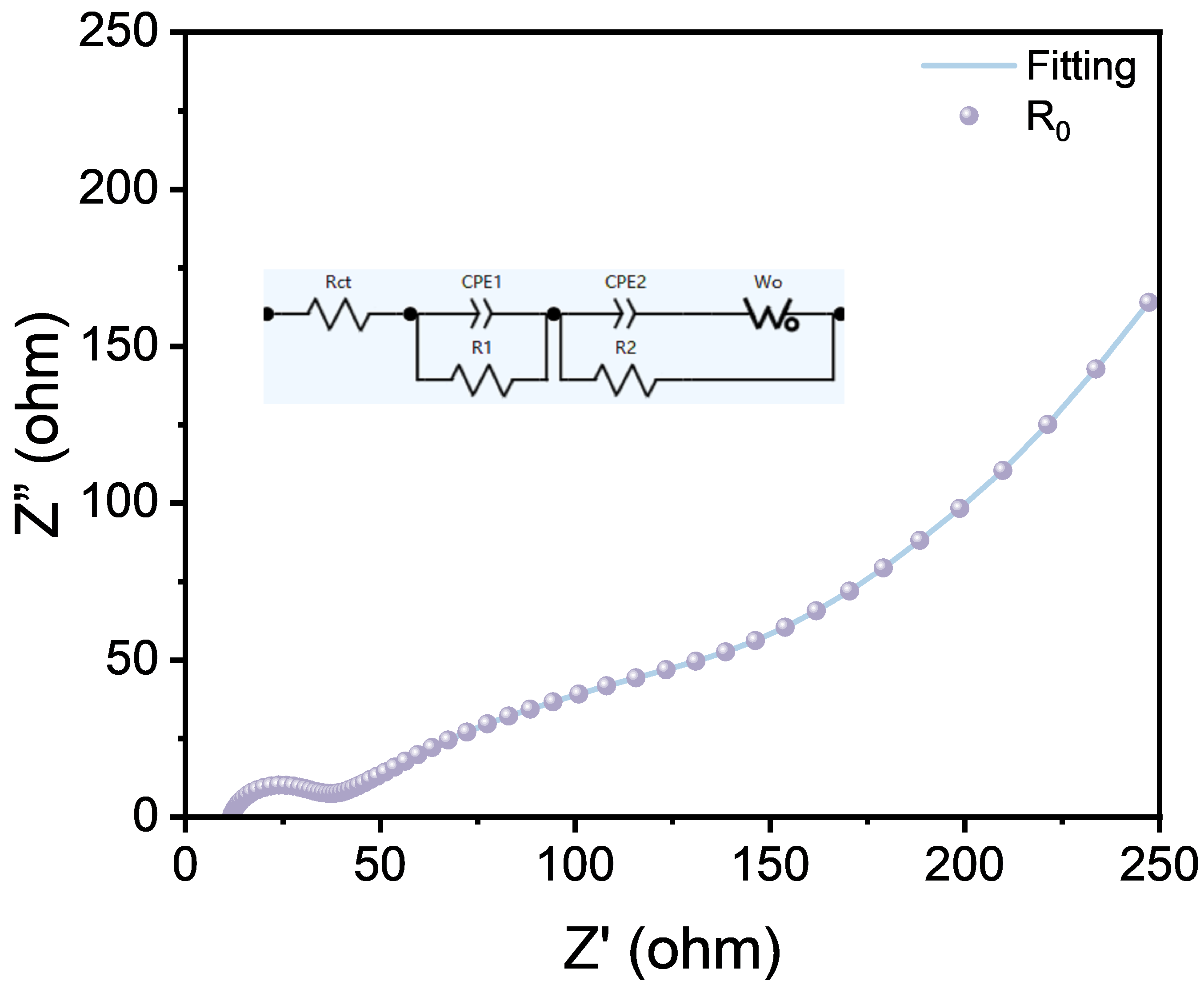


**Figure S62**. Electrochemical Impedance Spectroscopy and Equivalent Circuit Diagram of the Zn||AU15||VFePBA System

# 64. Contact angle between VFePBA and electrolyte


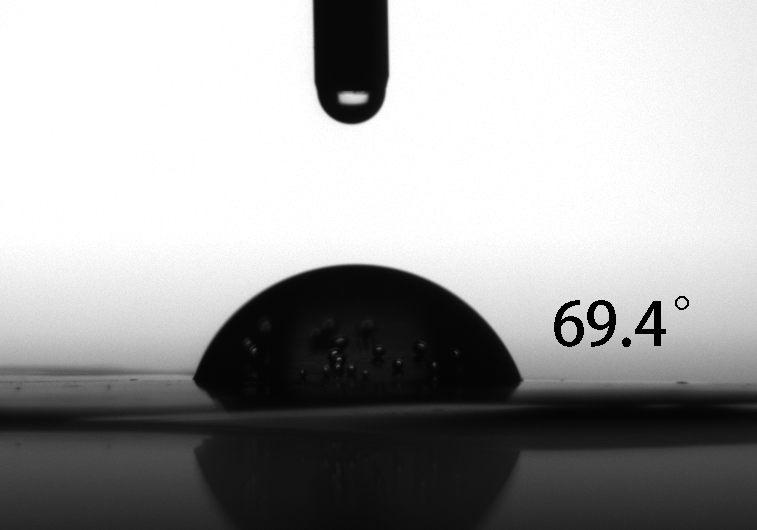


**Figure S63**. Contact angle between VFePBA and electrolyte

# 65. Charge-discharge profiles of Pouch cell demonstration of VFePBA


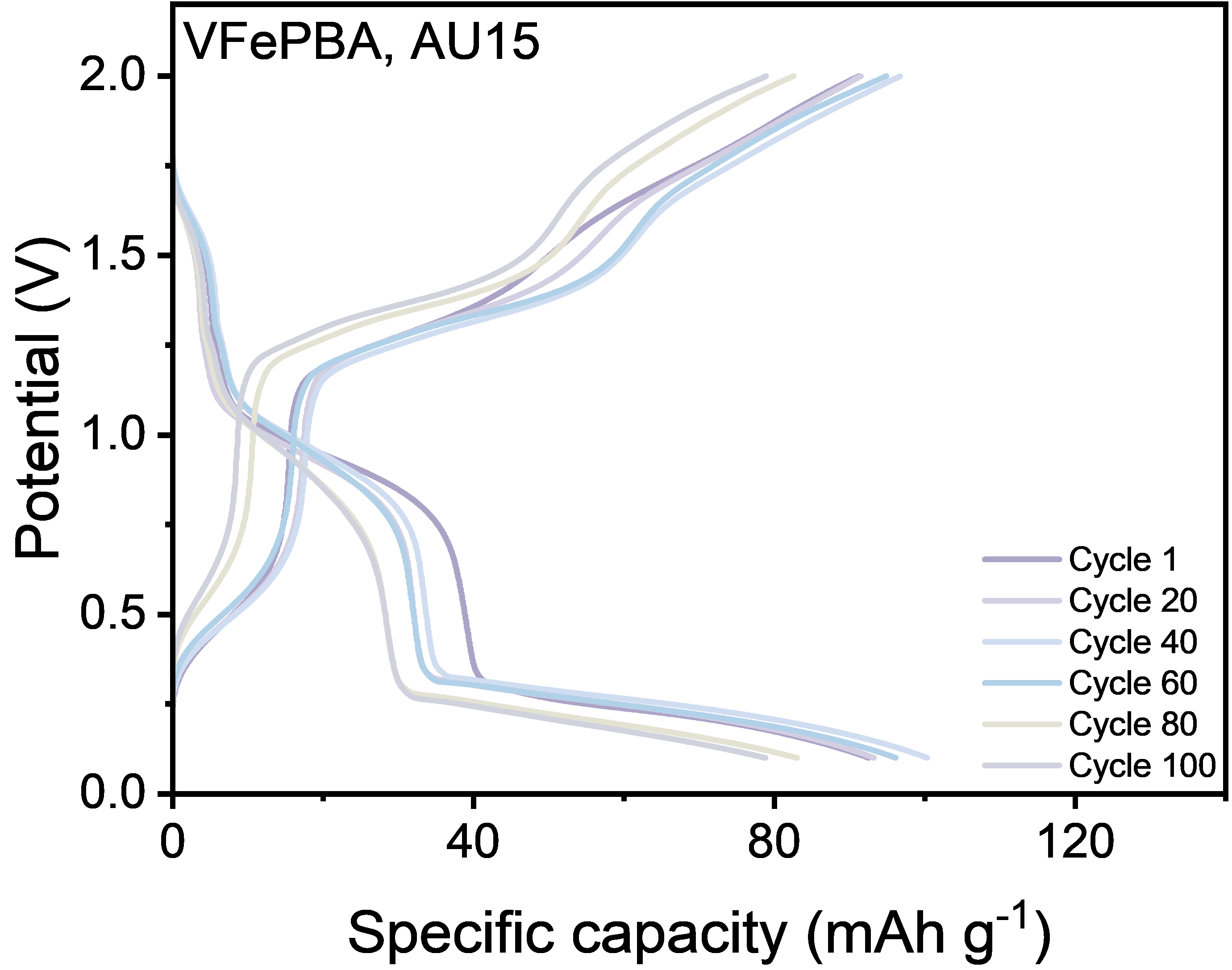


**Figure S64.** Charge-discharge profiles of Pouch cell demonstration of VFePBA

# 66. Structure of VFePBA before Al^3+^ intercalation


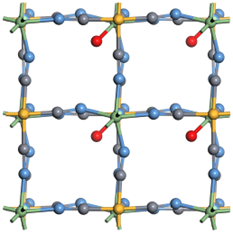


**Figure S65.** Structure of VFePBA before Al^3+^ intercalation.

# 67. Charge distribution on the (101) crystal plane of VFePBA


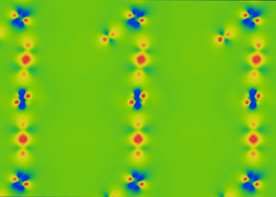


**Figure S66.** Charge distribution on the (101) crystal plane of VFePBA

# 68. Structure of VFePBA after Al^3+^ intercalation


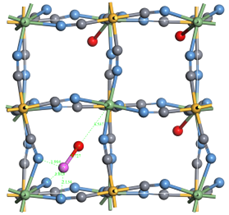


**Figure S67.** Structure of VFePBA after Al^3+^ intercalation.

# 69. Ex situ XPS spectra of VFePBA: Zn 2p


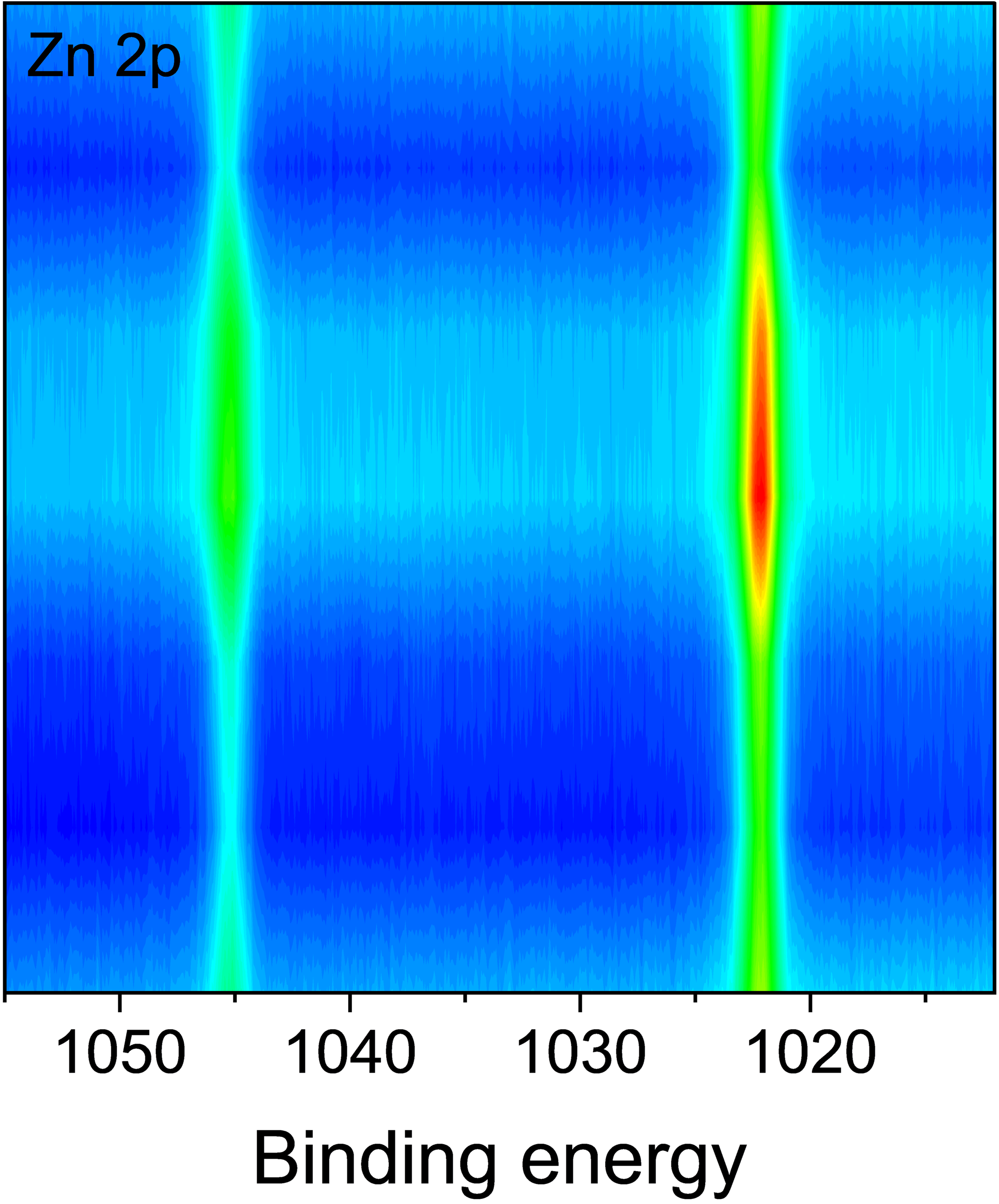


**Figure S68.** Ex situ XPS spectra of VFePBA: Zn 2p.

# 70. In-situ XRD patterns of VFePBA in Zn||AU15||VFePBA


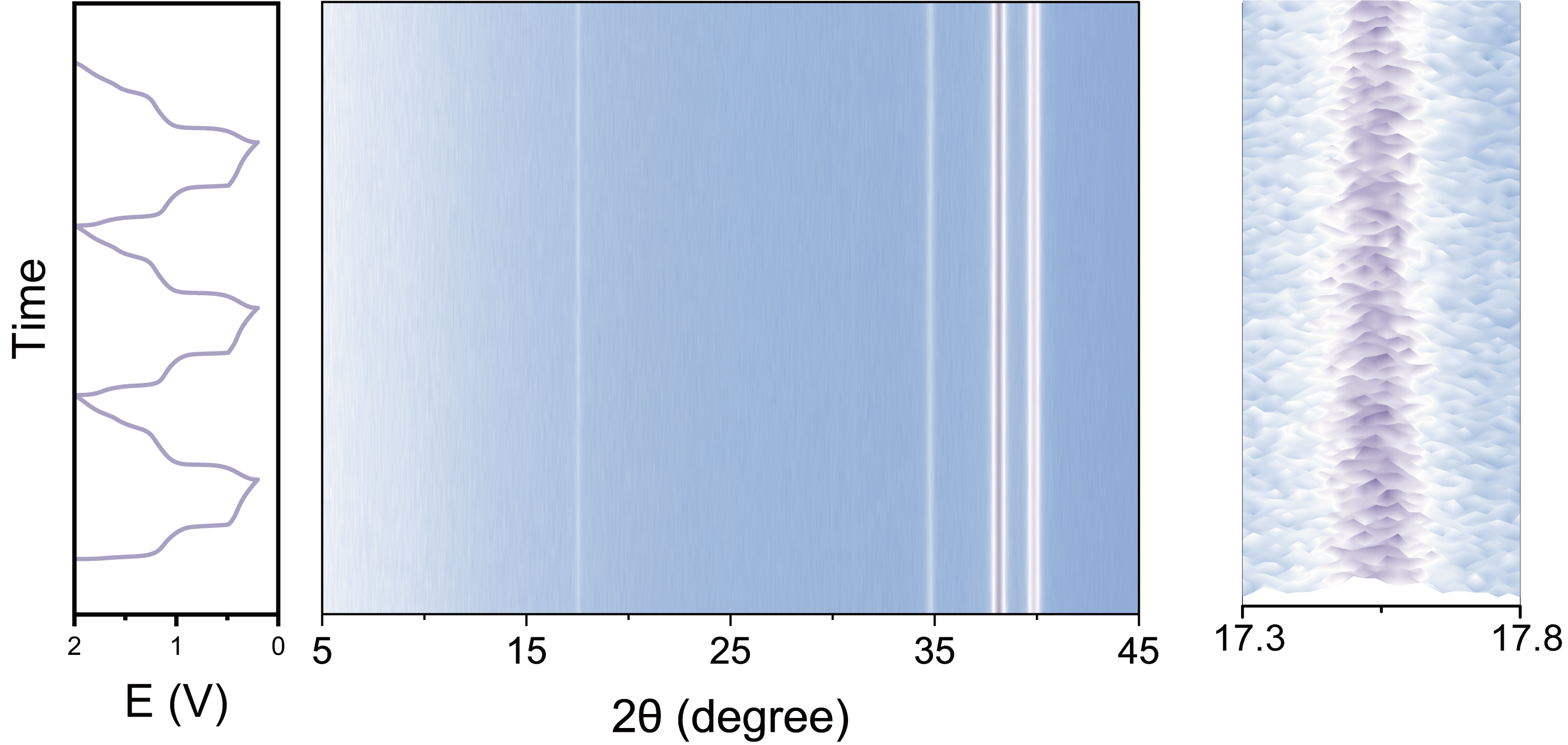


**Figure S69.** In-situ XRD patterns of VFePBA in Zn||AU15||VFePBA and corresponding charge/discharge curves.

# 71. In-situ UV spectroscopy of VFePBA in Zn||AU15||VFePBA


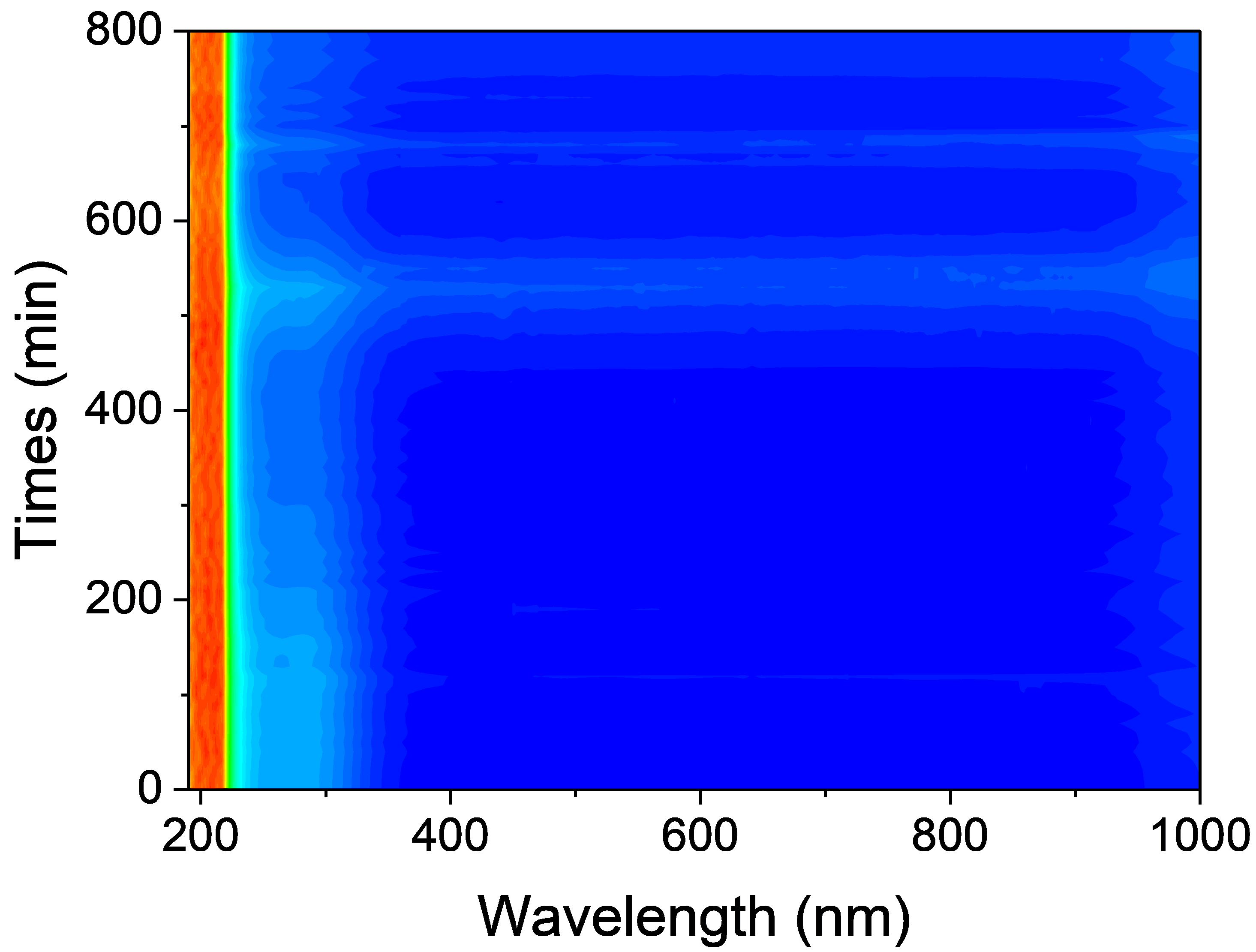


**Figure S70.** In-situ UV spectroscopy of VFePBA in Zn||AU15||VFePBA

# Rerfence

[1] J. Xing, X. Fu, S. Guan, Y. Zhang, M. Lei, Z. Peng, *Applied Surface Science* **2021**, *543*, 148843.

[2] C. Lu, Z. Wang, Y. Zhang, G. Tang, Y. Wang, X. Guo, J. Li, L. Wei, *Nano Energy* **2024**, *120*, 109158.

[3] J. P. Perdew, K. Burke, M. Ernzerhof, *Phys. Rev. Lett.*, **1996**, *77*, 3865-3868.

[4] B. Delley, *Phys. Rev. B.*, **2002**, *66*, 155125.

[5] T. A. Halgren, W. N. Lipscomb, *Chem. Phys. Lett*. **1977**, *49*, 225-232.

[6] N. Govind, M. Petersen, G. Fitzgerald, D. King-Smith, J. Andzelm, *Comput.* *Mater. Sci.* **2003**, *28*, 250-258.
